# Isotope-free mapping of protein-RNA interactions at single-nucleotide resolution by iCLIP3

**DOI:** 10.1016/j.xpro.2026.104704

**Published:** 2026-07-17

**Authors:** Vladimir Despic, Melina Klostermann, Anna Orekhova, Mikhail Mesitov, Anke Busch, Kathi Zarnack, Julian König, Michaela Müller-McNicoll

**Affiliations:** 1 Institute of Molecular Biosciences, Goethe University Frankfurt, 60438 Frankfurt Am Main, Germany; 2 Cluster of Excellence SubCellular Architecture of Life (SCALE), Goethe University Frankfurt, 60438 Frankfurt am Main, Germany; 3 Theodor Boveri Institute, Biocenter, University of Würzburg, 97074 Würzburg, Germany; 4 Institute of Molecular Biology (IMB), 55128 Mainz, Germany; 5 Max Planck Institute of Biophysics, 60438 Frankfurt Am Main, Germany; 6 Cluster for Nucleic Acid Sciences and Technologies – NUCLEATE, 81377 Munich, Germany

**Keywords:** Gene Expression, RNAseq, Sequence analysis

## Abstract

Individual-nucleotide resolution UV crosslinking and immunoprecipitation (iCLIP) enables transcriptome-wide mapping of RNA-binding protein (RBP)-RNA interactions. Here, we present iCLIP version 3 (iCLIP3), a streamlined protocol optimized for generating high-quality iCLIP libraries from low-input material. We describe steps for near-infrared visualization of RBP-RNA complexes, silica column-based RNA isolation, and unique dual indexing using TruSeq adapters for cost-effective multiplexing and sequencing. We detail a complete bioinformatics workflow to identify crosslinking events and define RBP binding sites from raw sequencing data.

## Highlights

• Instructions for near-infrared visualization of RBP-RNA complexes with pCp-IR750
• Protocol for RNA recovery from crosslinked RBP-RNA complexes using silica columns
• Steps for iCLIP3 library preparation with TruSeq adapters and unique dual indices
• Detailed iCLIP3 data analysis workflow using racoon_clip and BindingSiteFinder

## Abstract (editor highlights)

Publisher’s note: Undertaking any experimental protocol requires adherence to local institutional guidelines for laboratory safety and ethics.

## Graphical abstract

**Figure:**
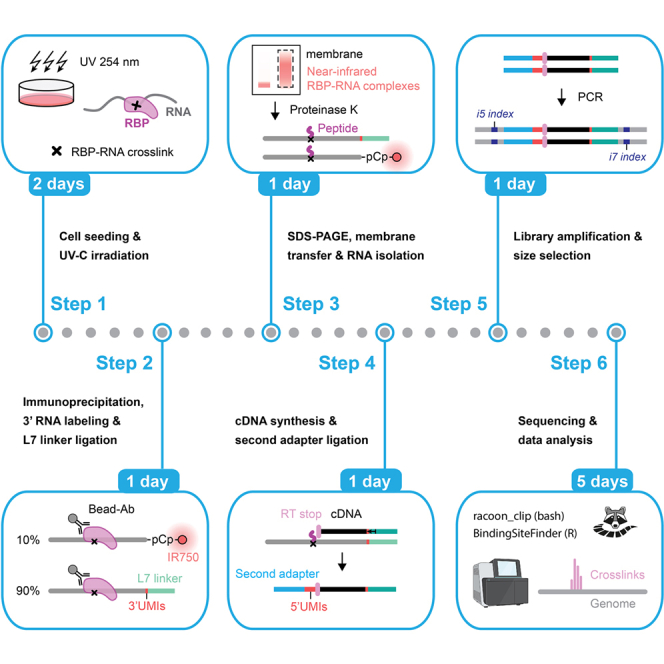

## Before you begin

### Protocol overview

UV-C crosslinking and immunoprecipitation (CLIP)-based techniques offer a powerful approach for transcriptome-wide mapping of RNA-binding protein (RBP)–RNA interactions at nucleotide resolution in living cells. These methods rely on UV-induced covalent crosslinking of RBPs to target RNAs, followed by immunoprecipitation (IP) of the crosslinked RBP-RNA complexes and identification of associated RNA fragments by high-throughput sequencing.^1^^,^^2^ In iCLIP, immunopurified RBP–RNA complexes are denatured, separated via SDS–PAGE, and transferred to a nitrocellulose membrane. RNA is isolated via protease digestion of the crosslinked RBP, but contains a short peptide at the crosslinked nucleotide. During reverse transcription, this residual peptide induces premature termination of cDNA synthesis at the crosslink site. Sequencing of these truncated cDNAs enables the precise, single-nucleotide resolution mapping of RBP binding sites across the transcriptome.^3^^,^^4^

### Protocol innovation

**Figure 1:**
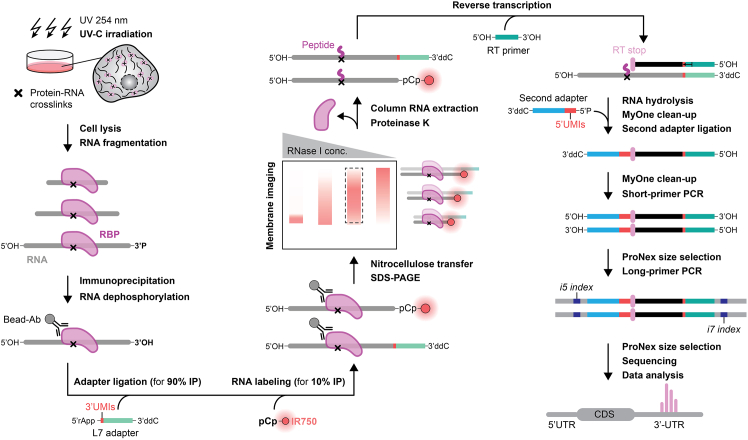
Overview of the iCLIP3 library preparation workflow Living cells are irradiated with 254-nm UV light to induce covalent crosslinking between RNA and RBPs, followed by cell lysis. RNA is partially fragmented with RNase I in cleared lysates of defined protein amount and concentration. The RBP of interest is then purified using a specific antibody coupled to magnetic Protein A or G beads. 3′ ends of the immunopurified RNA fragments are dephosphorylated on the beads using T4 polynucleotide kinase (PNK). L7 linker, a 3′ end adapter, is ligated on the beads to the RNA component of the 90% purified RBP–RNA complexes, whereas pCp-IR750 is independently ligated to the RNA component of the remaining 10%. L7 linker contains unique molecular identifiers (UMIs) in a form of three random nucleotides, hereby named 3′UMIs. 3′UMIs are used to reduce sequence biases during L7 linker ligation. After extensive and stringent washes, two reactions are combined and RBP–RNA complexes are eluted from the beads. RBP–RNA complexes are resolved based on their size on the SDS-PAGE and transferred onto the nitrocellulose membrane. Complexes containing pCp-IR750 are visualized on the membrane in the Cy7 or IRDye 800CW channels. The membrane area corresponding to the RBP–RNA complexes is cut out and RNA released from the membrane using Proteinase K. RNA is further purified using silica columns. Note that the RNA contains a short peptide, a remnant of the UV-crosslinked RBP. The RNA is reverse transcribed into cDNA, during which reverse transcriptase stalls at the peptide–RNA crosslink site, generating truncated cDNAs. The terminal nucleotide at the 3′ end of the cDNA thus marks the RBP crosslink position. RNA is subsequently hydrolyzed, and cDNA is extracted using MyOne Silane beads. A second DNA adapter is then ligated to the cDNA 3′ ends; this adapter contains nine random nucleotides, hereby named 5′UMIs. cDNA molecules containing both adapters are purified using MyOne Silane beads and subjected to pre-amplification using short P5 and P7 primers. The resulting double-stranded DNA is size-selected with ProNex beads to remove adapter dimers. Finally, pre-amplified cDNA is further amplified using long P5 and P7 primers containing dual *i5* and *i7* indices for sample multiplexing. Final iCLIP3 libraries are purified with ProNex beads to remove unused primers, pooled in equimolar ratios, and sequenced on Illumina platforms.

**Figure 2:**
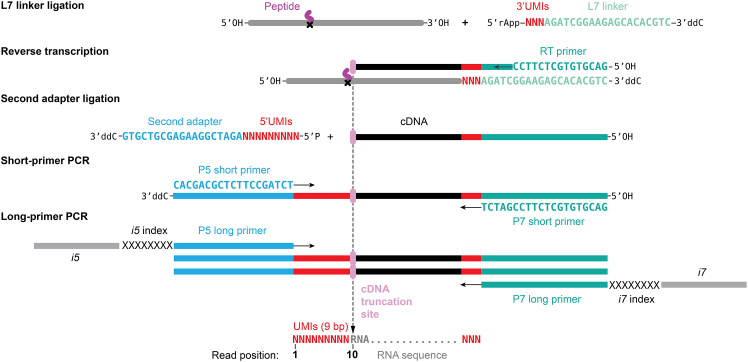
iCLIP3 oligonucleotide design L7 linker ligation: The L7 linker is a 3′ DNA adapter with a pre-adenylated 5′ end (5′-rApp), which enables efficient ligation to the 3′ end of immunopurified RNA in the absence of ATP. A dideoxycitidine modification at the 3′ end (3′-ddC) prevents self-ligation of the linker. The L7 linker also contains three random nucleotides (NNN) at its 5′ end to reduce ligation biases. These random nucleotides are referred to as 3′ unique molecular identifiers (3′UMIs) that are appended to the 3′ end of the RNA. Reverse transcription: Reverse transcription (RT) is performed using an RT primer complementary to the 3′ end of the L7 linker, generating cDNA from the RNA template. Second adapter ligation: The second DNA adapter is phosphorylated at its 5′ end to enable ligation to the 3′ ends of truncated cDNA molecules. A 3′-ddC modification prevents adapter self-ligation. The adapter contains nine random nucleotides (NNNNNNNNN) at its 5′ end (5′UMIs) which are used for deduplication of sequencing reads. **Short-primer PCR:** Short P5 and P7 primers (P5 short and P7 short) are used for cDNA pre-amplification. **Long-primer PCR:** Long P5 and P7 primers (P5 long and P7 long) are used for the final amplification of iCLIP3 libraries. These primers contain *i5* and *i7* sequences required for cluster generation on Illumina flow cells, as well as dual 8-nucleotide indices (XXXXXXXX) for sample multiplexing.

iCLIP3 builds upon the previously published iCLIP2 protocol^5^ and introduces several methodological improvements that simplify the workflow, enhance safety, and increase compatibility with standard sequencing pipelines. First, iCLIP3 replaces radioactive 5′ end RNA labeling with 3′ end RNA labeling using the pCp-IR750 dye, enabling non-radioactive, near-infrared visualization of RBP–RNA complexes (Figure 1). Second, RNA purification in iCLIP3 is performed using silica column-based isolation instead of phenol–chloroform extraction, simplifying sample handling and eliminating the use of organic solvents (Figure 1). Third, iCLIP3 incorporates TruSeq adapter sequences and unique dual indexing, which permits multiplexing of iCLIP3 libraries with unrelated RNA-seq libraries and their simultaneous sequencing (Figures 1 and 2). Finally, we provide a dedicated bioinformatics workflow for iCLIP3 data analysis. This workflow utilizes a modified version of the racoon_clip pipeline^6^ to extract singe-nucleotide crosslinking events from raw sequencing data^7^ and the R/Bioconductor package BindingSiteFinder^8^ to define RBP binding sites across biological replicates.

### Protocol optimization

CLIP-based protocols typically require RBP-specific optimization because their performance depends on the model organism used,^9^ on the activities of endogenous nucleases and proteases,^10^ on the expression level of the target RBPs, their subcellular localization and accessibility,^11^^,^^12^ the quality of IP-grade antibodies,^13^ the presence of paralogous proteins^14^ and ultimately, the protein’s capacity to bind RNA *in vivo*.^15^ To maximize iCLIP3 library preparation efficiency, we highly recommend verifying that the selected antibody efficiently immunoprecipitates the target RBP using iCLIP3 lysis and wash buffers. Additionally, before generating iCLIP3 sequencing libraries, follow the step-by-step instructions in Methods S1 to test the RNA-binding capacity of the target RBP *in vivo*, verify the isolation of clean RBP-RNA complexes, and optimize RNA fragmentation conditions.

## Key resources table

**Table:** 

REAGENT or RESOURCE	SOURCE	IDENTIFIER
**Antibodies**		

Anti-U2AF^65^ antibody, Mouse monoclonal (clone MC3), 5 μg per IP	Sigma-Aldrich	Cat#U4758

**Chemicals, peptides, and recombinant proteins**		

DMEM, high glucose, GlutaMAX, pyruvate	Thermo Fisher Scientific	Cat#10569010
Fetal bovine serum, Value	Thermo Fisher Scientific	Cat#A5256701
Penicillin-Streptomycin (10,000 U/mL)	Thermo Fisher Scientific	Cat#15140122
UltraPure DNase/RNase-Free Distilled Water	Thermo Fisher Scientific	Cat#10977035
UltraPure 1 M Tris-HCl, pH 7.5	Thermo Fisher Scientific	Cat#15567027
NaCl (5M), RNase-free	Thermo Fisher Scientific	Cat#AM9759
Sodium deoxycholate	Sigma-Aldrich	Cat#D6750-100G
Igepal CA-630	Sigma-Aldrich	Cat# I8896-50ML
Tween 20	Sigma-Aldrich	Cat# P9416-50ML
UltraPure SDS solution, 10%	Thermo Fisher Scientific	Cat#15553027
MgCl_2_ (1M)	Thermo Fisher Scientific	Cat#AM9530G
EDTA (0.5 M), pH 8.0, RNase-free	Thermo Fisher Scientific	Cat#AM9260G
1M Tris-HCl, pH 6.5	Sigma-Aldrich	Cat#20-160
DL-Dithiothreitol solution (1 M in H_2_0)	Sigma-Aldrich	Cat#646563-10X.5ML
cOmplete, Mini, EDTA-free Protease Inhibitor Cocktail	Sigma-Aldrich	Cat#11836170001
Ambion RNase I, cloned, 100 U/μL	Thermo Fisher Scientific	Cat#AM2294
TURBO DNase (2 U/μL)	Thermo Fisher Scientific	Cat#AM2238
Recombinant RNasin Ribonuclease Inhibitor	Promega	Cat#N2511
T4 Polynucleotide kinase (T4 PNK)	NEB	Cat#M0201S
T4 RNA Ligase 1 (ssRNA Ligase), High Concentration	NEB	Cat#M0437M
10X T4 RNA Ligase Reaction buffer	NEB	Component of Cat#M0437M
50% Polyethylene Glycol (PEG) 8000	NEB	Component of Cat#M0437M
100 mM ATP	NEB	Component of Cat#M0437M
pCp-IR750 (1 mM)	Jena Bioscience	Cat#NU-1706-IR750
NuPAGE 4%–12% Bis-Tris Mini Protein Gels	Thermo Fisher Scientific	Cat#NP0322BOX
NuPAGE MOPS SDS Running Buffer (20X)	Thermo Fisher Scientific	Cat#NP0001
NuPAGE Transfer Buffer (20X)	Thermo Fisher Scientific	Cat#NP00061
NuPAGE LDS Sample Buffer (4X)	Thermo Fisher Scientific	Cat#NP0007
PageRuler Prestained Protein Ladder	Thermo Fisher Scientific	Cat#26616
Proteinase K, recombinant, PCR Grade (20 mg/mL)	Sigma-Aldrich	Cat#3115828001
Phenylmethylsulfonyl Fluoride (PMSF)	Cell Signaling Technology	Cat#8553S
Methanol	Carl Roth	Cat#0082.1
Ethanol	Sigma-Aldrich	Cat#32205-1L-M
2-Propanol (Isopropanol)	Sigma-Aldrich	Cat#190764
SuperScript III Reverse Transcriptase	Thermo Fisher Scientific	Cat#18080044
5X First Strand Buffer	Thermo Fisher Scientific	Component of Cat#18080044
0.1 M DTT	Thermo Fisher Scientific	Component of Cat#18080044
Deoxynucleotide (dNTP) Solution mix, 10 mM each	NEB	Cat#N0447S
HEPES Buffer Solution (1 M)	Sigma-Aldrich	Cat#H0887-20ML
Sodium Hydroxide, pellet	Sigma-Aldrich	Cat#S8045-500G
Buffer RLT	QIAGEN	Cat#79216
Phusion High-Fidelity PCR Master Mix with HF Buffer	NEB	Cat#M0531S
DMSO	NEB	Component of Cat#M0531S
GeneRuler Ultra Low Range DNA ladder	Thermo Fisher Scientific	Cat#SM1211

**Critical commercial assays**		

Dynabeads Protein G for Immunoprecipitation	Thermo Fisher Scientific	Cat#10003D
RNA Clean & Concentrator-5 Kit	Zymo Research	Cat#R1015
Dynabeads MyOne Silane	Thermo Fisher Scientific	Cat#37002D
ProNex Size-Selective Purification System	Promega	Cat#NG2001
High Sensitivity D1000 ScreenTape	Agilent	Cat#5067-5584
High Sensitivity D1000 Reagents	Agilent	Cat#5067-5585
Qubit 1X dsDNA High Sensitivity (HS) Assay Kit	Thermo Fisher Scientific	Cat#Q33230

**Deposited data**		

Genome assembly (e.g., GRCm38/hg38)	GENCODE	https://www.gencodegenes.org
Gene annotation (e.g., GENCODE, release 49)	GENCODE	https://www.gencodegenes.org
(optional) rRNA sequences	NCBI/Refseq	https://www.ncbi.nlm.nih.gov/nuccore/
(optional) Genomes of potential contaminating organisms	ENSEMBL	https://www.ensembl.org/index.html
U2AF2 iCLIP3	This study	GEO: GSE325775
U2AF2 iCLIP2 rep1	Ebersberger et al.^16^	GEO: GSM6793346
U2AF2 iCLIP2 rep2	Ebersberger et al.^16^	GEO: GSM6793347

**Experimental models: Cell lines**		

Human HeLa cells	This study	N/A

**Oligonucleotides**		

L7 linker:/5rApp/NNNAGATCGGAAGAG CACACGTC/3ddC/	Integrated DNA Technologies	N/A
RT primer: GACGTGTGCTCTTCC	Integrated DNA Technologies	N/A
Second adapter:/5Phos/NNNNNNNNNAGAT CGGAAGAGCGTCGTG/3ddC/	Integrated DNA Technologies	N/A
P5 short (P5S): ACACGACGCTCTTCCGATC∗T	Integrated DNA Technologies	N/A
P7 short (P7S): GACGTGTGCTCTTCCGATC∗T	Integrated DNA Technologies	N/A
P5 long (P5L): AATGATACGGCGACCACCGA GATCTACAC[8 nt index]ACACTCTTTCCCTA CACGACGCTCTTCCGATC∗T	Integrated DNA Technologies	N/A
P7 long (P7L): CAAGCAGAAGACGGCATAC GAGAT[8 nt index]GTGACTGGAGTTCAGA CGTGTGCTCTTCCGATC∗T	Integrated DNA Technologies	N/A

**Software and algorithms**		

DNA Barcode Combination Finder Shiny app	This study	https://www.biozentrum.uni-wuerzburg.de/brb/resources/
racoon_clip	Klostermann & Zarnack {Klostermann, 2024, racoon_clip-a complete pipeline for single-nucleotide analyses of iCLIP and eCLIP data}	https://github.com/ZarnackGroup/racoon_clip
Conda or Docker or Apptainer	Anaconda/Docker/Apptainer	https://anaconda.org/, https://www.docker.com/, https://apptainer.org/

**Other**		

BioLite Cell Culture Treated Dishes, 100 mm	Thermo Fisher Scientific	Cat#130182
BIO-LINK BLX-254 UV Crosslinker	Vilber	N/A
Branson digital sonifier 250 with 1/8″ tip	Emerson	N/A
DynaMag-2 Magnet magnetic rack (1.5 ml tubes)	Thermo Fisher Scientific	Cat#12321D
Magnetic Separation Rack, 0.2 mL tubes	EpiCypher	Cat#10-0008-EPC
Proteus Clarification Mini Spin Columns	SERVA Electrophoresis	Cat#42225.01
XCell SureLock Mino-Cell and XCell II Blot Module	Thermo Fisher Scientific	Cat#EI0002
Whatman 3MM Chromatography Paper	GE Healthcare	Cat#3030917
Amersham Protran 0.45 μm Nitrocellulose Membrane	Cytiva	Cat#10600002
Swann-Morton Stainless Surgical Scalpels	Fisher Scientific	Cat#11728353
PowerPac Basic Power Supply	Bio-Rad	Cat#1645050
ChemiDoc MP Imaging System	Bio-Rad	N/A
Thermomixer	N/A	N/A
Falcon 15 mL Conical Centrifuge Tubes	Corning	Cat#CLS352096
Multiply μStripPro 0.2 mL PCR 8-Tube Strips	SARSTEDT	Cat#72.991.002
SafeSeal Reaction Tube, 1.5 mL, DNA Low Binding	SARSTEDT	Cat#72.706.700
Nonstick, RNase-free Microfuge Tubes, 1.5 mL	Thermo Fisher Scientific	Cat#AM12450
ROTILABO PES Syringe Filters, 0.22 μm	Carl Roth	Cat#P668.1
Omnifix 50 mL Disposable Syringes	Carl Roth	Cat#T552.2
Omnifix 10 mL Disposable Syringes	Carl Roth	Cat#C542.1
PCR Themocycler	N/A	N/A
Qubit Flex Fluorimeter	Thermo Fisher Scientific	N/A
Tape Station 4150 or 4200	Agilent	N/A
Tape Station Optical Tube Strips	Agilent	Cat#401428
Tape Station Optical Tube Strip Caps	Agilent	Cat#401425

## Materials and equipment

Prepare all buffers in nuclease-free water

**Table:** Lysis buffer

Reagent	Final concentration	Volume (mL)
1 M Tris-HCl, pH 7.5	50 mM	2
5 M NaCl	100 mM	0.8
10% Igepal CA-630 (v/v)	1%	4
5% Na-deoxycholate (w/v)	0.5%	4
10% SDS	0.1%	0.4
Nuclease-free water	N/A	28.8
Total	N/A	**40**

Filter the buffer through 0.22 μm filter and store at 4°C for up to 1 month.

**Table:** High Salt buffer

Reagent	Final concentration	Volume (mL)
1 M Tris-HCl, pH 7.5	50 mM	2
5 M NaCl	1 M	8
10% Igepal CA-630 (v/v)	1%	4
5% Na-deoxycholate (w/v)	0.5%	4
10% SDS	0.1%	0.4
0.5 M EDTA, pH 8.0	1 mM	0.08
Nuclease-free water	N/A	21.52
Total	N/A	**40**

Filter the buffer through 0.22 μm filter and store at 4°C for up to 1 month.

**Table:** PNK Wash buffer

Reagent	Final concentration	Volume (mL)
1 M Tris-HCl, pH 7.5	20 mM	0.8
1 M MgCl_2_	10 mM	0.4
10% Tween-20 (v/v)	0.2%	0.8
Nuclease-free water	N/A	38
Total	N/A	**40**

Filter the buffer through 0.22 μm filter and store at 4°C for up to 1 month.

**Table:** Last Wash buffer

Reagent	Final concentration	Volume (mL)
1 M Tris-HCl, pH 7.5	20 mM	0.4
5 M NaCl	100 mM	0.4
10% Tween-20 (v/v)	0.2%	0.4
0.5 M EDTA, pH 8.0	2 mM	0.08
Nuclease-free water	N/A	18.72
Total	N/A	**20**

Filter the buffer through 0.22 μm filter and store at 4°C for up to 1 month.

**Table:** 5X PNK buffer pH 6.5

Reagent	Final concentration	Volume (μL)
1 M Tris-HCl, pH 6.5	350 mM	70
250 mM MgCl_2_	50 mM	40
100 mM DTT	5 mM	10
Nuclease-free water	N/A	80
Total	N/A	**200**

Store aliquots of the buffer at −20°C. Do not thaw and freeze again.

**Table:** Proteinase K buffer

Reagent	Final concentration	Volume (mL)
1 M Tris-HCl, pH 7.5	100 mM	1
5 M NaCl	50 mM	0.1
0.5 M EDTA pH 8.0	10 mM	0.2
10% SDS (v/v)	0.2%	0.2
Nuclease-free water	N/A	8.5
Total	N/A	**10**

Filter the buffer through 0.22 μm filter and store at 4°C for up to 1 month.

**Table:** PEG/NaCl Mix

Reagent	Final concentration	Volume (μL)
50% PEG 8000	20%	200
5 M NaCl	2.5 M	250
Nuclease-free water	N/A	50
Total	N/A	**500**

Store the solution in a parafilm sealed 1.5 mL tube at 4°C for up to 3 months.

## Step-by-step method details

### Cell culture and UV irradiation

**Timing: Variable, 2 days for sample preparation**

This section describes how to crosslink RBPs to their RNA targets by briefly exposing cells to 254 nm UV light. In addition to the primary UV-irradiated (UV+) sample with RBP-specific IP, we recommend including a non-irradiated (UV–) control with RBP-specific IP and a UV+ control sample with non-immune IgG or beads-only IP.

1. Seed cells for the experiment.
  a. Grow HeLa cells in high glucose, pyruvate 1X DMEM + GlutaMAX medium supplemented with 10% heat-inactivated FBS and 1X Penicillin-Streptomycin (Pen-Strep).
  b. One day before the experiment, seed HeLa cells in a 100 mm dish to achieve ∼85% confluency the following day.
2. Wash cells with PBS.
  a. Aspirate the culture medium from the dishes.
  b. Gently wash the cells with 6 mL ice-cold PBS.
  c. Remove the PBS and add 6 mL fresh ice-cold PBS to the dishes.

***Note:*** Consider adding protease inhibitors to PBS if the cell line of interest is known to show significant protease activity during cell harvesting.

3. Irradiate cells with 254 nm UV light.
  a. Place the cell dishes containing PBS on an ice tray covered with a thin layer of water (Figure S1).
  b. Remove the lids and irradiate the cells once with 254 nm UV light at 150 mJ/cm^2^.
4. Harvest cells.
  a. Gently scrape the cells in PBS using cell lifters.
  b. Transfer the cell suspensions into 15 mL Falcon tubes labeled with sample names.
  c. Pellet the cells by centrifugation at 300 x *g* for 5 min at 4°C.
    ***Note:*** If available, use a swinging-bucket rotor to ensure the cell pellets concentrate compactly at the bottom of the tubes.
  d. Carefully aspirate the PBS without disturbing the cell pellets.
  e. Snap-freeze the cell pellets on dry ice or in liquid nitrogen and store at −80°C.
    **Pause point:** Frozen cell pellets can be stored at −80°C long term until further use.

### Antibody-bead coupling

#### Day 1

**Timing:15 min +1–2 h**

This section describes how to couple the antibody for the target RBP to magnetic beads.

5. Couple the antibody to magnetic Protein A or Protein G Dynabeads.
  ***Note:*** Verify the antibody’s binding preference for Protein A or Protein G Dynabeads by consulting the manufacturer’s recommendations.
  a. Aliquot 30 μL Protein A or Protein G Dynabeads per sample into a 1.5 mL tube.
    ***Note:*** Adjust the Dynabeads aliquot volume according to the number of samples.
  b. Wash the beads twice with 750 μL Lysis buffer.
  c. Resuspend the beads in 300 μL Lysis buffer and add 5 μg antibody per sample for the RBP of interest.
    ***Note:*** Adjust the amount of antibody based on the number of samples. Use 500 μL Lysis buffer for ≥ 6 samples. All washes in the protocol are performed by pipetting unless otherwise stated.
  d. Incubate the beads on a rotating wheel at 20°C–24°C for ≥ 1 h (until the lysates are ready for immunoprecipitation).

### Cell lysis and protein quantification

#### Day 1

**Timing: 1 h**

This section describes how to lyse frozen cell pellets and quantify the total protein concentration in the resulting lysates.

6. Lyse cell pellets.
  a. Thaw the frozen cell pellets on ice.
  b. Resuspend the cell pellets in 750 μL Lysis buffer supplemented with 1X PIs.
  c. Lyse the cells on ice for 10 min.
    ***Optional:*** Sonicate the lysates on ice with Branson digital sonifier 250 at 10% power amplitude using 5 cycles of 5 s pulses with 10 s pauses between pulses.
    ***Note:*** Sonication is recommended for nuclear proteins. If sonication is omitted, extend the lysis time to 20 min on ice. The sonication settings described here may not be fully applicable to another type of sonifier and may require optimization.
  d. Transfer the lysates to new 1.5 mL tubes.
  e. Clarify the lysates by centrifugation at 16,000 x *g* for 10 min at 4°C.
  f. Transfer the supernatants to fresh 1.5 mL tubes.
7. Determine the protein concentration of the cleared lysates using a BCA Protein Assay kit according to the manufacturer’s instructions.
8. Prepare lysates for RNA fragmentation.
  a. Dilute the lysates to a final protein concentration of 0.5 mg/mL using Lysis buffer supplemented with 1X PIs.
  b. Transfer 500 μL of each diluted lysate (corresponding to 250 μg of total protein) into a new 1.5 mL tube and keep the samples on ice.

### RNA fragmentation

#### Day 1

**Timing: 20 min**

This section describes how to perform partial RNA digestion in the lysate using RNase I to generate fragments within a 50 – 300 nt size range suitable for iCLIP3 library generation and sequencing.

9. Treat lysates with DNase.
  a. Add 2 μL **TURBO DNase** to the diluted lysates from Step 8b.
  b. Gently invert the tubes several times to mix.
  c. Briefly spin down the samples and place on ice.
10. Fragment RNA in lysates with RNase I.
  a. Prepare a medium **RNase I concentration** in nuclease-free water as follows:

**Table:** 

RNase I	RNase I, 100 U/μL (μL)	Nuclease-free water (μL)
Medium (M, 0.67 U/μL)	1	149
    **CRITICAL:** Thoroughly pipette the prepared RNase I dilution before proceeding. Do not change the RNase I concentration (0.67 U/μL, 1:150 dilution) for HeLa and P19 cell lysates containing 250 μg of total protein at a protein concentration of 0.5 mg/mL. Consult Methods S1 for detailed instructions on how to evaluate the size of the RNA fragments derived from the recommended RNase I concentration.
  b. Add 10 μL medium **RNase I concentration** to the lysates.
  c. Gently invert the tubes several times to mix, then briefly spin down.
  d. Immediately incubate the samples in a thermomixer at 37°C for 3 min with shaking at 1,100 rpm.
    **CRITICAL:** Strictly adhere to a 3 min-long incubation for consistent results.
  e. Immediately place the samples on ice after incubation and keep them for ≥ 3 min to stop the RNA digestion.
    ***Optional:*** Load the samples onto Proteus mini clarification spin columns, centrifuge at 16,000 x *g* for 1 min at 4°C and transfer the flow-throughs to new 1.5 mL tubes.

### Immunoprecipitation

#### Day 1

**Timing: 2.5 h**

This section describes how to immunopurify the target RBP from RNase I-treated lysates using magnetic beads coupled with an RBP-specific antibody.

11. Clean up antibody-bead complexes.
  a. Briefly spin down the antibody-bead coupling mixture.
  b. Place the tubes on a magnetic rack to collect the beads and discard the supernatant.
  c. Wash the beads twice with 750 μL Lysis buffer.
  d. Resuspend the beads for each sample in 100 μL Lysis buffer supplemented with 1X PIs.
12. Immunoprecipitate target RBP.
  a. Add 100 μL antibody-coupled beads from Step 11d to the RNase I-treated lysates from Step 10e.
  b. Incubate the lysates with the beads on a rotating wheel for 2 h at 4°C.

***Note:*** Adjust the incubation time according to the optimized immunoprecipitation conditions for the RBP of interest.

13. Clean up immunoprecipitation.
  a. Briefly spin down the samples and place them on a magnetic rack to collect the beads.
  b. Wash the beads twice with 800 μL High Salt buffer. Incubate the second wash on a rotating wheel for 5 min at 4°C. After incubation, briefly spin down the samples to collect beads from the tube lid before proceeding.
  c. Wash the beads twice with 800 μL Lysis buffer. During the second Lysis buffer wash, transfer the beads to a new 1.5 mL tube.
  d. Wash the beads once with 800 μL PNK Wash buffer.
  e. Perform a final wash with 300 μL PNK Wash buffer.
  f. Keep the beads on ice until proceeding to the next step.

**Pause point:** The beads can be kept in the PNK Wash buffer on ice for 1–2 h.

### 3′ RNA dephosphorylation

#### Day 1

**Timing: 45 min**

RNase I generates RNA fragments with 2′,3′-cyclic phosphates and 3′-phosphates, which require conversion to 3′-hydroxyl groups to enable efficient ligation of pCp-IR750 and DNA adapters. This section describes how to dephosphorylate crosslinked RNA fragments on beads using T4 polynucleotide kinase (PNK).

14. Dephosphorylate RNA 3′ ends.
  a. Prepare the RNA Dephosphorylation buffer, mix thoroughly by pipetting and keep on ice.

**Table:** 

Reagent	Volume per reaction (μL)
Nuclease-free water	14.5
5X T4 PNK buffer (pH 6.5)	4
RNasin	0.5
Total	**19**
  b. Place the samples from Step 13f on a magnetic rack to collect the beads and remove the PNK Wash buffer.
  c. Briefly spin down the tubes, return them quickly to the magnetic rack, and remove any residual buffer using a P20 pipette.
    ***Note:*** Process one sample at a time to avoid drying the beads.
  d. Resuspend the beads in 19 μL RNA Dephosphorylation buffer.
  e. Add 1 μL T4 PNK and mix thoroughly by pipetting.
  f. Incubate the samples for 20 min at 37°C in a thermomixer with shaking at 1,200 rpm.
    ***Note:*** Place the samples in the thermomixer immediately after assembling the reaction to prevent bead sedimentation.
15. Clean up 3′ RNA dephosphorylation.
  a. Add 100 μL PNK Wash buffer without resuspending the beads.
  b. Place the samples on a magnetic rack to collect the beads and remove the PNK Wash buffer.
  c. Wash the beads twice with 800 μL High Salt buffer. Incubate the second wash for 2 min on a rotating wheel at 4°C. After incubation, briefly spin down the samples to collect beads from the tube lid before proceeding.
  d. Wash the beads with 800 μL Lysis buffer. Transfer the beads to a new 1.5 mL tube during this wash.
  e. Wash the beads once with 800 μL PNK Wash buffer.
  f. Perform a final wash with 300 μL PNK Wash buffer.
  g. Thoroughly resuspend the beads by pipetting and divide each sample into two 1.5 mL tubes as follows:
    i. Tube 1: 270 μL (90% beads)
    ii. Tube 2: 30 μL (10% beads)
  h. Store Tube 2 (10% beads) at 4°C for ≤20 h and keep Tube 1 (90% beads) on ice.

### 3′ L7 linker ligation

#### Days 1–2

**Timing: 1 h**

This section describes how to ligate the 5′-preadenylated L7 linker (a DNA adapter) on beads to 90% of the immunopurified RNA fragments with T4 PNK-repaired ends. Ligation of the L7 linker is essential for converting RBP-bound RNA into iCLIP3 sequencing libraries.

16. Ligate L7 linker to RNA 3′ ends.
  a. Prepare the RNA Ligation buffer, mix well by pipetting and keep it at 20°C–24°C:

**Table:** 

Reagent	Volume per reaction (μL)
Nuclease-free water	8.75
10X RNA Ligase Reaction buffer	2
20 μM L7 adapter	1.5
RNasin	0.25
50% PEG8000	6
Total	**18.5**
    ***Note:*** Pipette the assembled RNA Ligation buffer slowly and thoroughly to ensure homogeneous distribution of all components. PEG8000 is highly viscous; avoid liquid retention in the pipette tip.
  b. Place each Tube 1 from Step 15h on a magnetic rack to collect the beads and remove the PNK Wash buffer.
  c. Briefly spin down the tubes, return them quickly to the magnetic rack, and remove any residual buffer using a P20 pipette.
    ***Note:*** Process one sample at a time to avoid drying the beads.
  d. Resuspend the beads in 18.5 μL RNA Ligation buffer.
  e. Add 1.5 μL T4 RNA Ligase 1 (high concentration).
  f. Mix the reactions thoroughly by pipetting until the beads are evenly distributed. Pipette slowly to avoid bead retention in the pipette tip.
  g. Incubate the ligation reactions at 16°C for 16 h–20 h in a thermomixer with shaking at 1,200 rpm.
17. Clean up 3′ L7 linker ligation (Day 2).
  a. Add 100 μL PNK Wash buffer without resuspending the beads.
  b. Place the samples on a magnetic rack to collect the beads and remove the PNK Wash buffer.
  c. Wash the beads twice with 800 μL High Salt buffer. Incubate the second wash for 2 min on a rotating wheel at 4°C. After incubation, briefly spin down the samples to collect beads from the tube lid before proceeding.
  d. Wash the beads with 800 μL Lysis buffer. Transfer the beads to a new 1.5 mL tube during this wash.
  e. Wash the beads twice with 800 μL PNK Wash buffer.
  f. Leave the beads on ice in the PNK Wash buffer.

### 3′ RNA labeling with pCp-IR750

#### Day 2

**Timing: 1.5 h**

**Figure 3:**
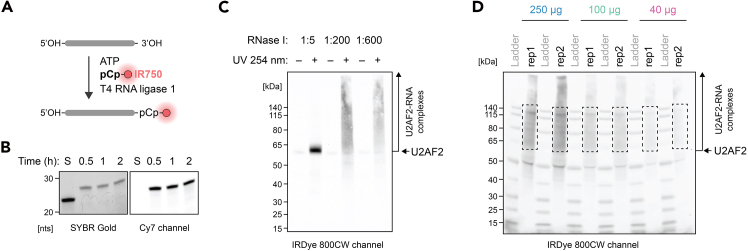
pCp-IR750 RNA labeling enables rapid and safe visualization of RNA–protein complexes (A) Infrared 3′ end RNA labeling requires pCp-IR750, ATP, and T4 RNA ligase 1. (B) Efficiency of infrared 3′ end RNA labeling with pCp-IR750. A 15% TBE–urea gel shows attachment of pCp-IR750 to a 24-nucleotide (nt) RNA oligonucleotide. RNA was labeled with pCp-IR750 for 0.5, 1, and 2 h at 27°C. Lane S contains unlabeled (input) RNA. The gel was stained with SYBR Gold and imaged in both SYBR Gold and Cy7 channels. (C) iCLIP3-based visualization of immunopurified protein–RNA complexes. A nitrocellulose membrane shows pCp-IR750-labeled RNA associated with immunopurified U2AF2–RNA complexes from UV-irradiated and non-irradiated samples treated with different RNase I concentrations (high, 20 U/μL, 1:5 dilution; medium, 0.5 U/μL, 1:200 dilution; low, 0.167 U/μL, 1:600 dilution). Only 10% of the immunoprecipitation (IP) material was subjected to pCp-IR750 RNA labeling. (D) Nitrocellulose membrane showing U2AF2–RNA complexes immunopurified from HeLa cell lysates containing 250 μg, 100 μg and 40 μg total protein. Only 10% of the immunopurified U2AF2–RNA complexes in each sample and each replicate were labeled with pCp-IR750. The remaining 90% of the U2AF2–RNA complexes were subjected to L7 linker ligation. Both 10% and 90% fractions were combined and ran in the same gel lane. U2AF2-RNA complexes were visualized in (C–D) in the IRDye 800CW channel. Note that the 40 μg and 100 μg samples were derived by diluting the RNase I-digested 250 μg sample in Lysis buffer containing 1X PIs. Medium RNase I concentration, 0.67 U/μL (1:150 dilution) was used.

This section describes how to ligate pCp-IR750 on beads to 10% of the immunopurified RNA fragments with T4 PNK-repaired 3′ ends. This labeling step enables rapid and safe visualization of RBP-RNA complexes on a nitrocellulose membrane (Figures 3A and 3B).

***Note:*** pCp-IR750 requires imaging using near-infrared Cy7 or IRDye 800CW channels. We do not recommend pCp-Cy5 due to high background signal on the membrane in the Cy5 imaging channel. We did not thoroughly evaluate other pCp-fluorophore conjugates.

18. Label RNA 3′ ends with pCp-IR750.
  a. Prepare the RNA Labeling buffer, mix thoroughly by pipetting and keep at 20°C–24°C.

**Table:** 

Reagent	Volume per reaction (μL)
Nuclease-free water	7.75
10X RNA Ligase Reaction buffer	2
10 mM ATP	2
200 μM pCp-IR750	1
RNasin	0.25
50% PEG8000	6
**Total**	**19**
    ***Note:*** Pipette the assembled RNA Labeling buffer slowly and thoroughly to ensure homogeneous distribution of all components. PEG8000 is highly viscous; avoid liquid retention in the pipette tip. Protect the buffer from light.
  b. Place each Tube 2 (10% beads) from Step 15h on a magnetic rack to collect the beads and remove the PNK Wash buffer.
  c. Resuspend the beads in 19 μL RNA Labeling buffer.
  d. Add 1 μL T4 RNA Ligase 1 (high concentration).
  e. Mix the reactions thoroughly by pipetting until the beads are evenly distributed. Pipette slowly to avoid bead retention in the pipette tip.
  f. Incubate the reactions at 27°C for 1 h in a thermomixer with shaking at 1,200 rpm.
    **CRITICAL:** Do not incubate the labeling reaction longer than 1 h to avoid protein labeling. Protect the samples from light using aluminum foil.
19. Clean up 3′ RNA labeling reaction.
  a. Add 100 μL PNK Wash buffer without resuspending the beads.
  b. Place the samples on a magnetic rack to collect the beads and remove the PNK Wash buffer.
  c. Wash the beads twice with 400 μL High Salt buffer.
  d. Wash the beads with 400 μL Lysis buffer. Transfer the beads to a new 1.5 mL tube during this wash.
  e. Wash the beads once with 400 μL PNK Wash buffer.
  f. Perform a final wash with 100 μL PNK Wash buffer.

### Elution of RBP-RNA complexes

#### Day 2

**Timing: 20 min**

This section describes how to combine, wash, and elute the L7 linker-ligated and pCp-IR750-labeled RBP-RNA complexes from the beads. While pCp-IR750 RNA labeling allows visualization of the immunopurified RBP–RNA complexes, L7 linker ligation enables conversion of the RNA into iCLIP3 sequencing libraries.

20. Combine beads from Tube 1 and Tube 2.
  a. For each sample, transfer the beads from Tube 2 (100 μL) from Step 19f into Tube1 (800 μL) from Step 17f.
  b. Wash the combined beads with 300 μL Last Wash buffer.
21. Elute RBP-RNA complexes.
  a. Collect the beads on a magnetic rack and discard the Last Wash buffer.
  b. Resuspend the beads in 20 μL 1X LDS NuPAGE Loading buffer supplemented with 50 mM DTT.
  c. Incubate the beads in a thermomixer at 70°C for 5 min with shaking at 1,100 rpm.
  d. Briefly spin down the samples, collect the beads on a magnetic rack, and transfer the eluates to new 1.5 mL tubes.

### SDS-PAGE and nitrocellulose transfer of RBP-RNA complexes

#### Day 2

**Timing: 3 h**

This section describes how to resolve the eluted RBP-RNA complexes using denaturing SDS-PAGE and how to transfer them onto a nitrocellulose membrane. Because proteins efficiently bind nitrocellulose, directly crosslinked RNA remains attached to the membrane via the RBP, whereas background or non-crosslinked RNA does not stably bind.

22. Resolve RBP-RNA complexes using SDS-PAGE.
  a. Prepare 0.5 L 1X NuPAGE MOPS SDS Running buffer using nuclease-free water.
  b. Prepare the Prestained Protein Ruler (protein ladder) dilution for two gel lanes per sample by mixing 6.25 μL protein ladder stock solution with 43.75 μL 1X LDS NuPAGE Loading buffer (includes 2.5x surplus).
  c. Assemble a 12-well 4–12% Bis-Tris SDS gel in the XCell SureLock module according to the manufacturer’s instructions.
  d. Load 20 μL protein ladder dilution in two gel lanes, leaving an empty gel lane between them.
  e. Load 20 μL sample from Step 21d in the empty lane between the protein ladder lanes.
  f. Run the gel at 180 V for 50–60 min.
23. Transfer RBP–RNA complexes to a nitrocellulose membrane.
  a. Prepare 1X NuPAGE Transfer buffer with 20X buffer stock using nuclease-free water and 20% (vol/vol) methanol or ethanol.
  b. Cut the nitrocellulose membrane and four pieces of Whatman paper.
  c. Carefully open the gel cassette.
  d. Assemble the transfer sandwich using the XCell II module according to the manufacturer’s instructions.
    ***Note:*** Pre-wet the sponges and remove any air bubbles using a roller.
  e. Perform the transfer in 1X NuPAGE Transfer buffer for 1.5 h at 30 V.
    ***Note:*** For proteins smaller than 50 kDa, transfer for 1 h at 30 V. For large proteins (>180 kDa), longer transfer times may be required.

### Membrane imaging

#### Day 2

**Timing: 15 min**

This section describes how to directly visualize the pCp-IR750-labeled, immunopurified RBP-RNA complexes on the nitrocellulose membrane.

24. Visualize immunopurified RBP–RNA complexes.
  a. Disassemble the transfer sandwich.
  b. Using forceps, place the membrane in a clean plastic tray containing 1X PBS.
  c. Place the membrane onto a thin, transparent plastic foil and insert it into the imager.
    ***Note:*** Do not place the membrane directly onto the transilluminator plate to avoid contamination from previously imaged nucleic acids.
  d. Image the membrane using the Cy7 or IRDye 800CW channel to visualize the RBP–RNA complexes.
  e. Image the membrane using the colorimetric channel to visualize the protein ladder.
  f. Merge the two images into a single composite image and save.
  g. Return the membrane to the plastic tray containing 1X PBS.
25. Inspect the image and identify the region of the membrane showing RNA signal that corresponds to the size range of immunopurified RBP–RNA complexes (Figures 3C and 3D; Figures S2A and S2B).

***Note:***PageRuler prestained protein ladder bands exhibit different apparent molecular weights when resolved on a Bis-Tris gel using MOPS SDS running buffer compared to their nominal sizes.

### Elution of RNA from the membrane with Proteinase K treatment

#### Day 2

**Timing: 1 h**

This section describes how to excise nitrocellulose regions containing RBP-RNA complexes and treat them with Proteinase K. As a non-specific protease, Proteinase K digests the RBP to release both pCp-IR750-labeled (10%) and L7 linker-ligated (90%) RNA from the membrane.

26. Cut out the membrane part containing RBP-RNA complexes.
  a. Place the membrane on a transparent, thick foil.
  b. Using sterile scalpels, cut the sample lane between two proteins ladders corresponding to the size of the immunopurified RBP–RNA complexes (Figure 3D; Figure S2B).
  c. Transfer the excised membrane region to a clean 6-cm or 10-cm dish.
  d. Shred the membrane further into smaller pieces.
  e. Prepare a 1.5 mL tube containing 150 μL Proteinase K buffer.
  f. Using needle tips, transfer the membrane pieces into the 1.5 mL tube containing the Proteinase K buffer.

***Note:*** Ensure that all membrane pieces are fully submerged in Proteinase K buffer.

27. Digest RBP with Proteinase K.
  a. Add 10 μL Proteinase K (20 mg/mL) to each sample.
  b. Incubate the samples in a thermomixer at 37°C for 20 min with shaking at 1,000 rpm.
  c. Continue the incubation in a thermomixer at 50°C for 20 min with shaking at 1,000 rpm.
  d. Briefly spin down the samples and transfer the supernatants (∼155 μL) to new 1.5 mL tubes.

### RNA isolation

#### Day 2

**Timing: 15 min**

This section describes how to purify the RNA released after Proteinase K treatment using a silica column-based approach.

28. Inactivate Proteinase K.
  a. Add 45 μL nuclease-free water to the samples from Step 27d.
  b. Add 1 μL 0.5 M PMSF and briefly vortex.
  c. Briefly spin down the samples and incubate at 20°C–24°C for ≥ 3 min.
29. Isolate RNA using RNA Clean and Concentrator-5 kit.
  a. Add 400 μL (2 volumes) RNA Binding buffer and briefly vortex.
  b. Briefly spin the samples, add 700 μL 100% isopropanol (3.5X starting volume) and briefly vortex.
  c. Incubate the samples for 15 min at 20°C–24°C on a rotating wheel.
  d. Briefly spin down the samples, transfer 650 μL into the Zymo-Spin IC column and centrifuge at 5,000 x g for 30 s at 20°C–24°C.
  e. Discard the flow-through from each collection tube and add the remaining sample to the same column.
  f. Centrifuge at 5,000 x g for 30 s at 20°C–24°C and discard the flow-throughs.
  g. Wash the columns with 400 μL RNA Prep buffer, centrifuge at 5,000 x g for 30 s at 20°C–24°C and discard the flow-throughs.
  h. Wash the columns with 500 μL RNA Wash buffer (with ethanol added), centrifuge at 5,000 x g for 30 s at 20°C–24°C and discard the flow-throughs.
  i. Wash the columns with 250 μL RNA Wash buffer (with ethanol added), centrifuge at 9,000 x g for 30 s at 20°C–24°C and discard the flow-throughs.
  j. Centrifuge at 9,000 x g for additional 2 min at 20°C–24°C and transfer the columns to clean 1.5 mL tubes, being careful to avoid contact between the wash buffer and the column.
  k. Add 10.3 μL nuclease-free water to the columns, incubate at 37°C for 2 min and then centrifuge at 15,000 x g for 1 min at 20°C–24°C.
  l. Discard the columns and store the eluted RNA at −80°C.

**Pause point:** RNA can be stored at −80°C until the next day.

### Reverse transcription

#### Day 3

**Timing: 1.5 h**

This section describes how to convert eluted RNA into cDNA using reverse transcriptase. The RT primer anneals to the 3′ end of the L7 linker, and reverse transcriptase synthesizes a cDNA copy. Residual peptide left after Proteinase K digestion at the crosslinked nucleotide causes premature termination of reverse transcription and generates truncated cDNA.

30. Perform cDNA synthesis.
  a. Thaw the isolated RNA from Step 29l on ice and transfer 10 μL to a PCR tube.
  b. Prepare the following master mix:

**Table:** 

Reagent	Volume per reaction (μL)
dNTP mix (10 mM each)	1
iCLIP3 RT primer (2 μM)	1
Total	**2**
  c. Add 2 μL master mix to 10 μL isolated RNA and mix well.
  d. Incubate the mixture in a thermocycler with the lid heated to the maximum temperature (usually 105°C) using the following program:

**Table:** 

Steps	Temperature	Time	Cycle number
Denaturation	70°C	5 min	1
Hybridization	25°C	∞	1
  e. Prepare the following RT mix, pipette thoroughly and keep on ice:

**Table:** 

Reagent	Volume per reaction (μL)
Nuclease-free water	2
5X First Strand buffer	4
0.1 M DTT	1
RNasin	0.5
SuperScript III	0.5
Total	**8**
  f. Add 8 μL pre-assembled RT mix to each sample recovered from the thermocycler and mix well by pipetting.
  g. Return the samples back to the thermocycler and run the following program with the lid heated to the maximum temperature (usually 105°C):

**Table:** 

Steps	Temperature	Time	Cycle number
Initial incubation	25°C	5 min	1
cDNA synthesis	42°C	20 min	1
	50°C	40 min	–
Inactivation	85°C	5 min	1
Hold	16°C	∞	1

### RNA degradation

#### Day 3

**Timing: 30 min**

This section describes how to hydrolyze the RNA under alkaline conditions and at elevated temperature to remove it from the cDNA samples.

31. Hydrolyze RNA.
  a. Add 1.65 μL 1 M NaOH to each RT reaction and mix well.
  b. Place the samples back to the thermocycler and run the following program with the lid heated to the maximum temperature (usually 105°C):

**Table:** 

Steps	Temperature	Time	Cycle number
RNA degradation	98°C	20 min	1
Hold	25°C	∞	1

32. Neutralization.
  a. Add 20 μL 1 M HEPES to each sample recovered from the thermocycler and mix well.
  b. Transfer each sample to a non-stick 1.5 mL tube.

### Isolation of truncated cDNA with MyOne Silane beads

#### Day 3

**Timing: 30 min**

This section describes how to isolate the truncated cDNA using MyOne Silane beads.

33. Prepare MyOne Silane beads for isolation of truncated cDNA.
  a. Aliquot 10 μL MyOne Silane beads per sample from the original stock into a 1.5 mL tube.
  b. Wash the beads once with 750 μL RLT buffer.
  c. Resuspend the beads in 125 μL RLT buffer per sample.
34. Isolate truncated cDNA with MyOne Silane beads.
  a. Add 125 μL beads in RLT buffer to the samples from Step 32b and briefly mix by pipetting.
  b. Add 150 μL 100% ethanol to the samples and mix well by pipetting.
  c. Incubate the mixture at 20°C–24°C for 5 min.
  d. Pipette the mixture again and incubate at 20°C–24°C for additional 5 min.
  e. Place the samples on a magnetic rack to collect the beads and discard the supernatant.
  f. Wash the beads with 800 μL 80% ethanol by resuspending them.
  g. Transfer the beads in 80% ethanol to a new 1.5 mL non-stick tube.
  h. Place the samples on a magnetic rack to collect the beads and discard the supernatant.
  i. Wash the beads twice with 80% ethanol without resuspending.
  j. After the last wash, slowly lift the tube up on the magnetic rack to collect the beads at the bottom in a single spot.
  k. Discard the supernatant and air-dry the beads.
    ***Note:*** Do not over-dry the beads.
  l. Resuspend the beads in 5.3 μL nuclease-free water.

### Second adapter ligation to 3′ ends of truncated cDNA

#### Day 3

**Timing: 20 min**

This section describes how to ligate a 5′-phosphorylated DNA adapter containing unique molecular identifiers (5′UMIs) to the 3′ end of the cDNA at the truncation site.

35. Ligate second adapter to cDNA 3′ ends.
  a. Prepare the following master mix:

**Table:** 

Reagent	Volume per reaction (μL)
100% DMSO	1
20 μM iCLIP3 second adapter	1
Total	**2**
  b. Add 2 μL master mix to the beads from Step 34l.
  c. Heat the samples at 75°C for 2 min, then place on ice for 30 s and allow them to equilibrate to 20°C–24°C.
  d. Prepare the following additional master mix:

**Table:** 

Reagent	Volume per reaction (μL)
Nuclease-free water	2
10X RNA Ligase Reaction buffer	2
100 mM ATP	0.2
Total	**4.2**
  e. Add 4.2 μL additional master mix to the beads from Step 35c.
  f. Add 7 μL 50% PEG8000 and 1.5 μL T4 RNA Ligase 1 (high concentration).
  g. Mix the assembled ligation reactions thoroughly by pipetting.
    ***Note:*** Pipette the assembled ligation reactions slowly and thoroughly to ensure homogeneous distribution of all components. PEG8000 is highly viscous; avoid liquid and bead retention in the pipette tip.
  h. Incubate the ligation reactions in a thermomixer at 25°C for 16 h–20 h with shaking at 1,200 rpm.

### Isolation of the final cDNA with MyOne Silane beads

#### Day 4

**Timing: 30 min**

This section describes how to recover the final cDNA containing both adapters from the second adapter ligation reaction using MyOne Silane beads.

36. Prepare MyOne Silane beads for isolation of final cDNA.
  a. Aliquot 5 μL MyOne Silane beads per sample from the original stock in a 1.5 mL tube.
  b. Wash the beads once with 750 μL RLT buffer.
  c. Resuspend the beads in 60 μL RLT buffer per sample.
37. Isolate the final cDNA with MyOne Silane beads.
  a. Add 60 μL beads in RLT buffer to the ligation reaction from Step 35h and briefly mix by pipetting.
  b. Add 60 μL 100% ethanol to the samples and mix well by pipetting.
  c. Incubate the mixture at 20°C–24°C for 5 min.
  d. Pipette the mixture again and incubate at 20°C–24°C for additional 5 min.
  e. Place the samples on a magnetic rack to collect the beads and discard the supernatant.
  f. Wash the beads with 800 μL 80% ethanol by resuspending them.
  g. Transfer the beads in 80% ethanol to a new 1.5 mL non-stick tube.
  h. Place the samples on a magnetic rack to collect the beads and discard the supernatant.
  i. Wash the beads twice with 80% ethanol without resuspending.
  j. After the last wash, slowly lift the tube up on the magnetic rack to collect the beads at the bottom in a single spot.
  k. Discard the supernatants and airdry the beads.
    ***Note:*** Do not over-dqry the beads.
  l. Resuspend the beads in 15 μL nuclease-free water and incubate at 20°C–24°C for 5 min.
  m. Place the samples on a magnetic rack and transfer each supernatant (14.7μL) to a new 1.5 mL tube.
    **Pause point:** The isolated final cDNA can be stored at −20°C long term.

### Short-primer PCR (final cDNA pre-amplification)

#### Day 4

**Timing: 30 min**

This section describes how to amplify the final cDNA with short primers using a minimal number of PCR cycles. This short-primer PCR generates a short iCLIP3 library that is not yet ready for sequencing. The short iCLIP3 library requires subsequent size selection to remove contaminating adapter dimers.

38. Perform short-primer PCR.
  a. Assemble the following PCR reaction:

**Table:** 

Reagent	Volume per reaction (μL)
Final cDNA from Step 37m	14.7
Primer mix of P5s and P7s (50 μM each)	0.3
2X Phusion HF PCR MasterMix	15
Total	**30**
    **CRITICAL:** It is essential to keep the PCR reaction volume exactly at 30 μL.
  b. Run the PCR in a thermocycler using the following PCR program:

**Table:** 

Steps	Temperature	Time	Cycle number
Denaturation 1	98°C	30 s	1
Denaturation 2	98°C	10 s	5–7
Annealing	65°C	30 s	–
Extension	72°C	30 s	–
Final extension	72°C	3 min	1
Hold	16°C	∞	1
    ***Note:*** For abundant RBPs with known RBDs, perform 5 PCR cycles. For unknown RBPs, perform 6 PCR cycles. For proteins that bind RNA weakly or transiently, 7 PCR cycles may be required.

### Short iCLIP3 library size selection with ProNex beads

#### Day 4

**Timing: 1.5 h**

This section describes how to size-select the short iCLIP3 library using ProNex beads. This step removes adapter dimers (∼50 bp) and recovers the library ≥ 75 bp (insert size: ≥ 25 bp), with the most efficient recovery for library ≥ 100 bp (insert size: 50 bp). Ultra Low Range (ULR) DNA ladder is first used to evaluate ProNex-based size selection.

39. Equilibrate ProNex beads and ProNex Wash buffer (with ethanol added) at 20°C–24°C for 30 min prior to use.
40. Size-select ULR DNA ladder.
  a. Set up the size selection ULR ladder control.

**Table:** 

Reagent	Volume per reaction (μL)
ULR DNA ladder	1
Nuclease-free water	14
2X Phusion HF PCR MasterMix	15
Total	**30**
  b. Set up the reference ULR ladder and keep at 4°C until further use.

**Table:** 

Reagent	Volume per reaction (μL)
ULR DNA ladder	1
Nuclease-free water	22
Total	23
  c. Vortex the ProNex bead stock solution and mix thoroughly by pipetting.
  d. Add 2.95 volumes of ProNex beads (88.5 μL) to the size selection ULR ladder control from Step 40a.
  e. Mix well by pipetting up and down 8–10 times.
  f. Incubate the mixture at 20°C–24°C for 10 min.
  g. Place the tube on a magnetic rack to collect the beads.
  h. Discard the supernatants.
  i. Add 150 μL ProNex Wash buffer without disturbing the beads and incubate 15 s.
  j. Repeat steps h and i.
  k. Discard the supernatant and air-dry the beads until bead cracking becomes apparent.
    ***Note:*** Beads can be dried for 1–2 min at 37°C. Do not over-dry the beads.
  l. Remove the tube from the magnetic rack and resuspend the beads in 23 μL nuclease-free water.
  m. Incubate the sample for 5 min at 20°C–24°C.
  n. Place the tube on a magnetic rack to collect the beads.
  o. Transfer the eluted size-selected ULR ladder control to clean 1.5 mL tubes.
    **Pause point:** The eluted size-selected ULR ladder control can be stored at −20°C long term.
41. Evaluate ProNex size selection using the TapeStation.
  a. Equilibrate TapeStation reagents at 20°C–24°C for 20–30 min prior to use.
  b. Load 2 μL reference ULR ladder from Step 40b and 2 μl size-selected ULR ladder control from Step 40o onto the TapeStation using High Sensitivity D1000 ScreenTape.
  c. Assess the recovery of the 50 bp, 75 bp and 100 bp bands in the size-selected ULR ladder control relative to the reference ULR ladder.

**Figure 4:**
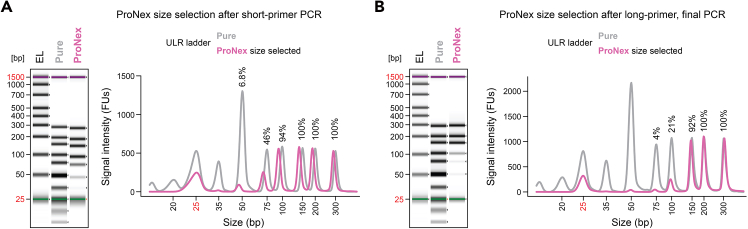
iCLIP3 library size selection (A) ProNex bead-based size selection of dsDNA after short-primer PCR. Capillary gel electrophoresis image of the pure (grey) and size-selected ULR ladder (pink) using ProNex beads at sample-to-ProNex bead ratio of 1:2.95. (B) ProNex bead-based size selection of dsDNA after final long-primer PCR. Capillary gel electrophoresis image of the pure (grey) and size-selected ULR ladder (pink) using ProNex beads, PEG8000/NaCl mixture and ethanol. For both (A) and (B), accompanying partial electrograms of the capillary gel electrophoresis are shown. The numbers above peaks represent the percent recovery of each peak after ProNex bead size selection.

***Note:*** Successful size selection is indicated by significant removal of the 50-bp band, ∼50% recovery of the 75-bp band, and ∼100% recovery of the 100-bp band (Figure 4A). Additionally, the ratio of the 75-bp to 50-bp band intensities in the size-selected ULR ladder control should be ≥ 2.5.

42. Size-select the short iCLIP3 library with ProNex beads.
  a. If ULR DNA ladder size selection was successful (see Step 41 Note, Figure 4A), subject the short-primer PCR reaction from Step 38b to ProNex size-selection by following Steps 40c-o.
  b. Store the eluted and size-selected short iCLIP3 library at 4°C for ≤24 h or directly proceed to the next step.

**Pause point:** The eluted size-selected short iCLIP3 library can be stored at −20°C long term.

### Long-primer test PCR

#### Day 4

**Timing: 30 min**

This section describes how to amplify an aliquot of the short iCLIP3 library using long PCR primers containing the *i5* and *i7* index sequences. These sequences enable binding to the Illumina flow cell and cluster generation during sequencing. Performing this test PCR on a small aliquot of the short iCLIP3 library allows both qualitative and quantitative assessment of the resulting test iCLIP3 library.

43. Perform long-primer test PCR.
  a. Assemble the following PCR reaction:

**Table:** 

Reagent	Volume per reaction (μL)
Short iCLIP3 library from Step 42b	1
Primer mix of P5L and P7L (10 μM each)	0.5
2X Phusion HF PCR MasterMix	5
Nuclease-free water	3.5
Total	**10**
  b. Run the PCR in a thermocycler using the following PCR program:

**Table:** 

Steps	Temperature	Time	Cycle number
Denaturation 1	98°C	30 s	1
Denaturation 2	98°C	10 s	8–15
Annealing	65°C	30 s	–
Extension	72°C	30 s	–
Final extension	72°C	3 min	1
Hold	16	∞	1
    ***Note:*** Perform 2–3 PCR reactions for each sample using different cycle numbers (e.g., 8, 10, and 12 cycles) to determine the optimal amplification conditions for each library.

### Test iCLIP3 library size selection with ProNex beads

#### Day 4

**Timing: 30 min**

This section describes how to size-select the test iCLIP3 library using ProNex beads. This step removes unused long PCR primers and recovers test iCLIP3 library fragments ≥ 170 bp (insert size: ≥ 25 bp).

44. Equilibrate ProNex beads and ProNex Wash buffer (with ethanol added) at 20°C–24°C for 30 min prior to use.
45. Size-select the test iCLIP3 library.
  a. Vortex ProNex bead stock and mix thoroughly by pipetting.
  b. Add 2.4 volumes of ProNex beads (24 μL) to the PCR reaction from Step 43b.
  c. Mix well by pipetting up and down 8-10 times.
  d. Incubate the mixture at 20°C–24°C for 10 min.
  e. Place the tubes on a magnetic rack to collect the beads.
  f. Discard the supernatants.
  g. Add 120 μL ProNex Wash buffer without disturbing the beads and incubate 15 s.
  h. Repeat steps f and g.
  i. Discard the supernatant and air-dry the beads until cracking becomes apparent.
    ***Note:*** Do not over-dry the beads.
  j. Remove the tubes from the magnetic rack and resuspend the beads in 12 μL nuclease-free water.
  k. Incubate the samples for 5 min at 20°C–24°C.
  l. Place the tubes on a magnetic rack to collect the beads.
  m. Transfer the eluted test iCLIP3 libraries to clean 1.5 mL tubes.
    **Pause point:** The isolated test iCLIP3 libraries can be stored at 4°C for ≤24 h or at −20°C long term.

### Test iCLIP3 library quality control and quantification

#### Day 4

**Timing: 30 min**

This section describes how to evaluate the quality and purity of the test iCLIP3 library and determines its concentration.

46. Evaluate quality of the test iCLIP3 library using the TapeStation.
  a. Equilibrate Tape Station reagents at 20°C–24°C for 20–30 min prior to use.
  b. Load 2 μL purified test iCLIP3 library from Step 45m onto the TapeStation using High Sensitivity D1000 ScreenTape.
  c. Visualize library size distribution and confirm removal of PCR primers.
  d. Determine the average size of the library in bp. The purified library typically appears as a dsDNA smear ≥ 175 bp.
47. Quantify the test iCLIP3 library.
  a. Use 1–2 μL purified test iCLIP3 library from Step 45m to measure the DNA concentration (ng/μL) using a Qubit fluorimeter according to the manufacturer’s instructions.
  b. Calculate library molarity using the following equations:

$$Library\;Mw\;\left(\frac{g}{mol}\right)=Library\;\left(bp\right)\times 660\;\left(\frac{g}{mol}\right)$$

$$Library\;molarity\;\left(nM\right)=\frac{Library\;concentration\;\left(\frac{ng}{\mu L}\right)\times 10^{6}\;}{Library\;average\;Mw\;\left(\frac{g}{mol}\right)}$$

### Long-primer final PCR cycle determination and primer selection

#### Day 4

**Timing: 30 min**

This section describes how to determine the optimal number of PCR cycles for final library amplification. During the final PCR, each sample is labeled with unique dual indexes (UDIs) incorporated into the long primers. Carefully select UDIs to ensure proper color balance and sufficient Hamming distance between indexes for multiplexing, thereby minimizing index hopping during sequencing.

48. Calculate the number of PCR cycles required for final library amplification using the following equation:

$$Nf=Nt+\log\;2\left(\frac{10}{Mt\;\left(nM\right)\times cDNA\;\left(\mu L\right)}\right)$$

where *Nf* is the number of cycles for the final PCR, *Nt* is the number of cycles used in the test PCR, *Mt* is the calculated molarity from the test PCR, and cDNA is the volume of the pre-amplified cDNA (short iCLIP3 library) in the final PCR reaction.

49. Select unique dual index (UDI) primer pairs for final PCR amplification of individual iCLIP3 libraries using the DNA Barcode Combination Finder Shiny app to identify compatible barcode combinations and ensure appropriate index color balance for Illumina sequencing.
  ***Note:*** The app takes as input the available *i7* (and optionally *i5*) index sequences, either from the built-in iCLIP3 index library or from user-provided custom index sets. It then calls the `experimentdesign` function from the R package DNABarcodeCompatibility^17^ to compute sets of barcode combinations that maintain a minimal pairwise distance (Hamming or Levenshtein) between indexes and thus reduce the risk of index misassignment or hopping. In addition, for Illumina platforms using XLEAP SBS chemistry (NextSeq 1000/2000 and NovaSeq X/X Plus), the app evaluates base composition per index cycle and iteratively searches for combinations that satisfy Illumina’s color-balance constraints (avoiding cycles with only A/G or only G signal, while allowing T/C-only cycles).
  a. Specify whether libraries will be single-indexed (*i7* only) or dual-indexed (*i7*+*i5*), and choose whether to use the iCLIP3 index library or paste custom index sequences (one per line).
  b. Enter the total number of libraries (samples), the desired multiplexing level per lane, and the number of color channels corresponding to the sequencing platform (e.g., 2 for NextSeq/NovaSeq).
  c. Optionally set advanced parameters by choosing the type and value for the distance metric (Hamming or sequence Levenshtein), which controls how dissimilar individual indexes must be.
  d. Run the analysis to obtain a table of optimal *i7* only or *i7*/*i5* UDI combinations, grouped by lane, along with summary tables showing per-cycle Index color balancing and sequence logos visualizing nucleotide-specific color signal and base usage across indexes.
    The resulting table directly lists the recommended P5/P7 long primer pairs (containing *i5*/*i7* indexes) that should be used for the final PCR amplification of each iCLIP3 library.

### Long-primer final PCR

#### Day 4 or 5

**Timing: 30 min**

This section describes how to amplify the final iCLIP3 library using the previously selected primer combinations in Step 49. This amplification yields a finalized library ready for sequencing on Illumina platforms.

50. Perform long-primer, final PCR.
  a. Assemble the following PCR reaction:

**Table:** 

Reagent	Volume per reaction (μL)
Short iCLIP3 library from Step 42b	10
Primer mix of P5L and P7L (10 μM each)	2
2X Phusion HF PCR MasterMix	20
Nuclease-free water	8
Total	**40**
    **CRITICAL:** Maintain the PCR reaction volume exactly at 40 μL.
  b. Run the PCR in a thermocycler using the following PCR program:

**Table:** 

Steps	Temperature	Time	Cycle number
Denaturation 1	98°C	30 s	1
Denaturation 2	98°C	10 s	n
Annealing	65°C	30 s	–
Extension	72°C	30 s	–
Final extension	72°C	3 min	1
Hold	16	∞	1

### Final iCLIP3 library size selection with ProNex beads

#### Day 4 or 5

**Timing: 1.5 h**

This section describes how to purify and eliminate unused long PCR primers using ProNex beads. Ultra Low Range (ULR) DNA ladder is first used to evaluate ProNex-based size selection.

51. Equilibrate ProNex beads and ProNex Wash buffer (with ethanol added), as well as PEG/NaCl Mix at 20°C–24°C for 30 min.
52. Size-select ULR DNA ladder.
  a. Set up the size selection ULR ladder control.

**Table:** 

Reagent	Volume per reaction (μL)
Nuclease-free water	19
ULR DNA ladder	1
2X Phusion HF MasterMix	20
Total	**40**
  b. Set up the reference ULR ladder and keep at 4°C until further use:

**Table:** 

Reagent	Volume per reaction (μL)
ULR DNA ladder	1
Nuclease-free water	11
Total	**12**
  c. Prepare the following size selection mixture and pipette thoroughly to ensure proper mixing:

**Table:** 

Reagent	Volume per reaction (μL)
Nuclease-free water	2.5
**PEG/NaCl Mix**	12.5
ProNex beads	25
Total	**40**
  d. Add 40 μL size selection mixture to the size selection ULR ladder control from Step 52a.
  e. Add 20 μL 100% ethanol to the sample and mix by pipetting up and down 8-10 times.
  f. Incubate the mixture for 10 min at 20°C–24°C.
  g. Place the tube on a magnetic rack to collect the beads.
  h. Discard the supernatant.
  i. Add 150 μL ProNex Wash buffer without disturbing the beads and incubate for 15 s.
  j. Repeat steps h and i.
  k. Discard the supernatant and air-dry the beads until cracking becomes apparent.
    ***Note:*** Do not over-dry the beads.
  l. Remove the tube from the magnetic rack and resuspend the beads in 12 μL nuclease-free water.
  m. Incubate the tube for 5 min at 20°C–24°C.
  n. Place the tube on the magnetic rack to collect the beads.
  o. Transfer the eluted final size-selected ULR ladder control to clean 1.5 mL tubes.
    **Pause point:** The isolated size-selected ULR ladder can be stored at −20°C long term.
53. Evaluate ProNex size selection using the TapeStation.
  a. Equilibrate TapeStation reagents at 20°C–24°C for 20–30 min prior to use.
  b. Load 2 μL reference ULR ladder from Step 52b and 2 μl size-selected ULR ladder control from Step 52o onto the TapeStation using High Sensitivity D1000 ScreenTape.
  c. Assess the recovery of the 75-bp, 100-bp and 150-bp bands in the size-selected ULR ladder control relative to the reference ULR ladder.

***Note:*** Successful size selection is indicated by significant removal of the 75-bp band and ≥ 90% recovery of the 150-bp band (Figure 4B). Additionally, the ratio of the 150-bp to 75-bp band intensities in the size-selected ULR ladder control should be ≥ 15.

54. Size-select the final iCLIP3 library with ProNex beads.
  a. If ULR DNA ladder size selection was successful (see Step 53 Note, Figure 4B), subject the long-primer PCR reaction from Step 50b to ProNex size selection by following Steps 52c-o.
  b. Store the eluted and size-selected final iCLIP3 library at 4°C for ≤24 h or directly proceed to the next step.

**Pause point:** The eluted size-selected final iCLIP3 library can be stored at −20°C long term.

### Final iCLIP3 library quality control and quantification

#### Day 4 or 5

**Timing: 30 min**

This section describes how to evaluate the quality, purity and concentration of the final iCLIP3 library.

55. Evaluate quality of the final iCLIP3 library using the TapeStation.
  a. Equilibrate TapeStation reagents at 20°C–24°C for 20–30 min prior to use.
  b. Load 2 μL final iCLIP3 library from Step 54b on the TapeStation using High Sensitivity D1000 ScreenTape.
  c. Visualize the library size distribution and confirm the removal of PCR primers.
  d. Determine the average library size for each sample in bp. A properly purified library appears as a dsDNA smear ≥ 175 bp (Figure 5).

**Figure 5:**
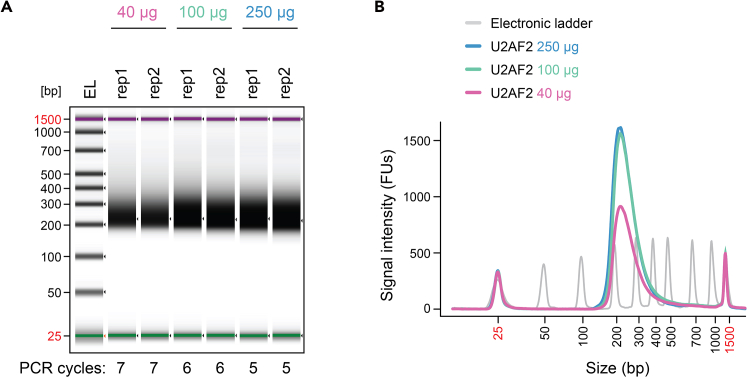
Generation of U2AF2 iCLIP3 libraries from different amounts of input material (A) TapeStation capillary gel electrophoresis of the final iCLIP3 libraries prepared from the samples depicted in Figure 3D. The number of cycles used in the second, final PCR is shown. (B) An accompanying electrogram of the capillary gel electrophoresis from (A). The average signal intensity values from two replicates are displayed. FUs, fluorescent units.
56. Quantify final iCLIP3 library.
  a. Use 1 μL final iCLIP3 library from Step 54b to measure the concentration (ng/μL) for each sample with a Qubit fluorimeter according to the manufacturer’s instructions.
  b. Using the equations from Step 47b, determine the molarity of each individual iCLIP3 library.

**Pause point:** The isolated final iCLIP3 libraries can be stored at −20°C long term.

### iCLIP3 library multiplexing

#### Day 4 or 5

**Timing: 20 min**

This section describes how to combine multiple individual iCLIP3 libraries at an equimolar ratio to enable sequencing on the same Illumina flow cell lane. Equimolar pooling ensures comparable sequencing depth for each library within the pooled sample.

57. Using the library molarity calculated in Step 56b, mix the individual libraries at equimolar ratios to create the final multiplexed library pool.

### Pooled iCLIP3 library cleanup with ProNex beads

#### Day 4 or 5

**Timing: 30 min**

This section describes how to remove residual long PCR primers from the pooled iCLIP3 library to ensure a clean library for sequencing.

58. Purify the pooled iCLIP3 library by following Steps 44–45 of this protocol.

**Pause point:** The purified pooled iCLIP3 library can be stored at −20°C long term until sequencing.

### Pooled iCLIP3 library quality control and quantification

#### Day 4 or 5

**Timing: 30 min**

This section describes how to assesses the quality, size distribution and concentration of the pooled iCLIP3 library to verify its suitability for sequencing.

59. Evaluate quality and concentration of the pooled iCLIP3 library.
  a. Evaluate the quality of the pooled iCLIP3 library from Step 58 using the TapeStation to visualize the library size distribution and confirm the removal of residual primers (follow Step 55).
  b. Measure the concentration of the pooled iCLIP3 library from Step 58 using Qubit fluorimeter or an equivalent instrument (follow Step 56).

### iCLIP3 library sequencing

60. Sequence each iCLIP3 library within the pool to a depth of 30-50 million reads on Illumina sequencer or an equivalent sequencer using a single-end configuration with 75 or 100 cycles.

### Installation of racoon_clip for iCLIP3 data analysis

**Timing: 1–2 h**

To analyze the sequencing data, we employ racoon_clip,^6^ a fully automated workflow for processing iCLIP3, earlier iCLIP variants and eCLIP data to extract RBP-RNA crosslink positions at single-nucleotide resolution. This section describes how to install racoon_clip.

61. Install racoon_clip on a UNIX-based compute cluster.
  a. Build racoon_clip in a conda environment from the GitHub release.
    i. Make a fresh conda environment containing the racoon_clip dependencies mamba, python and pip.
      bash
      # make racoon_clip conda environment with mamba support
      >conda create -n racoon_clip ∖
       --override-channels -c conda-forge ∖
       mamba=1 ∖
       'python_abi=∗=∗cp∗' ∖
       python=3.9.0 ∖
       pip=25.0
      # activate conda environment
      >conda activate racoon_clip
    ii. Find the latest version at ZarnackGroup GitHub and copy the link to the ZIP file of the latest version.
    iii. Download and install racoon_clip in this environment.
      ***Note:*** Replace [version] with the latest version number (e.g., v.2.0.12).
      ***Note:*** racoon_clip is also available as ready-made containers based on Docker or Apptainer. For more details, refer to the racoon_clip documentation. SingularityCE is currently not supported.
      bash
      # download racoon_clip
      >wget link/to/latest/release.zip
      >unzip [version].zip
      >cd racoon_clip-[version]
      # install
      pip install -e .
  b. Check that racoon_clip works by getting the version number.
    ***Note:*** In this protocol, we are using version v.2.0.12.
    bash
    >racoon_clip -v
    ***Optional:*** Test racoon_clip.
    bash
    >racoon_clip --light
    ***Note:*** This runs a quick test, including basic functionality checks and should output the message “All tests passed successfully” in the end. If not, see the troubleshooting section below.

### Preparation of genome input files

**Timing: 1–2 h**

62. Download a suitable genome (FASTA format) and a matching genome annotation in GTF format.

bash

# download genome FASTA and annotation GTF

>wget link_to_genome.fa.gz

>wget link_to_genome_annotation.gtf.gz

***Note:*** For human or mouse data, we recommend using the comprehensive gene annotation for the primary genome assembly (PRI) from GENCODE.

63. Optional: Use racoon_clip’s FastQ Screen module to screen for potential RNA contamination from other organisms and/or rRNA content.
  a. Use the following code to get FASTA files of all contaminant genomes you want to screen against.
    bash
    # download genomes to screen against
    >wget link_to_genome_for_screening.fa.gz
    >fa=<path/to/genome_for_screening.fa.gz>
  b. For rRNA content, obtain a FASTA file from the rRNA sequences.
    ***Note:*** A FASTA file of the human rRNA sequences used in this study is available on GitHub. Alternatively, create a custom FASTA file as described below.
  c. Create an rRNA content FASTA as follows:
    i. Make a new empty FASTA file (e.g., rRNA.fa).
    ii. Copy the rRNA sequences for example from NCBI into the FASTA file. Follow the FASTA format as shown here.
      content of rRNA.fa
      >28S_rRNA
      CGCGACCTCAGATCAGACGTGGCGACCCGCTGAATTTAAGCATA.
      >18S_rRNA
      ***Note:*** For the human genome, we used the rRNA genes from NCBI with the following NCBI/RefSeq IDs: 28S, NR_003287.4; 18S, NR_003286.4; 5.8S, NR_003285.3; 5S, NR_023363.1; mitochondrial 12S (MT-RNR1), GeneID 4549; mitochondrial 16S (MT-RNR2), GeneID 4550.
64. Generate genome indices for mapping.
  a. Install necessary packages in a conda environment.
    bash
    # install bowtie and gffread
    >conda create -n bowtie_index bioconda::bowtie2 >bioconda::gffread
    >conda activate bowtie_index
    >bowtie2 -h
    >gffread -h
  b. Index the genomes with bowtie2.
    bash
    # make bowtie index (for rRNA)
    >bowtie2-build rRNA.fa > rRNA_idx
    # make bowtie index (for contaminating organisms)
    >bowtie2-build contaminant_genome.fa > contaminant_genome_idx
65. If you want to screen for multiple contaminants and/or the rRNAs of multiple organisms, repeat the steps 63 and 64 for each of them.
66. Create a configuration file named fastqscreen.config with all contaminants to screen against.
  a. Use the following space-separated structure (DATABASE <name_of_contaminant> <path_to_folder_containing_bowtie_index/prefix_of_bowtie_index>).

***Note:*** Add a new line with the same structure for each contaminant to be screened.

content of fastqscreen.config

DATABASE contaminantA </path/to/contaminantA_idx>

DATABASE rRNA </path/to/rRNA_idx>

### Obtaining crosslink events and peaks from sequencing data using racoon_clip

**Timing: 2–3 days**

This section describes how to process raw sequencing data using the command-line tool racoon_clip.

**Figure 6:**
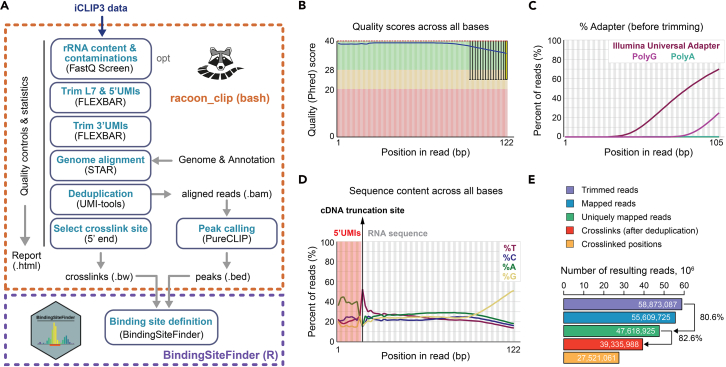
Processing of iCLIP3 sequencing data (A) Computational analysis workflow for iCLIP3 data using the command-line tool racoon_clip and the R/Bioconductor package BindingSiteFinder. The pipeline begins with preprocessing steps, include optional quality control and trimming of UMIs and sequencing adapters, that are tailored to different CLIP protocols to ensure correct parsing of read structures. Processed reads are then aligned to the reference genome in a splice-aware manner using STAR (Dobin et al., 2013) with parameters optimized to preserve exact 5′ ends, followed by UMI-based deduplication to remove PCR duplicates. Crosslink sites are subsequently defined as the nucleotide immediately upstream of aligned read 5′ ends and exported as single-nucleotide resolution tracks in bigWig format. In the latest version used here, the workflow is extended by automated peak calling with PureCLIP to identify crosslink positions significantly enriched over local background; these peaks form the basis for the downstream binding site definition (see Methods S2). In addition, racoon_clip generates a comprehensive HTML report summarizing data quality metrics and processing statistics. (B) Distribution of sequencing quality (Phred) scores at each nucleotide position along iCLIP3 reads. (C) Adapter content before trimming. Plot shows percent of reads with universal Illumina adapter, polyG or polyA tracts at each nucleotide position in reads. (D) Sequence composition of reads. Shown is the percent of each nucleotide at each nucleotide position. Position of 9-nt 5′UMI and cDNA truncation site are highlighted. (E) Number of reads after each racoon_clip processing step. (B–E) show exemplary visualizations (modified from racoon_clip report) for replicate 1 of 250 μg U2AF2 iCLIP3. The full processing report for all U2AF2 iCLIP3 samples is available in Data S1. racoon_clip uses FastQC for (B-D).

***Note:*** racoon_clip performs quality checks, adapter and UMI trimming, genomic alignment, deduplication and extraction of both crosslink events and peaks at single-nucleotide resolution (Figure 6A). Refer to the racoon_clip documentation for the default parameters used in each step.

***Note:*** Scripts and configuration files used for the U2AF2 iCLIP3 data in this publication are available on GitHub.

67. Prepare the racoon_clip input files.
  a. Copy the raw demultiplexed FASTQ sequencing files to the compute cluster.
    ***Note:*** racoon_clip can handle gzipped or unzipped FASTQ files. We recommend comparing the md5sums to ensure that the transfer was complete.
    ***Optional:*** Make an adapter file (here named adapter.fa) that contains the sequence of the L7 adapter:
    content of adapter.fa
    >adapter
    AGATCGGAAGAGCACACGTC
    ***Note:*** If you are using an adapter that differs from the one used in this protocol, add the sequence of your adapter instead.
  b. Make a racoon_clip config file (here called config.yaml) in YAML format that specifies all input files and selected parameters.
    i. A basic racoon_clip config file looks like this:
      content of racoon_config.yaml
      # output directory
      wdir: “output/path”
      # input
      infiles: “path/to/sample1.fastq path/to/sample2.fastq”
      samples: “sample1 sample2”
      # annotation
      gtf: “path/to/annotation.gtf”
      genome_fasta: “path/to/genome_assembly.fa”
      read_length: N
      # experiment type
      experiment_type: “iCLIP3”
      # adapter
      adapter_trimming: True
      adapter_file: “path_to/adapter.fa”
    ii. Fill the config file as follows:

**Table:** 

parameter	input	notes
wdir	folder where output files should be written	in quotation marks; no slash at the end
infiles	paths to all input files separated by empty space	quotation marks before first and after last path
samples	sample names	must be identical to the file names without the file ending (.fastq or.fastq.gz); quotation marks before first and after last sample name
GTF	path to downloaded annotation GTF file	in quotation marks; has to be unzipped
genome_fasta	path to downloaded genome assembly	in quotation marks; has to be unzipped
read_length	length of sequencing reads	no quotation marks
experiment_type	“iCLIP3”	Experiment type refers to the UMI + barcode architecture of the experiment. If your UMI + barcode architecture diverges from the one described here, racoon_clip offers other experiment_types as well as an option to use a custom architecture. For an explanation of experiment_types, check out the racoon_clip documentation.
adapter_trimming	True	no quotation marks
adapter_file	path to adapter_file.fa	in quotation marks
      ***Note:*** For racoon_clip, the term “sample” refers to a set of reads sharing the same barcode, i.e., a single fastq file. This differs from the usage of the terms “sample” and “replicate” in other sections of this protocol. For the U2AF2 iCLIP3 data analyzed here, racoon_clip “samples” correspond to the individual replicates (rep1, rep2) of all conditions (250μg, 100μg, 40μg).
      ***Optional:*** Customize racoon_clip by adding additional parameters as extra lines to the config file.
      ***Note:*** The following lists the commonly used parameters. A complete description can be found in the racoon_clip documentation.
      ***Optional:*** Customize by adding experiment groups.
      ***Note:*** If your data contains multiple conditions or groups, you can perform peak calling separately within each group.
    iii. Prepare a group TXT file (here named groups.txt) to assign each sample to a group. The group file should have one line per sample and specify “group_name sample” in each line (separated by a space).
      **CRITICAL:** The sample names must exactly be the file names without file ending (.fastq or.fastq.gz). Group names may be chosen freely.
      ***Note:*** The following example shows the group file for the U2AF2 iCLIP3 data from this study:
      content of groups.txt
      u2af2_40ug u2af2_40ug_rep1.R1
      u2af2_40ug u2af2_40ug_rep2.R1
      u2af2_100ug u2af2_100ug_rep1.R1
      u2af2_100ug u2af2_100ug_rep2.R1
      u2af2_250ug u2af2_250ug_rep1.R1
      u2af2_250ug u2af2_250ug_rep2.R1
      ***Note:*** If you do not specify a group file, peak calling will be performed on a merge of all samples.
    iv. Add the following to the config.yaml.
      optional lines for racoon_config.yaml
      experiment_group_file: “path/to/groupfile.txt”
      ***Optional:*** Customize by adding contamination screening.
    v. Set the parameter fastqScreen to True.
    vi. Provide the path to the fastqscreen.config file to the parameter fastqScreen_config.
      optional lines for racoon_config.yaml
      fastqScreen: True
      fastqScreen_config: “path_to/fastqscreen.config
      ***Optional:*** Customize by restricting PureCLIP training on selected chromosomes (Recommended).
      ***Note:*** Peak calling with PureCLIP^18^ is the most time and memory-consuming step of racoon_clip. PureCLIP first trains a hidden Markov model on the data and then calls peaks using the learned pattern. Training is by default done on the complete genome, but can be downsized to a few chromosomes, as also recommended in the PureCLIP documentation.
    vii. Add the PureCLIP training chromosome parameter to the config file.
    viii. Specify chromosome names as a semicolon-separated list.
      optional lines for racoon_config.yaml
      morePureclipParameters: “-iv 'chr1;chr2;chr3;'”
      ***Note:*** Chromosome naming might differ depending on the genome and source (e.g., '1;2;3;' is often used by ENSEMBL).
      ***Note:*** The results shown in Figures 6 and 7 were obtained by training on the full genome.
68. Run racoon_clip by specifying the path to the config file and the number of available CPUs.
  bash
  >racoon_clip peaks --configfile <your_configfile.yaml> --cores <n_cores>
  ***Note:*** This step will likely take > 36 h. Increasing <n_cores> can speed this up to a certain extent. As a rule of thumb, specify the number of CPUs as 1x, 2x or 4x the number of samples. For the U2AF2 iCLIP3 data, computation time for all samples was around 2 days with 20 CPUs.
  ***Optional:*** Run racoon_clip using a SLURM cluster profile.
  a. Specify the desired cluster profile.
  b. Submit racoon_clip processing steps as individual jobs.
    ***Note:*** Instructions are available in the racoon_clip documentation.
69. Locate the results folder generated by racoon_clip in the output directory specified in the config file.
  a. Download Report.html to review processing summaries and quality-control metrics.
  b. Download the bw folder containing sample-specific crosslink events in bigWig format.
  c. Download the bw_merged folder containing merged crosslink events for each group (or all samples if no group file was specified).
  d. Download the peaks folder containing PureCLIP peak calls in BED format.
70. Review the Report.html file to assess the data quality and evaluate the results of each processing step.

**Figure 7:**
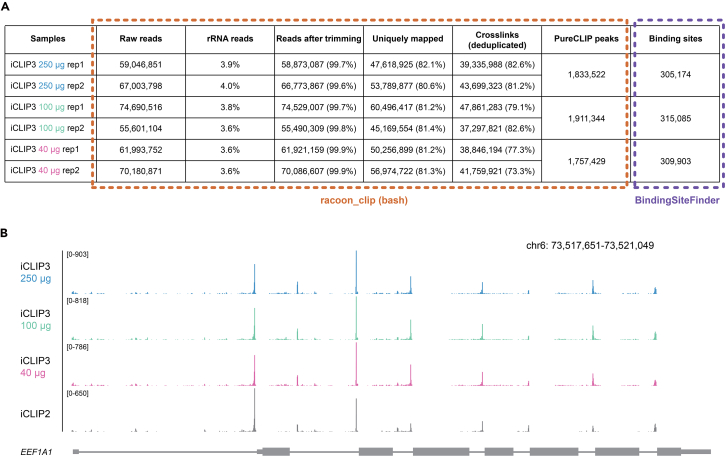
Comparison between U2AF2 iCLIP3 samples Data were individually processed for U2AF2 iCLIP3 from HeLa cell lysates containing 250 μg, 100 μg, and 40 μg of total protein. (A) Number of sequenced and mapped reads, PureCLIP-called peaks (from racoon_clip) and binding sites (from BindingSiteFinder) for all U2AF2 iCLIP3 samples. Percentages correspond to the difference from the prior step. Peak calling and binding site definition were performed on the merge of replicates for each condition. (B) Genome browser view of U2AF2 crosslink events (sum of replicates) across the gene *EEF1A1* (chr6:73,517,651-73,521,049) of U2AF2 iCLIP3 and iCLIP2 experiments. Axis scale is depicted on the left.

### Identification of binding sites using BindingSiteFinder

71. After extracting the crosslink events with racoon_clip, use the R/Bioconductor package BindingSiteFinder^8^ to define discrete RBP binding sites.

***Note:*** A detailed step-by-step protocol for using BindingSiteFinder is provided as a separate Protocol in Methods S2.

## Expected outcomes

Using this protocol, iCLIP3 libraries were successfully prepared for the RNA-binding protein U2AF2 from HeLa cell lysates containing 250 μg, 100 μg, and 40 μg of total protein. Long-primer final PCR amplification with 5, 6, and 7 cycles yielded high-quality libraries suitable for sequencing (Figure 5).

iCLIP3 libraries are expected to be ≥ 175 bp, with a majority of the signal falling within a size range of 175–300 bp (Figure 5). No signal should be detectable in negative control samples, including UV– and UV+ non-immune IgG or no-antibody (beads-only) controls, indicating minimal background and high specificity of the procedure. Experiments in which the long-primer final PCR exceeds 12 cycles are expected to yield substantial levels of PCR duplicates and may therefore not be suitable for downstream analyses.

racoon_clip generates a full report (in HTML format) that documents the performance and data quality across the analysis workflow. Representative visualizations for sequencing quality, adapter content, sequence composition and genomic mapping results for one sample are shown in Figure 6. The full processing report for all U2AF2 iCLIP3 samples is available in Data S1.

U2AF2 iCLIP3 libraries derived from HeLa cell lysates containing 250 μg, 100 μg, and 40 μg total protein were sequenced on an Illumina NextSeq 2000 as 122 nt single-end reads, yielding 56-75 million reads per sample. Analysis of the iCLIP3 samples shows that—irrespective of the amount of input material—a similar library depth was reached (Figure 7A). racoon_clip detected more than 35 million crosslink events per sample. Although this number cannot be generalized, at least 1 million crosslink events can be expected—as a rule of thumb—for good RNAbinders in mammalian cells. If the signal depth of an iCLIP3 dataset is much lower, it is recommended to revisit the quality controls performed during the experiment and data analysis.

The main outputs of racoon_clip are the bigWig files containing the crosslink events for each sample, as well as files merged across all samples (or within each group if specified). The crosslink positions correspond to the nucleotide position 1 nt upstream of the reverse transcription termination sites. The crosslink tracks can be loaded into a genome browser (e.g., Integrative Genomics Viewer, IGV) for manual inspection and comparison between samples and replicates (Figure 7B; Figure S3).

## Limitations

Like other CLIP-based protocols, iCLIP3 depends on the availability of high-quality IP-grade antibodies, which can limit its application to proteins for which suitable antibodies are lacking. In such cases, epitope tagging of the protein of interest can help overcome this limitation. iCLIP3 is also constrained by the inherently low efficiency of UV irradiation to induce covalent crosslinking between proteins and RNA; consequently, some RBPs may fail to crosslink efficiently despite bona fide interactions with RNA. For these proteins, metabolic labeling with 4-thiouridine (4sU) followed by 365 nm UV crosslinking may improve crosslinking efficiency, but different computational analysis of the data are required.^19^^,^^20^ Importantly, the iCLIP3 protocol cannot identify binding sites of cellular proteins whose interactions with RNA do not involve direct protein–RNA contact. In such cases, alternative approaches such as RIP-seq can provide complementary insights into RNA–protein associations.

racoon_clip requires 120 GB of RAM during the genomic mapping step. Therefore, it cannot be run on most local computers, but requires access to a compute cluster, cloud computing or a dedicated high-performance workstation.

A reference genome and annotation in standard GTF format are required for both the initial data processing with racoon_clip and the subsequent binding site definition with BindingSiteFinder (see Methods S2). These are not always available, particularly for non-model organisms.

## Troubleshooting

### Problem 1

No PCR libraries are observed in the test PCR with 12 cycles (related to Steps 50, 54–55).

### Potential solution

Increase the number of PCR cycles (Step 50). The iCLIP3 library is likely derived from negligible amounts of immunopurified RNA. Although additional PCR cycles may produce a visible library with sufficient yield, a high rate of PCR duplication is expected. For optimal results, repeat the experiment using a substantially larger amount of starting material, such as a higher number of cells.

### Problem 2

The peak observed in the test PCR is around 150 bp, indicating the absence of cDNA inserts or the presence of only very short inserts (replated to Steps 43–47).

### Potential solution

This issue likely reflects a failure during library preparation. Possible causes include insufficient input material, suboptimal reaction conditions, or RNA overdigestion by RNase I. Excessive RNase I activity could lead to RNA degradation and, consequently, very short or absent cDNA inserts. To improve outcomes, the experiment should be repeated using a substantially larger amount of starting material, such as a higher number of cells, or by optimizing RNase I-based RNA fragmentation (see Methods S1). If the problem persists, the reagents should be replaced and aliquoted to avoid repeated freeze–thaw cycles.

### Problem 3

Higher-molecular-weight bands are visible in the library profiles after long-primer test or final PCR (related to Steps 43-47, 50, 54-55).

### Potential solution

This pattern likely indicates overamplification and the formation of secondary PCR products. To reduce the occurrence of these higher-molecular-weight bands, reduce the number of cycles used in the long-primer test or final PCR (Steps 43, 50).

### Problem 4

Libraries are detected after long-primer test or final PCR in the UV– and UV+ non-immune IgG or no-antibody control samples (related to Steps 43-47, 50, 54-55).

### Potential solution

This signal may arise from non-specific background. To reduce background, increase the stringency and number of wash steps (Steps 13, 15, 17), and carefully monitor protein–RNA complexes on the nitrocellulose membrane (Steps 24-25) in the UV– and UV+ non-immune IgG or no-antibody control lanes (see Methods S1). Alternatively, the signal may result from carryover contamination from previous experiments. In this case, thoroughly clean the workspace and maintain strict physical separation of pre- and post-PCR work areas to minimize cross-contamination.

### Problem 5

Low rate of uniquely mapping reads to the reference genome (related to Step 70).

### Potential solution

Poor unique mapping rates may result from experimental problems, such as RNA over-digestion, inefficient removal of adapter dimers or other contamination. Inspect the racoon_clip report to find out about potential problems and repeat the experiment if necessary. Moreover, a high content of multimapping reads may originate from rRNA or repetitive sequences which are often contaminants but can also be genuine RNA targets, depending on the RBP of interest. If the latter is anticipated, we suggest using dedicated approaches to capture rRNA or repeat element binding.

### Problem 6

High fraction of reads lost during duplicate removal (related to Step 70).

### Potential solution

A high duplication rate indicates a low library complexity which may originate from insufficient amounts of immunopurified RNA. This is usually reflected in an increased number of PCR cycles required during library preparation (see above).

### Problem 7

The racoon_clip pipeline breaks during the peak calling step with PureCLIP (related to Step 68).

### Potential solution

This usually occurs if RAM limits are reached. Restrict the training step of PureCLIP to selected chromosomes (see above) and/or provide additional RAM.

### Problem 8

racoon_clip fails at the start of the run (related to Step 68).

### Potential solution

This often points to either a problem with mamba or errors in the config file. Double-check for typos in the file names and paths. If problems persist, post an issue on the GitHub page of racoon_clip.

## Resource availability

### Lead contact

Further information and requests for resources and reagents should be directed to and will be fulfilled by the lead contact, Michaela Müller-McNicoll (mueller-mcnicoll@bio.uni-frankfurt.de).

### Technical contact

Technical questions on executing this protocol should be directed to and will be answered by the technical contacts, Vladimir Despic (despic@bio.uni-frankfurt.de) (experimental) and Melina Klostermann (melina.klostermann@uni-wuerzburg.de) (bioinformatic analysis).

### Materials availability

This study did not produce new materials, reagents, or cell lines.

### Data and code availability

• The U2AF2 iCLIP3 data generated in this study have been deposited at NCBI Gene Expression Omnibus (GEO) and will be publicly available under the accession number GEO: GSE325775. The U2AF2 iCLIP2 data used for comparison are available under the accession numbers GEO: GSM6793346 and GSM6793347.
• Scripts and configuration files used for processing the U2AF2 iCLIP3 data in this publication are available on GitHub (https://github.com/ZarnackGroup/Despic_et_al_2026) and Zenodo (https://doi.org/10.5281/zenodo.18743095, https://doi.org/10.5281/zenodo.20412265).

## Acknowledgments

This research was supported by the 10.13039/501100001659German Research Foundation (Deutsche Forschungsgemeinschaft, DFG) through grants ZA 881/6-1 (to K.Z.), TRR 267 (project no. 403584255, TP A03, to M.M.-M. and K.Z.), SFB 1551 (project no. 464588647, to J.K.), the excellence cluster Cardiopulmonary Institute (10.13039/501100021703CPI) EXC 2026/2 (project no. 390649896, to M.M.-M. and K.Z.), the excellence cluster SCALE EXC 3094/1 (project no. 533751785, to M.M.-M.), and the Cluster for Nucleic Acid Sciences and Technologies – NUCLEATE EXC 3113/1 (project no. 533767322, to J.K. and K.Z.), as well as by 10.13039/100004410EMBO (pc25/22, to Z.K., J.K. and M.M.-M.).

We gratefully acknowledge the Genomics Core Facility at Institute of Molecular Biology (IMB) Mainz, for sequencing the U2AF2 iCLIP3 libraries. We would like to thank Miona Ćorović, Elias Bechara, Rosario Avolio, and all EMBO iCLIP course participants for successfully testing the experimental iCLIP3 protocol; Annika Ladwig and Sarah Wolf for testing the data analysis protocol; and all members of the Müller-McNicoll, König and Zarnack labs for discussion.

## Author contributions

V.D., J.K., and M.M.-M. conceived the project. V.D. developed the experimental protocol, with contributions from A.O. and M.M. V.D. performed the experiments. M.K. performed the bioinformatics data analysis. V.D. and M.K. prepared the figures. A.B. developed the initial pipeline for processing iCLIP3 sequencing reads. A.O. performed the U2AF2 iCLIP3 experiment in the König lab. M.M. developed the Shiny app for library multiplexing with long primers with UDIs. V.D. wrote the experimental protocols and M.K. wrote the bioinformatics protocols, with input and edits from all co-authors. K.Z., J.K., and M.M.-M. supervised the project.

## Declaration of interests

The authors declare no competing interests.

## Supplemental information

Document S1. Methods S1 and S2 and Figures S1–S5

Data S1. Full processing report for U2AF2 iCLIP3 samples

Data S2. BindingSiteFinder example codes

Data S3. BindingSiteFinder analysis plots
