# Supplementary material for: Isotope-free mapping of protein-RNA interactions at single-nucleotide resolution by iCLIP3
Source: STAR Protoc. 2026 Jul 17;7(3):104704. doi: 10.1016/j.xpro.2026.104704 (PMC13400664; doi:10.1016/j.xpro.2026.104704)
Supplement: Document S1. Methods S1 and S2 and Figures S1–S5 [file mmc1.pdf]

Methods S1: Protocol for RNA binding assay and optimization of RNA fragmentation, related to Step 9.

The number of annotated RBPs has expanded to over 1,500; however, many lack canonical RNA-binding domains and have not been validated as direct RNA binders [1]. For all proteins of interest, and particularly for non-canonical or unvalidated RBPs, this step-by-step protocol must be executed prior to the main iCLIP3 library preparation. This protocol serves to confirm direct protein-RNA interactions, inspect the purity of isolated protein-RNA complexes, and determine the appropriate RNase I dilution required to generate RNA fragments within the optimal size range required for iCLIP3 library preparation.

KEY RESOURCES TABLE

REAGENT or RESOURCE	SOURCE	IDENTIFIER
Antibodies		
Anti-U2AF^{65} antibody, Mouse monoclonal (clone MC3), 5 µg per IP	Sigma-Aldrich	Cat#U4758
Anti-GFP antibody, Goat (15 µg/µL), 5 µg per IP	MPI-CBG	N/A
Chemicals, peptides, and recombinant proteins		
DMEM, high glucose, GlutaMAX, pyruvate	Thermo Fisher Scientific	Cat#10569010
Fetal bovine serum, Value	Thermo Fisher Scientific	Cat#A5256701
Penicillin-Streptomycin (10,000 U/mL)	Thermo Fisher Scientific	Cat#15140122
UltraPure DNase/RNase-Free Distilled Water	Thermo Fisher Scientific	Cat#10977035
UltraPure 1 M Tris-HCl, pH 7.5	Thermo Fisher Scientific	Cat#15567027
NaCl (5M), RNase-free	Thermo Fisher Scientific	Cat#AM9759
Sodium deoxycholate	Sigma-Aldrich	Cat#D6750-100G
Igepal CA-630	Sigma-Aldrich	Cat# I8896-50ML
Tween 20	Sigma-Aldrich	Cat# P9416-50ML
UltraPure SDS solution, 10%	Thermo Fisher Scientific	Cat#15553027
$MgCl_2$ (1M)	Thermo Fisher Scientific	Cat#AM9530G
EDTA (0.5 M), pH 8.0, RNase-free	Thermo Fisher Scientific	Cat#AM9260G
1M Tris-HCl, pH 6.5	Sigma-Aldrich	Cat#20-160
DL-Dithiothreitol solution (1 M in H_20)	Sigma-Aldrich	Cat#646563-10X.5ML
cOmplete, Mini, EDTA-free Protease Inhibitor Cocktail	Sigma-Aldrich	Cat#11836170001

Ambion RNase I, cloned, 100 U/µL	Thermo Fisher Scientific	Cat#AM2294
TURBO DNase (2 U/µL)	Thermo Fisher Scientific	Cat#AM2238
Recombinant RNasin Ribonuclease Inhibitor	Promega	Cat#N2511
T4 Polynucleotide kinase (T4 PNK)	NEB	Cat#M0201S
T4 RNA Ligase 1 (ssRNA Ligase), High Concentration	NEB	Cat#M0437M
10X T4 RNA Ligase Reaction buffer	NEB	Component of Cat#M0437M
50% Polyethylene Glycol (PEG) 8000	NEB	Component of Cat#M0437M
100 mM ATP	NEB	Component of Cat#M0437M
pCp-IR750 (1 mM)	Jena Bioscience	Cat#NU-1706-IR750
NuPAGE 4%–12% Bis-Tris Mini Protein Gels	Thermo Fisher Scientific	Cat#NP0322BOX
NuPAGE MOPS SDS Running Buffer (20X)	Thermo Fisher Scientific	Cat#NP0001
NuPAGE Transfer Buffer (20X)	Thermo Fisher Scientific	Cat#NP00061
NuPAGE LDS Sample Buffer (4X)	Thermo Fisher Scientific	Cat#NP0007
PageRuler Prestained Protein Ladder	Thermo Fisher Scientific	Cat#26616
Proteinase K, recombinant, PCR Grade (20 mg/mL)	Sigma-Aldrich	Cat#3115828001
Phenylmethylsulfonyl Fluoride (PMSF)	Cell Signaling Technology	Cat#8553S
Methanol	Carl Roth	Cat#0082.1
Ethanol	Sigma-Aldrich	Cat#32205-1L-M
2-Propanol (Isopropanol)	Sigma-Aldrich	Cat#190764
Novex TBE-Urea Sample Buffer (2X)	Thermo Fisher Scientific	Cat#LC6876
Low Range ssRNA Ladder	NEB	Cat#N0364S
TBE (10X), RNase-free	Thermo Fisher Scientific	Cat#AM9863
Novex TBE-Urea Gels, 10%	Thermo Fisher Scientific	Cat#EC68752BOX
SYBR Gold Nucleic Acid Gel Stain (10,000X)	Thermo Fisher Scientific	Cat#S11494
Critical commercial assays		
Dynabeads Protein G for Immunoprecipitation	Thermo Fisher Scientific	Cat#10003D
RNA Clean & Concentrator-5 Kit	Zymo Research	Cat#R1015
Experimental models: Cell lines		
Human HeLa cells	This study	N/A

Mouse embryonic P19 cells	Sigma-Aldrich	Cat#95102107-1VL
Other		
BioLite Cell Culture Treated Dishes, 100 mm	Thermo Fisher Scientific	Cat#130182
BIO-LINK BLX-254 UV Crosslinker	Vilber	N/A
Branson digital sonifier 250 with 1/8” tip	Emerson	N/A
DynaMag-2 Magnet magnetic rack (1.5 ml tubes)	Thermo Fisher Scientific	Cat#12321D
Proteus Clarification Mini Spin Columns	SERVA Electrophoresis	Cat#42225.01
XCell SureLock Mino-Cell and XCell II Blot Module	Thermo Fisher Scientific	Cat#EI0002
Whatman 3MM Chromatography Paper	GE Healthcare	Cat#3030917
Amersham Protran 0.45 µm Nitrocellulose Membrane	Cytiva	Cat#10600002
Swann-Morton Stainless Surgical Scalpels	Fisher Scientific	Cat#11728353
PowerPac Basic Power Supply	Bio-Rad	Cat#1645050
ChemiDoc MP Imaging System	Bio-Rad	N/A
Thermomixer	N/A	N/A
Falcon 15 mL Conical Centrifuge Tubes	Corning	Cat#CLS352096
SafeSeal Reaction Tube, 1.5 mL, DNA Low Binding	SARSTEDT	Cat#72.706.700
Nonstick, RNase-free Microfuge Tubes, 1.5 mL	Thermo Fisher Scientific	Cat#AM12450
ROTILABO PES Syringe Filters, 0.22 µm	Carl Roth	Cat#P668.1
Omnifix 50 mL Disposable Syringes	Carl Roth	Cat#T552.2
Omnifix 10 mL Disposable Syringes	Carl Roth	Cat#C542.1

MATERIALS and EQUIPMENT

Prepare all buffers in nuclease-free water.

Lysis buffer

Filter the buffer through 0.22 µm filter and store at 4ºC for up to 1 month.

Reagent	Final concentration	Volume (mL)
1 M Tris-HCl, pH 7.5	50 mM	2
5 M NaCl	100 mM	0.8
10% Igepal CA-630 (v/v)	1%	4
5% Na-deoxycholate (w/v)	0.5%	4
10% SDS	0.1%	0.4
Nuclease-free water	N/A	28.8
**Total**	N/A	**40**

High Salt buffer

Filter the buffer through 0.22 µm filter and store at 4ºC for up to 1 month.

Reagent	Final concentration	Volume (mL)
1 M Tris-HCl, pH 7.5	50 mM	2
5 M NaCl	1 M	8
10% Igepal CA-630 (v/v)	1%	4
5% Na-deoxycholate (w/v)	0.5%	4
10% SDS	0.1%	0.4
0.5 M EDTA, pH 8.0	1 mM	0.08
Nuclease-free water	N/A	21.52
**Total**	N/A	**40**

PNK Wash buffer

Filter the buffer through 0.22 µm filter and store at 4ºC for up to 1 month.

Reagent	Final concentration	Volume (mL)
1 M Tris-HCl, pH 7.5	20 mM	0.8
1 M $MgCl_2$	10 mM	0.4
10% Tween-20 (v/v)	0.2%	0.8
Nuclease-free water	N/A	38
**Total**	N/A	**40**

Last Wash buffer

Filter the buffer through 0.22 µm filter and store at 4ºC for up to 1 month.

Reagent	Final concentration	Volume (mL)
1 M Tris-HCl, pH 7.5	20 mM	0.4
5 M NaCl	100 mM	0.4
10% Tween-20 (v/v)	0.2%	0.4
0.5 M EDTA, pH 8.0	2 mM	0.08

Nuclease-free water	N/A	18.72
**Total**	N/A	**20**

5X PNK buffer pH 6.5

Store aliquots of the buffer at -20ºC. Do not thaw and freeze again.

Reagent	Final concentration	Volume (μL)
1 M Tris-HCl, pH 6.5	350 mM	70
250 mM $MgCl_2$	50 mM	40
100 mM DTT	5 mM	10
Nuclease-free water	N/A	80
**Total**	N/A	**200**

Proteinase K buffer

Filter the buffer through 0.22 μm filter and store at 4ºC for up to 1 month.

Reagent	Final concentration	Volume (mL)
1 M Tris-HCl, pH 7.5	100 mM	1
5 M NaCl	50 mM	0.1
0.5 M EDTA pH 8.0	10 mM	0.2
10% SDS (v/v)	0.2%	0.2
Nuclease-free water	N/A	8.5
**Total**	N/A	**10**

STEP-BY-STEP METHOD DETAILS

Cell culture and UV irradiation

Timing: variable, 2 days for sample preparation

This section describes how to crosslink RBPs to their RNA targets by briefly exposing cells to 254 nm UV light.

1. Seed cells for the experiment.

a. Grow HeLa cells in high glucose, pyruvate 1X DMEM + GlutaMAX medium supplemented with 10% heat-inactivated FBS and 1X Penicillin-Streptomycin (Pen-Strep).
b. One day before the experiment, seed HeLa cells onto two 100-mm dishes to achieve ~85% confluency the following day.

2. Wash cells with PBS.
   a. Aspirate the culture medium from each dish.
   b. Gently wash the cells with 6 mL ice-cold PBS.
   c. Remove the PBS and add 6 mL fresh, ice-cold PBS to each dish.

Note: Consider adding protease inhibitors to PBS if the cell line of interest is known to show significant protease activity during cell harvesting.

3. Irradiate cells with 254 nm UV light.
   a. Place one cell dish containing PBS on an ice tray covered with a thin layer of water (**Figure S1**).
   b. Remove the lid and irradiate the cells once with 254-nm UV light at 150 mJ/cm^2.
   c. Keep the second dish on ice without UV exposure as a non-irradiated control.

4. Harvest cells.
   a. Gently scrape the cells in PBS using cell lifters.
   b. Transfer the cell suspensions into 15 mL Falcon tubes labeled UV+ (UV-irradiated) and UV– (non-UV-irradiated).
   c. Pellet the cells by centrifugation at 300 x *g* for 5 min at 4ºC.

Note: If available, use a swinging-bucket rotor to ensure the cell pellets concentrate compactly at the bottom of the tubes.

   d. Carefully aspirate the PBS without disturbing the cell pellets.
   e. Snap-freeze the cell pellets on dry ice or in liquid nitrogen and store at -80ºC.

Pause point: Frozen cell pellets can be kept at -80ºC long term.

Antibody-bead coupling

Day 1 15 min + Timing: 1–2 h

This section describes how to couple the antibody for the target RBP to magnetic beads.

5. Couple the antibody to magnetic Protein A or Protein G Dynabeads.

Note: Verify the antibody's binding preference for Protein A or Protein G Dynabeads by consulting the manufacturer's recommendations.

   a. Aliquot 30 µL Protein A or G Dynabeads stock per sample into a 1.5 mL tube.

Note: For six samples, aliquot 189 µL beads (6.3x surplus required for this protocol).

b. Wash the beads twice with 750 µL Lysis buffer.
c. Resuspend the beads in 300 µL Lysis buffer and add 5 µg antibody per sample specific for the RBP of interest.

Note: For six samples, use 31.5 µg antibody in a total volume of 500 µL Lysis buffer. Lower amounts of antibody per sample may be used. All washes in the protocol are performed by pipetting unless otherwise stated.

d. Incubate the beads on a rotating wheel at 20ºC–24ºC for ≥1 h (until the lysates are ready for immunoprecipitation).

Cell lysis and protein quantification

Day 1 **Timing: 1 h**

This section describes how to lyse frozen cell pellets and quantify the total protein concentration in the resulting lysates.

6. Lyse cell pellets.
   a. Thaw frozen cell pellets on ice.
   b. Resuspend each cell pellet in 750 µL Lysis buffer supplemented with 1X Protease Inhibitors (1X PIs).
   c. Lyse the cells on ice for 10 min.
   d. **Optional:** Sonicate the lysate on ice with a Branson digital sonifier 250 at 10% power amplitude using 5 cycles of 5 s pulses with 10 s pauses between pulses.

Note: Sonication is recommended for nuclear proteins. If sonication is omitted, extend the lysis time to 20 min on ice. The sonication settings described here may not be fully applicable to another type of sonifier and may require optimization.

   e. Transfer the lysates to 1.5 mL tubes.
   f. Clarify the lysates by centrifugation at 16,000 x *g* for 10 min at 4ºC.
   g. Transfer the supernatants to fresh 1.5 mL tubes.

7. Determine the protein concentration in the cleared lysates using a BCA Protein Assay kit according to the manufacturer's instructions.

8. Prepare the lysates for RNA fragmentation.
   a. Dilute the UV– and UV+ lysates to a final protein concentration of 0.5 mg/mL using Lysis buffer supplemented with 1X PIs. Prepare at least 1.6 mL of UV– and UV+ lysates at 0.5 mg/mL to proceed.
   b. Label three 1.5 mL tubes per condition as H (High), M (Medium), and L (Low).
   c. Transfer 500 µL diluted lysate (250 µg protein) into each tube and keep the lysates on ice.

RNA fragmentation

Day 1 Timing: 20 min

This section outlines how to test the optimal RNA digestion conditions in the lysate using different RNase I dilutions to generate fragments within a 50 – 300 nt size range suitable for iCLIP3 library preparation and sequencing. Additionally, it describes how to use a high RNase I concentration to generate 4–20 nt RNA fragments to evaluate IP efficiency and purity.

9. Treat lysates with DNase.
   a. Add 2 µL **TURBO DNase** to each diluted lysate.
   b. Gently invert the tubes several times to mix.
   c. Briefly spin down the samples and return them to ice.
10. Fragment RNA in lysates with RNase I.
   a. Prepare different RNase I concentrations in nuclease-free water as follows:

RNase I	RNase I, 100 U/µL (µL)	Nuclease-free water (µL)
High (H, 20 U/µL)	6	24
Medium (M, 1 U/µL)	1	99
Low (L, 0.2 U/µL)	1	499

Note: HeLa and P19 cell lysates with total protein amount other than 250 µg and protein concentrations differing from 0.5 mg/mL may require adjusted RNase I concentrations to optimize RNA fragmentation. Cells with high endogenous RNase activity may also require distinct RNase I concentration adjustments.

   b. Add 10 µL of the appropriate **RNase I concentration** to each corresponding lysate (H, M, or L).
   c. Gently invert the tubes several times to mix, then briefly spin down.
   d. Immediately incubate the samples in a thermomixer at 37ºC for 3 min with shaking at 1,100 rpm.

Critical: Strictly adhere to a 3 min-long incubation for consistent results.

   e. Immediately place the samples on ice after incubation and keep it on ice for ≥ 3 min to stop RNA digestion.
   f. **Optional:** Load the samples onto Proteus mini clarification spin columns, centrifuge at 16,000 x *g* for 1 min at 4ºC and transfer the flow-throughs to new 1.5 mL tubes.

Immunoprecipitation

Day 1 Timing: 2.5 h

This section describes how to immunopurify the target RBP from RNase I-treated lysates using magnetic beads coupled with an RBP-specific antibody.

11. Clean up antibody-bead complexes.
    a. Briefly spin down the antibody–bead coupling mixture from Step 5d.
    b. Place the tube on a magnetic rack to collect the beads and discard the supernatant.
    c. Wash the beads twice with 750 µL Lysis buffer.
    d. Resuspend the beads for each sample in 100 µL Lysis buffer supplemented with 1X PIs.

Note: For six samples, resuspend the beads in 630 µL Lysis buffer supplemented with 1X PIs.

12. Immunoprecipitate the target RBP.
    a. Add 100 µL antibody-coupled beads from Step 11d to each RNase I-treated lysate from Step 10e (or Step 10f, if performed).
    b. Incubate the lysates with the beads on a rotating wheel for 2 h at 4ºC.

Note: Adjust the incubation time according to the optimized immunoprecipitation conditions for the RBP of interest.

13. Clean up the immunoprecipitation.
    a. Briefly spin down the samples and place them on a magnetic rack to collect the beads.
    b. Discard the supernatant.
    c. Wash the beads twice with 800 µL High Salt buffer. Incubate the second wash for 5 min on a rotating wheel at 4ºC. After incubation, briefly spin down the tubes to collect beads from the tube lids before proceeding.
    d. Wash the beads twice with 800 µL Lysis buffer. During the second Lysis buffer wash, transfer the beads to new 1.5 mL tubes.
    e. Wash beads once with 800 µL PNK Wash buffer.
    f. Perform a final wash with 300 µL PNK Wash buffer.
    g. Keep the beads on ice before proceeding to the next step.

Pause point: The beads can be kept in the PNK Wash buffer on ice for 1–2 h.

3′ RNA dephosphorylation

Day 1 Timing: 45 min

RNase I generates RNA fragments with 2′,3′-cyclic phosphates and 3′-phosphates, which require conversion to 3′-hydroxyl groups to enable efficient ligation of pCp-IR750 and DNA adapters. This section describes how to dephosphorylate crosslinked RNA fragments on beads using T4 polynucleotide kinase (PNK).

14. Dephosphorylate RNA 3’ ends.

    a. Prepare the RNA Dephosphorylation buffer, mix thoroughly by pipetting and keep on ice.

Reagent	Volume per reaction (µL)
Nuclease-free water	14.5
5X T4 PNK buffer (pH 6.5)	4
RNasin	0.5
**Total**	**19**

    b. Place the beads from Step 13g on a magnetic rack and remove the PNK Wash buffer.
    c. Briefly spin down the tubes, return them to the magnetic rack and remove any residual buffer using a P20 pipette.

Note: Process one sample at a time to avoid drying the beads.

    d. Resuspend the beads in 19 µL RNA Dephosphorylation buffer.
    e. Add 1 µL **T4 PNK** and mix thoroughly by pipetting.
    f. Incubate the samples for 20 min at 37ºC in a thermomixer with shaking at 1,200 rpm.

Note: Place the sample in the thermomixer immediately after assembling the reaction to prevent bead sedimentation.

15. Clean up 3′ RNA dephosphorylation.

    a. Add 100 µL PNK Wash buffer without resuspending the beads.
    b. Place the tubes on a magnetic rack to collect the beads and remove the PNK Wash buffer.
    c. Wash the beads twice with 800 µL High Salt buffer. Incubate the second wash for 2 min on a rotating wheel at 4ºC. After incubation, briefly spin down the tubes to collect beads from the tube lids before proceeding.
    d. Wash the beads with 800 µL Lysis buffer. Transfer the beads to new 1.5 mL tubes during this wash.
    e. Wash the beads once with 800 µL PNK Wash buffer.
    f. Perform a final wash with 300 µL PNK Wash buffer.

16. Thoroughly resuspend the beads by pipetting and divide them into two tubes as follows:
    - Tube 1: 270 µL (90% beads)
    - Tube 2: 30 µL (10% beads)

Pause point: Store the samples at 4ºC for ≤ 20 h.

3’ RNA labeling with pCp-IR750

Day 2 **Timing: 1.5 h**

This section describes how to ligate pCp-IR750 on beads to 10% of the immunopurified RNA fragments with T4 PNK-repaired 3′ ends. Labeling this subset of RNA fragments is sufficient to enable rapid and safe visualization of RBP–RNA complexes on a nitrocellulose membrane (**Figures S2A** and **S2B**).

Note: pCp-IR750 requires imaging using near-infrared Cy7 or IRDye 800CW channels. We do not recommend pCp-Cy5 due to high background signal on the membrane in the Cy5 imaging channel. We did not thoroughly evaluate other pCp-fluorophore conjugates.

17. Label RNA 3’ ends with pCp-IR750.

    a. Prepare the RNA Labeling buffer, mix thoroughly by pipetting and keep at 20ºC–24ºC.

Reagent	Volume per reaction (μL)
Nuclease-free water	7.75
10X RNA Ligase Reaction buffer	2
10 mM ATP	2
200 μM pCp-IR750	1
RNasin	0.25
50% PEG8000	6
**Total**	**19**

Note: Pipette the assembled labeling buffer slowly and thoroughly to ensure homogeneous distribution of all components. PEG8000 is highly viscous; avoid liquid retention in the pipette tip. Protect the buffer from light.

    b. Place Tube 2 (10% beads) from Step 16 on a magnetic rack to collect the beads and remove the PNK Wash buffer.
    c. Resuspend the beads in 19 μL RNA Labeling buffer.
    d. Add 1 μL **T4 RNA Ligase 1** (high concentration).
    a. Mix the reaction thoroughly by pipetting until the beads are evenly distributed. Pipette slowly to avoid bead retention in the pipette tip.
    e. Incubate the samples at 27ºC for 1 h in a thermomixer with shaking at 1,200 rpm.

Critical: Do not incubate the labeling reaction longer than 1 h to avoid protein labeling. Protect the samples from light using aluminum foil.

18. Clean up 3′ RNA labeling reaction.

    a. Add 100 μL PNK Wash buffer without resuspending the beads.

b. Place the samples on a magnetic rack to collect the beads and remove the PNK Wash buffer.
c. Wash the beads twice with 400 µL High Salt buffer.
d. Wash the beads with 400 µL Lysis buffer. Transfer the beads to new 1.5 mL tubes during this wash.
e. Wash the beads once with 400 µL PNK Wash buffer.
f. Perform a final wash with 100 µL PNK Wash buffer.

Elution of RBP–RNA complexes

Day 2 Timing: 20 min

This section describes how to combine, wash, and elute the non-labeled (90%) and pCp-IR750-labeled (10%) RBP–RNA complexes from the beads. Labeling a subset of RNA fragments with pCp-IR750 allows direct visualization of immunopurified RBP–RNA complexes on a nitrocellulose membrane. The remaining, non-labeled RNA (90%) is subsequently labeled with pCp-IR750 after RNA isolation from the nitrocellulose membrane.

19. Combine beads from Tube 1 and Tube 2.

a. Transfer the beads from Tube 2 (100 µL) from Step 18f into Tube1 (270 µL) from Step 16.
b. Wash the combined beads with 300 µL Last Wash buffer.
c. Discard the supernatant.

20. Elute RBP–RNA complexes.

a. Collect the beads on a magnetic rack and discard the Last Wash buffer.
b. Resuspend the beads for each sample in 20 µL 1X LDS NuPAGE Loading buffer supplemented with 50 mM DTT.
c. Incubate the beads in a thermomixer at 70ºC for 5 min with shaking at 1,100 rpm.
d. Briefly spin down the samples, collect the beads on a magnetic rack and transfer the eluates to new 1.5 mL tubes.

SDS-PAGE and nitrocellulose transfer of the RBP–RNA complexes

Day 2 Timing: 3 h

This section describes how to resolve the eluted RBP-RNA complexes using denaturing SDS-PAGE and how to transfer them onto a nitrocellulose membrane. Because proteins efficiently bind nitrocellulose, directly crosslinked RNA remains attached to the membrane via the RBP, whereas background or non-crosslinked RNA does not stably bind.

21. Resolve RBP-RNA complexes using SDS-PAGE.

a. Prepare 0.5 L 1X NuPAGE MOPS SDS Running buffer using nuclease-free water.

b. Prepare the Prestained Protein Ruler (protein ladder) dilution for a single gel lane by mixing 2.5 μL protein ladder stock solution with 17.5 μL 1X LDS NuPAGE Loading buffer.

Note: For this experiment, prepare PreStained Protein Ruler dilution for 6 gel lanes by mixing 15 μL protein ladder stock solution and 105 μL 1X LDS NuPAGE Loading buffer.

c. Assemble a 12-well 4-12% Bis-Tris SDS gel in the XCell SureLock module according to the manufacturer's instructions.
d. Load 20 μL of each sample in the following order:

PL	UV– H	UV– M	UV– L	PL	UV+ H	PL	UV+ M	PL	UV+ L	PL	
1	2	3	4	5	6	7	8	9	10	11	12

where PL: Protein Ladder, UV–: non-irradiated, UV+: UV-irradiated, H: high RNase I, M: medium RNase I, L: low RNase I.

e. Run the gel at 180 V for 50-60 min.

22. Transfer RBP–RNA complexes to a nitrocellulose membrane.

a. Prepare 1X NuPAGE Transfer buffer from 20X stock using nuclease-free water and 20% (vol/vol) methanol or ethanol.
b. Cut the nitrocellulose membrane and four pieces of Whatman paper.
c. Carefully open the gel cassette.
d. Assemble the transfer sandwich using the XCell II module according to the manufacturer's instructions.

Note: Pre-wet the sponges and remove any air bubbles using a roller.

e. Perform the transfer for 1.5 h at 30 V.

Note: For proteins smaller than 50 kDa, 1 h at 30 V is usually sufficient. For large proteins (>180 kDa), longer transfer times may be required.

Membrane imaging

Day 2 Timing: 15 min

This section describes how to directly visualize the pCp-IR750-labeled, immunopurified RBP-RNA complexes on the nitrocellulose membrane.

23. Visualize immunopurified RBP–RNA complexes.

a. Disassemble the transfer sandwich.

b. Using forceps, place the membrane in a clean plastic tray containing 1X PBS.
c. Place the membrane onto a thin, transparent plastic foil and insert it into the imager.

Note: Do not place the membrane directly onto the transilluminator plate to avoid cross-contamination with nucleic acids.

d. Image the membrane using the Cy7 or IRDye 800CW channel to visualize the RBP–RNA complexes.
e. Image the membrane in the colorimetric channel to visualize the protein ladder.
f. Merge the two images into a single composite image and save.
g. Place the membrane back into the plastic tray containing 1X PBS.

24. Inspect the image and identify the region of the membrane showing RNA signal that corresponds to the size range of the immunopurified RBP–RNA complexes (**Figures S2A** and **S2B**).

Note: PageRuler prestained protein ladder bands exhibit different apparent molecular weights when resolved on a Bis-Tris gel using MOPS SDS running buffer compared to their nominal sizes.

Elution of RNA from the membrane with Proteinase K treatment

Day 2 **Timing: 1 h**

This section describes how to excise nitrocellulose regions containing RBP-RNA complexes from UV+ samples and treat them with Proteinase K. As a non-specific protease, Proteinase K digests the RBP to release both pCp-IR750-labeled (10%) and non-labeled (90%) RNA from the membrane.

25. Cut out the membrane part containing RBP-RNA complexes.

a. Place the membrane on a transparent, thick foil.
b. Using sterile scalpels, cut the sample lane between two proteins ladders corresponding to the size of the immunopurified RBP–RNA complexes in UV+ samples (**Figure S2B**).
c. Transfer the excised membrane region to a 6 or 10-cm dish.
d. Shred the membrane further into smaller pieces.
e. Prepare 1.5 mL tubes with 150 µL Proteinase K buffer.
f. Using needle tips, transfer the membrane pieces into the 1.5 mL tubes containing the Proteinase K buffer.

Note: Ensure that all membrane pieces are fully submerged in the Proteinase K buffer.

26. Digest RBP with Proteinase K.

a. Add 10 µL **Proteinase K** (20 mg/mL) to each sample.

b. Incubate the samples in a thermomixer at 37ºC for 20 min with shaking at 1,000 rpm.
c. Continue the incubation in a thermomixer at 50ºC for 20 min with shaking at 1,000 rpm.
d. Briefly spin down the samples and transfer the supernatants (~155 µL) into new 1.5 mL tubes.

RNA isolation

Day 2 **Timing: 15 min**

This section describes how to purify the RNA released after Proteinase K treatment using a silica column-based approach.

27. Inactivate Proteinase K.
    a. Add 45 µL nuclease-free water to the sample from Step 26d.
    b. Add 1 µL 0.5 M phenylmethylsulfonylfluoride (PMSF) and briefly vortex.
    c. Briefly spin down the samples and incubate at 20ºC–24ºC for ≥ 3 min.
28. Isolate RNA using RNA Clean and Concentrator-5 kit.
    a. Add 400 µL (2X volume) RNA Binding buffer and briefly vortex.
    b. Briefly spin the samples, add 700 µL 100% Isopropanol (3.5X starting volume) and briefly vortex.
    c. Incubate the samples for 15 min at 20ºC–24ºC on a rotating wheel.
    d. Briefly spin down the samples, transfer 650 µL of each sample into the Zymo-Spin IC column and centrifuge at 5,000 x g for 30 s at 20ºC–24ºC.
    e. Discard the flow-through from the collection tube and add the remaining sample to the same column.
    f. Centrifuge at 5,000 x g for 30 s at 20ºC–24ºC and discard the flow-through.
    g. Wash the columns with 400 µL RNA Prep buffer, centrifuge at 5,000 x g for 30 s at 20ºC–24ºC and discard the flow-through.
    h. Wash the columns with 500 µL RNA Wash buffer (with ethanol added), centrifuge at 5,000 x g for 30 s at 20ºC–24ºC and discard the flow-through.
    i. Wash the columns with 250 µL RNA Wash buffer (with ethanol added), centrifuge at 9,000 x g for 30 s at 20ºC–24ºC and discard the flow-through.
    j. Centrifuge at 9,000 x g for additional 2 min at 20ºC–24ºC and transfer the columns to clean 1.5 mL tubes, being careful to avoid contact between the wash buffer and the columns.
    k. Add 9.2 µL nuclease-free water to each column, incubate at 37ºC for 2 min and then centrifuge at 15,000 x g for 1 min at 20ºC–24ºC.
    l. Discard the column and freeze the eluted RNA at -80ºC.

Pause point: RNA can be stored at -80ºC until the next day.

RNA labeling

Day 2 Timing: 2.5 h

This section describes how to ligate pCp-IR750 to 90% of the previously unlabeled, immunopurified RNA. Labeling this fraction after RNA isolation preserves the IR750 signal and minimizes photobleaching.

29. Label RNA 3' ends with pCp-IR750.

    a. Set up the RNA labeling reaction as follows:

Reagent	Volume per reaction (µL)
Isolated RNA	8.8
10X RNA Ligase Reaction buffer	2
20 mM ATP	1
200 µM pCp-IR750	1
RNasin	0.2
DMSO	2
50% PEG8000	4
T4 RNA ligase 1 (high concentration)	1
**Total**	**20**

    b. Mix thoroughly by pipetting and incubate the ligation reaction in a thermomixer at 27ºC for 2 h with shaking at 1,000 rpm.

30. Isolate pCp-IR750-labeled RNA using RNA Clean and Concentrator-5 kit.

    a. Add 60 µL nuclease-free water to the ligation reaction.
    b. Add 160 µL RNA Binding buffer and briefly vortex.
    c. Briefly spin the samples, then add 280 µL isopropanol and briefly vortex.
    d. Incubate the samples on a rotating wheel at 20ºC–24ºC for 15 min, protected from light.
    e. Apply the samples to the Zymo-Spin IC columns, centrifuge at 5,000 x g for 30 s at 20ºC–24ºC and discard the flow-through.
    f. Wash the columns with 400 µL RNA Prep buffer, centrifuge at 5,000 x g for 30 s at 20ºC–24ºC and discard the flow-through.
    g. Wash the columns with 500 µL RNA Wash buffer (with ethanol added), centrifuge at 5,000 x g for 30 s at 20ºC–24ºC and discard the flow-through.
    h. Wash the columns with 250 µL RNA Wash buffer (with ethanol added), centrifuge at 9,000 x g for 30 s at 20ºC–24ºC and discard the flow-through.

i. Centrifuge at 9,000 x g for additional 2 min at 20ºC–24ºC and transfer the columns to clean microcentrifuge tubes, being careful to avoid contact between the wash buffer and the columns.
j. Add 7.2 µL nuclease-free water to each column, incubate at 37ºC for 2 min and then centrifuge at 15,000 x g for 1 min at 20ºC–24ºC.
k. Discard the columns and keep the eluted RNA on ice until further use.

Denaturing RNA gel

Day 3 Timing: 1.5 h

This section describes how to resolve pCp-IR750-labeled RNA on a denaturing 10% TBE-urea gel to visualize the immunopurified RNA fragments and determination their size range.

31. Resolve pCp-IR750-labeled RNA on a denaturing RNA gel.

a. Prepare 7 µL Low Range Single-Stranded RNA (ssRNA) Ladder diluted 1:5 in nuclease-free water.
b. Add 5 µL 2X TBE–Urea Sample buffer to each RNA sample from Step 30k and to the diluted ssRNA ladder.
c. Heat the samples and the ssRNA ladder at 65ºC for 5 min prior to loading.
d. Immediately place the samples and the ssRNA ladder on ice.
e. Assemble a 10% TBE-urea gel in the XCell SureLock module and fill the chamber with 1X TBE Running buffer according to the manufacturer's instructions.
f. Use a P1000 pipette tip and flush any precipitated urea from the wells.
g. Load 12 µL of each sample and the ssRNA ladder into the wells.
h. Run the gel for 60 min at 200 V.
i. Open the gel cassette and carefully remove the gel.
j. Stain the gel for 5 min in 30 mL 1X TBE buffer containing 3 µL SYBR Gold.
k. Visualize the pCp-IR750-labeled RNA using the Cy7 or IRDye 800CW channel.
l. Continue imaging the gel using the SYBR Gold channel to visualize the ssRNA ladder.
m. Merge images from both channels and determine the size range of immunopurified RNA fragments based on the ssRNA ladder migration (**Figure S2C**).

EXPECTED OUTCOMES

This protocol allows researchers to qualitatively assess whether the RBP of interest directly binds RNA *in vivo*, inspect the purity of isolated RBP-RNA complexes, and optimize RNase I-based RNA fragmentation. RNA labeling of the sample with high RNase I concentration should produce a strong pCp-IR750 signal in the UV+ sample, appearing as a single band corresponding to the molecular weight of the RBP of interest, whereas the UV– sample should show minimal or ideally no near-infrared RNA signal. In UV+ samples treated with

medium and low RNase I concentrations, the distinct RBP–RNA complex band gradually disperses into an upward smear, while the respective UV– samples remain devoid of near-infrared RNA signal (**Figures S2A** and **S2B,** see Main Protocol **Figure 3C**). This shift in signal confirms that the visualized complexes contain RNA. For HeLa and mouse P19 cell lines, the optimal RNase I dilution lies within the 1:100–1:150 range in lysates containing 250 µg total protein at 0.5 mg/mL protein concentration, resulting in the majority of immunopurified RNA fragments in 50–300 nt size range (**Figures S2B** and **S2C**). RNase I dilutions should be adjusted for lysates with substantially different protein or RNA amounts or concentrations. During RNase I optimization, all samples should be adjusted to match the sample with the lowest protein amount and concentration.

TROUBLESHOOTING

Problem 1

No IP-grade antibody is available for the RBP of interest.

Potential solution

Use CRISPR-based technologies to insert a protein tag (e.g., GFP, HA) at the N- or C-terminus of the RBP. Then, perform IPs using commercially available antibodies or nanobodies specific to the inserted tag. Heterozygous tagging might be sufficient for an iCLIP3 experiment. Tagged proteins can also be transiently expressed from a transfected plasmid. However, transient expression should be carefully titrated as protein overexpression can lead to non-specific RNA binding of the protein and should hence be avoided.

Problem 2

Infrared RNA signal appears weak on the nitrocellulose membrane, even after long exposure (≥ 3 min) in UV+ samples, while UV– samples show no visible RNA signal (related to Steps 23-24).

Potential solution

The protein of interest may still bind RNA specifically, but the starting material could be limiting due to low protein expression or low RNA binding capacity. Increase the amount of starting material, such as the number of cells. Importantly, optimize RNase I treatment on the resulting lysates with higher protein amount and concentration or use multiple 250 µg lysate tubes at 0.5 mg/mL for RNase I treatment and combine them prior to immunoprecipitation (Steps 8-10). In some cases, increasing the amount of antibody or using an alternative antibody can help (Step 5).

Problem 3

Near-infrared RNA signal appears after longer exposure and looks the same in UV– and UV+ samples at high RNase I concentration. A distinct band does not disperse into an upward smear at medium and low RNase I concentrations (related to Steps 23-24).

Potential solution

The protein of interest may interact weakly, transiently or not at all with RNA nucleobases. Consider increasing the UV energy dose (Step 3).

Problem 4

Multiple near-infrared bands are observed on the nitrocellulose membrane in the UV+ sample with high RNase I concentration (related to Steps 23-24).

Potential solution

Increase the stringency of the washing steps following the IP (Steps 13, 15, 18). Refer to the following publication for the recommended buffer recipes [2].

Problem 5

Near-infrared RNA signal is observed in the control samples where the specific antibody was omitted during the immunoprecipitation (related to Steps 23-24).

Potential solution

Increase the stringency of the washing steps following the IP (Steps 13, 15, 18). Refer to the following publication for the recommended buffer recipes [2].

Methods S2: Protocol for identification of binding sites using BindingSiteFinder, related to Step 61.

Timing: 1-2 days

After extracting the crosslink events with racoon_clip, discrete binding sites have to be defined among the broad background across transcripts. For this purpose, we use the R/Bioconductor package BindingSiteFinder [3] , which refines binding site boundaries and supports replicate-aware filtering to improve robustness.

Note: In most cases, the binding sites of the RNA-binding protein can be distinguished from background because they typically display higher signal and a bell-shaped accumulation of crosslink events [4,5].

Note: The package BindingSiteFinder provides a wrapper function that executes the complete workflow with automatic parameter estimation when no arguments are supplied. In addition, multiple parameters can be specified to refine binding site definition. We recommend testing different parameter settings and evaluating their impact by visualizing the resulting binding sites together with the racoon_clip crosslink tracks in a genome browser.

KEY RESOURCES TABLE

REAGENT or RESOURCE	SOURCE	IDENTIFIER
Deposited data		
Genome assembly (e.g., GRCm38 / hg38)	GENCODE	https://www.gencodegenes.org
Gene annotation (e.g., GENCODE, release 49)	GENCODE	https://www.gencodegenes.org
(optional) rRNA sequences	NCBI/Refseq	https://www.ncbi.nlm.nih.gov/nuccore/
(optional) Genomes of potential contaminating organisms	ENSEMBL	https://www.ensembl.org/index.html
U2AF2 iCLIP3	This study	GEO: GSE325775
U2AF2 iCLIP2 rep1	Ebersberger et al. [6]	GEO: GSM6793346
U2AF2 iCLIP2 rep2	Ebersberger et al. [6]	GEO: GSM6793347
Software and algorithms		
R (version ≥ 4.0.0)	R Foundation	https://www.biozentrum.uni-wuerzburg.de/brb/resources/

RStudio	Posit	https://github.com/ZarnackGroup/racoon_clip
Bioconductor	Huber et al. [7]	https://www.bioconductor.org/
BindingSiteFinder	doi:10.1016/j.ymeth.2019.11.008	https://www.bioconductor.org/packages/release/bioc/html/BindingSiteFinder.html

STEP-BY-STEP PROTOCOL

Setup of R and R packages

During this step, all R packages are installed that are required for running the BindingSiteFinder.

1. Install R software and the necessary R packages.

Note: The definition of binding sites in R can be performed on a local computer.

   a. Install the latest version of R by following the installation guide.

Optional: Install the latest version of RStudio by following the installation guide.

   b. Open R or RStudio and install the required R packages.

```
R (all code blocks from here)
if (!require("BiocManager", quietly = TRUE))
    install.packages("BiocManager")

install.packages(“knitr”)# optional for rendering reports
install.packages(“tidyverse”)
install.packages(“purrr”)
BiocManager::install(“rtracklayer”)
BiocManager::install(“BindingSiteFinder”)
BiocManager::install(“GenomicRanges”)
BiocManager::install(“GenomeInfoDb”)
BiocManager::install(“txdbmaker”)
```

2. Open a new file (e.g., in R, RMD or QMD format) to document the code for the analysis steps.

Note: All following steps are provided in the example script in **Data S2**.

3. Load the libraries.

```
# ----------------------
# load libraries
# ----------------------
library(knitr)
library(purrr)
library(rtracklayer)
library(tidyverse)
library(BindingSiteFinder)
library(GenomicRanges)
library(GenomeInfoDb)
library(GenomicFeatures)
library(txdbmaker)
```

Automatic binding site definition

This step will run BindingSiteFinder in its default mode to define binding sites with automatic parameter estimation.

4. Define an output folder.

```
# set output folder
out <- "<path/to/your/output_folder>"
```

5. Extract genes and gene regions from the annotation file as input for BindingSiteFinder.

Note: Execute this step only once to extract the gene and transcript region coordinates. Save them as RDS files and load them directly in subsequent analyses to reduce computation time.

   a. Import annotation file (in GTF format).

Note: Use the same annotation file that was used for racoon_clip.

```
# ----------------------
# prepare annotation
# ----------------------

# GTF annotation file (downloaded for example from GENCODE)
annoFile <- "path/to/annotation.gtf.gz"

# make annotation database from GTF file
annoDb = txdbmaker::makeTxDbFromGFF(file = annoFile, format =
"gtf")
annoInfo = rtracklayer::import(annoFile, format = "gtf")
```

b. Extract gene coordinates including metadata and save as RDS file.

```
# get genes as GRanges
gns = genes(annoDb)
idx = match(gns$gene_id, annoInfo$gene_id)
meta = cbind(elementMetadata(gns),
             elementMetadata(annoInfo)[idx,])
meta = meta[,!duplicated(colnames(meta))]
elementMetadata(gns) = meta

out_gns <- paste0(out, "gns.rds")
saveRDS(gns, out_gns)
```

c. Extract transcript regions and save as RDS file.

```
# get transcript regions as Granges
cdseq = cds(annoDb)
intrns = unlist(intronsByTranscript(annoDb))
utrs3 = unlist(threeUTRsByTranscript(annoDb))
utrs5 = unlist(fiveUTRsByTranscript(annoDb))
regions = GRangesList(CDS = cdseq, INTRON = intrns, UTR3 = utrs3,
UTR5 = utrs5)

out_regions <- paste0(out, "regions.rds")
saveRDS(regions, out_regions)
```

6. Run BindingSiteFinder in its default mode with automatic parameter estimation.

Note: Within the racoon_clip workflow [8], enrichment of crosslink events over background (peak calling) is assessed using PureCLIP [9]. The resulting peak locations are provided as one BED file per sample and represent individual nucleotide positions with significantly enriched crosslink signal. These are used as a basis for defining binding sites which usually span 5–9 nt.

a. Define the input files.
    i. Provide the path to the peak file (generated by PureCLIP, in BED format)
    ii. Provide the path to the folder with the crosslink files (in bigWig format).

Note: If you specified multiple groups in racoon_clip, define binding sites separately for each group by selecting only the files for the given condition or separating files into distinct folders. In the code example below, all samples in this folder will be used.

```
# ----------------------
# get input from racoon_clip
# ----------------------

# PureCLIP file
pureclip_file <- "<path/to/racoon_clip_out_folder/results/peaks/
pureclip_sites.bed>"


# crosslink files (if all samples are from the same condition and
you did not specify groups in racoon_clip)
bw_dir <- "path/to/racoon_clip_out_folder/results/bw"

bw.plus <- list.files(bw_dir, pattern = "plus.bw$", full.names =
TRUE, recursive = TRUE)

bw.minus <- list.files(bw_dir, pattern = "minus.bw$", full.names =
TRUE, recursive = TRUE)
```

b. Load RDS files with stored gene and transcript regions from the annotation.

```
# read prepared annotation
gns <- readRDS(paste0(out, "gns.rds"))
regions <- readRDS(paste0(out, "regions.rds"))
```

c. Import the peaks, clean up the peaks object and check the number of peaks.

Note: The number of peaks should be the same as given in the chapter “Peak calling” of the Report.html from racoon_clip.

```
#-----------------
# peaks from PureCLIP
#-----------------

# import PureCLIP peaks
peaks = rtracklayer::import(con = pureclip_file,
                             format = "BED",
                             extraCols=c("additionalScores" =
"character"))

# clean PureCLIP peaks columns
peaks$additionalScores = NULL
peaks$name = NULL


# check number of peaks
NROW(peaks)
```

d. **Optional:** Remove scaffold chromosomes and the mitochondrial chromosome.

```
# optional: keep only standard chromosomes and drop chrM
peaks = keepStandardChromosomes(peaks, pruning.mode = "coarse")
%>%
     dropSeqlevels(., "chrM", pruning.mode = "coarse")
```

e. Generate a data frame with the metadata for BindingSiteFinder.

Critical: Check the printed metadata dataframe and make sure that the paths and conditions are specified correctly.

```
# --------------------
# make metadata for BindingSiteFinder
# --------------------

meta = data.frame(
  id = c(1,2), # give each sample a unique id
  condition = c("cond_A", "cond_A"), # add the condition for each
sample (used for differential analysis), but one BSF object per
condition needs to be used
  clPlus = bw.plus, # crosslinks plus strand bigwig files
  clMinus = bw.minus) # crosslinks minus strand bigwig files

meta
```

f. Run BSFind() for automatic binding site generation.

Note: We recommend disabling the gene-wise filter (cutoff.geneWiseFilter = 0) as automatic estimation for this parameter is often overly stringent.

```
# --------------------
# run BindingSiteFinder in automatic mode
# --------------------

# make BSF object
bds_object = BSFDataSetFromBigWig(ranges = peaks,
                           meta = meta,
                           silent = TRUE)

# compute initial binding sites allowing BindingSiteFinder to
estimate most parameters
bds_automatic = BSFind(bds_object,
                 anno.genes = gns,
                 anno.transcriptRegionList = regions,
                 cutoff.geneWiseFilter = 0)


# save automatic binding sites
saveRDS(bds_automatic, paste0(out, "bds_automatic.rds"))
```

g. Get a summary of BSF object including the number of binding sites (#N Ranges), the width of the binding sites (Width ranges) and the number of samples that were considered for binding site definition (#N Samples).

```
# binding site summary
bds_automatic
```

h. Display the performed steps including the automatically estimated parameters in a workflow chart (**Figure S4A**).

```
# visualize steps and filters
processingStepsFlowChart(bds_automatic)
```

Visual inspection of binding sites

This step will enable visual inspection of the defined binding sites and their comparison to crosslink sites and PureCLIP peaks defined in racoon_clip.

7. Visually inspect the generated binding sites in a genome browser.

Note: The following steps are given for the Integrative Genomics Viewer (IGV) but can be transferred to other genome browsers like the UCSC Genome Browser.

a. Save the binding sites as BED file.

```
# export binding sites as BED file
exportToBED(bds_automatic, con = paste0(out,
"BindingSites_automatic.bed"))
```

b. Load data in IGV.

i. Open IGV and select your genome.
ii. Load crosslink bigWig files from the folder bw_merged.
iii. Turn off the windowing function
iv. ("None") by right-click on the sample names.
v. Load peak BED file(s) with the PureCLIP-called peaks from the folder peaks.
vi. Load the BED file BindingSites_automatic.bed with the binding sites exported in the previous step.

c. Navigate to genes of interest and/or housekeeping genes with binding sites.

d. Inspect the distribution of crosslink events within and around the binding sites.

Note: With sufficient coverage, the crosslink events in a binding site usually follow a bell-shaped distribution. For comparison between binding sites (and between samples), keep in mind that the signal intensity both within the binding sites and in the surrounding background is proportional to the expression of the gene (and the sequencing depth of the sample).

i. Evaluate binding site width.

Note: Check the following: Do the defined binding sites accurately capture the spread of the crosslink events? Should the binding sites be wider or narrower? Refer to the section "Optimize binding site width" section to evaluate this further and possibly change the width accordingly.

ii. Evaluate filter settings

Note: Check the following: Do many binding sites hardly rise over background or, inversely, are many prominent looking, bell-shaped patterns not called as binding sites? In this case, refer to the section "Selecting custom cutoffs".

Note: Beware that not all binding sites will look perfect with any parameter settings. Try to find a combination of parameters for which the majority looks good.

Optimize binding site width

This step allows to choose the optimal binding site width based on the signal-to-flank ratios.

8. Evaluate how BindingSiteFinder decided for the optimal binding site width.

Note: BindingSiteFinder computes the signal-to-flank ratio, i.e., the ratio of crosslink events within the binding sites over equal-sized windows to both sides, for a range of user-defined widths (**Figure S4B**). To account for different binding strengths, the ratios are averaged over calculations with increasing gene-wise filter, i.e., each time excluding an additional 10% of ranked PureCLIP-called peaks per gene (geneWiseFilter from 0 to 0.9). The optimal width is then chosen to maximize the median signal-to-flank ratio across all binding sites.

Note: The width is always uneven because the binding sites are aligned to the highest signal in the center and then resized evenly to both sides [5].

a. Generate width estimation plots with estimateBsWidthPlot().

```
# check estimation of binding site width
estimateBsWidthPlot(bds_automatic)
```

b. Compare the signal-to-flank ratios across alternative widths (**Figure S4B**).

c. Generate binding sites with alternative widths with makeBindingSites() using a range of values for the parameter bsSize.

Note: The example code below implements binding sites of width 5, 7, and 9.

d. Use the function rangeCoveragePlot() to visualize the summed coverage of crosslink events across binding sites for each width (**Figure S4C**).

```
# --------------------
# compare different binding site widths
# --------------------

# compute binding sites with width 5, 7, and 9
bds1 <- makeBindingSites(object = bds_object, bsSize = 5)
bds2 <- makeBindingSites(object = bds_object, bsSize = 7)
bds3 <- makeBindingSites(object = bds_object, bsSize = 9)
# summarize in list
l = list(`automatic - bsSize = 5` = bds1,
         `close by  - bsSize = 7` = bds2,
         `close by  - bsSize = 9` = bds3)

# plot comparison
rangeCoveragePlot(l, width = 20, show.samples = F,
subset.chromosome = "chr1")
```

i. Evaluate how well the binding site boundaries (grey box) capture the crosslinking signal (blue line).

Note: Crosslink signal should peak in the centre and decline toward background at both edges.

e. Optional: To change the bs width to a more suitable number, rerun BSFind() with the selected width (here: bsSize = 7).

Note: We chose a width of 5 nucleotides for the U2AF2 data shown, but 7 nucleotides would also be a valid option. If analyzing multiple CLIP data sets of the same protein, select the same width for all to allow for direct comparisons.

```
# ---------------------
# optional: use a different width
# ---------------------

bds_selected_width = BSFind(bds_object,
                  anno.genes = gns,
                  anno.transcriptRegionList = regions,
                  bsSize = 7,
                  cutoff.geneWiseFilter = 0)

# check new number of binding sites

bds_selected_width
```

f. Export the new binding sites as BED file and load them in IGV for visual inspection (**Figure S4D**).

```
# save and export new binding sites
saveRDS(bds_selected_width, paste0(out, " bds_selected_width.rds"))

exportToBED(bds_selected_width, con = paste0(out,
"BindingSites_resize.bed"))
```

Select custom cutoffs (optional)

BindingSiteFinder implements multiple additional parameters that can be used to fine-tune the criteria for binding site definition. Here, we showcase the genewise filter. For a full description of the available parameters, refer to the documentation of BindingSiteFinder.

9. **Optional:** Apply a custom gene-wise filter (here 0.1).

Note: The parameter geneWiseFilter allows to exclude a user-defined fraction of PureCLIP-called peaks per gene from binding site definition. This can be useful for focusing on the most prominent binding sites per genes.

```
# --------------------
# optional: use a different gene-wise filter
# --------------------

bds_genewisefilter = BSFind(bds_object,
                 anno.genes = gns,
                 anno.transcriptRegionList = regions,
                 bsSize = 7,
                 cutoff.geneWiseFilter = 0.1)

# check new number of binding sites

bds_genewisefilter
```

Reproducibility filter

BindingSiteFinder uses a two-step procedure in which binding sites are first defined on the merged samples—thereby increasing the signal-to-background ratio—and then tested for reproducibility across the individual samples. The stringency of the reproducibility filter can be adjusted by the user.

Note: If only one sample is available (not recommended!), the default value n-1 for repro.nReps in BSFind() will not work. In this case, turn off reproducibility filtering by setting repro.nReps = 1 and repro.cutoff = 0.

10. Assess the reproducibility of binding sites across samples.

    a. Evaluate the signal distribution and minimum crosslink events per binding site chosen for each sample. The function reproducibilityFilterPlot() visualizes the distribution of crosslink events per binding site, indicating the number of crosslink events corresponding to the selected percentile cutoff (**Figure S4E**).

```
# plot support cutoffs used for each sample
reproducibilityFilterPlot(bds_selected_width)
```

Note: repro.cutoff defines the minimum number of crosslink events that are required to support a binding site. It is defined as a percentile—rather than an absolute number—to account for differences in signal depth between samples [5].

    b. Evaluate how many binding sites are supported by a sufficient number of samples defined by repro.nReps (**Figure S4F**).

Critical: If only two replicates are available, we recommend requiring support from both (repro.nReps = 2). With more replicates, require support by all or all but one sample. n-1 samples or less is advisable if some samples are considerably worse.

```
# intersections of supported binding sites per sample
reproducibilitySamplesPlot(bds_selected_width)
```

c. Re-run BSFind() with the selected reproducibility parameters.

Note: A higher cutoff (repro.cutoff = 0.1 or more) can be useful if the data shows high background. However, in shallow data sets, higher reproducibility cutoffs can lead to an overly stringent loss of binding sites.

```
# make binding sites using new reproducibility cutoffs
bds_repro = BSFind(bds_object,
                   anno.genes = gns,
                   anno.transcriptRegionList = regions,
                   bsSize = 5, # add the width you selected
                   cutoff.geneWiseFilter = 0,
                   repro.nReps = 2,
                   repro.cutoff = 0.1)

# get numbers
bds_repro

# plot reproducibility support with changed settings
reproducibilityFilterPlot(bds_repro)
reproducibilitySamplesPlot(bds_selected_repro)
```

d. Save the final binding sites as RDS file and export them as a BED file.

```
# get final binding sites
bds_final <- bds_selected_width

# save and export
saveRDS(bds_final, paste0(out, "bds_final.rds"))
exportToBED(bds_final, con = paste0(out,
"binding_sites_final.bed"))
```

e. Load the binding sites as BED file in IGV. To evaluate reproducibility, load the crosslinks (bigWig files) of the individual samples for comparison.

Characterization of binding sites

This step assigns each binding site to a target gene and a transcript region.

11. Assign binding sites to target genes.

Critical: In regions of overlapping annotations, binding sites cannot be unambiguously assigned to a single gene. One possibility to resolve such overlaps in BindingSiteFinder is by applying a hierarchy of gene biotypes.

Note: Since iCLIP data is strand-specific, this problem does not apply for genes on opposite strands.

a. Inspect the gene biotypes that are present in the annotation.

```
# present gene types
unique(gns$gene_type)
```

Note: The GENCODE annotation used here contains the following biotypes: protein_coding transcribed_unprocessed_pseudogene, processed_pseudogene, lncRNA, transcribed_unitary_pseudogene, transcribed_processed_pseudogene, unprocessed_pseudogene, IG_V_pseudogene, unitary_pseudogene, TR_V_pseudogene, IG_V_gene, snRNA, miRNA, misc_RNA, snoRNA, rRNA_pseudogene, rRNA, vault_RNA, TR_V_gene, Mt_tRNA, Mt_rRNA, IG_C_gene, IG_J_gene, TR_J_gene, TR_C_gene, TR_J_pseudogene, IG_D_gene, ribozyme, IG_C_pseudogene, TR_D_gene, TEC IG_J_pseudogene, scaRNA, translated_processed_pseudogene, artifact, sRNA, IG_pseudogene.

b. Visualize the number of binding sites that overlap with multiple gene biotypes.

```
# binding sites with overlapping gene biotypes
geneOverlapsPlot(bds_final)
```

c. Select which gene biotypes should be kept separately, depending on your protein of interest.

```
# decide which gene biotypes to keep separately
my_gene_types <- c("protein_coding", "lncRNA", "snRNA", "snoRNA",
"miRNA", "rRNA", "misc_RNA", "tRNA")
```

d. Collapse all pseudogene categories into "pseudogenes" and all remaining categories into "other".

```
# make hierarchy column
gns <- as.data.frame(gns) %>%
  dplyr::mutate(gene_type_plot = case_when(
    gene_type %in% my_gene_types ~ gene_type,
    grepl(gene_type, pattern = "pseudogene") ~ "pseudogene",
    TRUE ~ "other"
  )) %>%
  makeGRangesFromDataFrame(., keep.extra.columns = TRUE)
```

e. Define the hierarchy of the gene biotypes with a vector sorted by decreasing relevance.

```
# define hierarchy of interesting gene biotypes
hierarchy <- c("protein_coding", "lncRNA", "snRNA", "snoRNA",
"miRNA", "rRNA", "misc_RNA", "tRNA", "pseudogene", "other")
```

f. Apply the hierarchy and plot the resulting distribution of binding sites across the gene biotypes (**Figure S4H**).

```
# assign gene type according to hierarchy
bds_alt_gene_asignment <-
assignToGenes(bds_final,
                        overlaps = "hierarchy",
                        overlaps.rule = hierarchy,
                        anno.genes = gns,
                        match.geneType = "gene_type_plot"
                                       )
# plot
targetGeneSpectrumPlot(bds_alt_gene_asignment, showNGroups = 20)
```

12. Assign the binding sites to the transcript regions in which they are located. As most genes contain multiple transcript isoforms, overlaps are again resolved with a hierarchy.

    a. Visualize the amount of ambiguous binding sites overlapping with multiple transcript regions (**Figure S4I**).

```
# visualize binding sites overlapping with multiple transcript
regions
transcriptRegionOverlapsPlot(bds final)
```

b. Select a hierarchy which best fits to the expected binding behavior of your protein of interest. For U2AF2 as a known splicing factor, we give the highest priority to introns, followed by the untranslated regions and the coding region.

```
# transcript region hierarchy
region_hierarchy <- c("INTRON", "UTR5", "UTR3", "CDS")
```

Note: The names of the regions need to be identical to the names assigned when preparing the transcript regions object from the annotation.

c. Assign the binding sites to the transcript regions and plot the resulting distribution (**Figure S4J**).

```
# assign binding sites to transcript regions by the chosen
hierarchy

bds_final <-
assignToTranscriptRegions(bds_final,
                          overlaps = "hierarchy",
                          overlaps.rule = region_hierarcy,
anno.transcriptRegionList = regions)

# plot
transcriptRegionSpectrumPlot(bds_final, show.others = TRUE)
```

EXPECTED OUTCOMES

As a showcase example, we defined U2AF2 binding sites from the iCLIP3 data from HeLa cell lysates containing 250 μg, 100 μg, and 40 μg of total protein. Selected plots from the BindingSiteFinder analysis for the 250 μg U2AF2 samples are shown in **Figure S4**, including the workflow summary, the estimation of the optimal binding site width, the assessment of reproducibility and the assignment of binding sites to target genes and transcript regions. An HTML report showing the complete BindingSiteFinder analysis including diagnostic plots and other visualizations is provided in **Data S3**.

The newly generated iCLIP3 samples show a strong qualitative and quantitative agreement to a previously published U2AF2 iCLIP2 dataset from the same cell line (**Figures S4** and

S5)[6]. Furthermore, the comparison between the 250 µg, 100 µg, and 40 µg samples highlights the robustness of iCLIP3 for smaller amounts of RNA input.

As each RBP exhibits distinct binding behavior, the number of detected binding sites as well as their distribution across gene biotypes and transcript regions will differ. In general, these distributions are expected to reflect the known biology of the RBP. However, not all binding sites necessarily follow canonical binding patterns, and RBPs rarely show exclusive binding to a single transcript region due to non-canonical interactions and annotation ambiguities. For example, U2AF2 binding sites were predominantly associated with protein-coding genes and intronic regions, consistent with its established role in pre-mRNA splicing (**Figures S4H–J**). Moreover, even deeply sequenced CLIP datasets are unlikely to capture the complete repertoire of binding sites for globally acting RBPs, making the total number of detected sites dependent on signal depth. Nevertheless, the relative distribution across gene biotypes and transcript regions should ideally remain largely stable across varying signal depths.

LIMITATIONS

Defining a hierarchy of gene biotypes and transcript regions requires prior knowledge about the protein of interest and can potentially skew the resulting distribution. To avoid such biases, orient the hierarchy on distribution of uniquely assigned binding sites or use a majority vote (see BindingSiteFinder documentation for more details). Moreover, for the interpretation of bound transcript regions, it is important to keep in mind that some regions, particularly introns, are considerably longer than others. In addition, iCLIP signal intensity and PureCLIP scores are strongly influenced by transcript abundance. Consequently, binding events in lowly expressed genes may remain undetected, whereas highly expressed or housekeeping genes may appear overrepresented.

Background signal in iCLIP datasets can arise from co-purified RBPs, antibody-related artifacts, and other experimental sources. While this protocol incorporates statistical modeling of crosslink events through PureCLIP and reproducibility filtering through BindingSiteFinder, these approaches are primarily designed to reduce local crosslinking noise and sample-specific variability. Background arising from co-purified RBPs or nonspecific interactions may persist and cannot currently be distinguished with high confidence. It is therefore important to minimize background already during experimental design and sample preparation by carefully optimizing crosslinking conditions, antibody specificity, purification procedures, and quality control steps. Control datasets such as size-matched input (SMInput) or IgG controls, as well as comparisons against other iCLIP datasets, can help identify likely artifacts. Moreover, regions repeatedly detected across unrelated CLIP experiments are increasingly recognized as likely artifacts and collected in blacklists that can be used to exclude dubious binding sites [10,11]. However, no universal framework exists for discriminating specific versus nonspecific interactions, and

interpretation should always consider the experimental context and known properties of the RBP under investigation.

Finally, overlap between biological replicates or independent experiments is never expected to be complete. Variability in crosslink efficiency, sequencing depth, library complexity, and peak calling contributes to incomplete overlap even under well-controlled conditions. Therefore, reproducibility should be assessed quantitatively and interpreted in the context of experimental noise rather than assuming perfect overlap of binding sites.

TROUBLESHOOTING

Problem 1

A high number of binding sites is removed in the reproducibility filter step (related to Step 10).

Potential solution

Check in the reproducibilityFilterPlot whether certain samples show much lower signal than others. To avoid that weak samples impair the overall reproducibility, reduce the minimum number of samples required to support a binding site or completely remove the given samples from the analysis.

Problem 2

The binding site definition with BindingSiteFinder fails (related to Step 6).

Potential solution

A possible reason is that the number of crosslink events and/or PureCLIP-called peaks is too low. This can sometimes be circumvented by turning off the automatic estimation of binding width by directly setting the parameter bsSize to the desired value. Go back to the racoon_clip report to evaluate data quality across the analysis.

Problem 3

Presumed nice peaks are not captured as binding sites (related to Step 7).

Potential solution

This may result from a lack of reproducibility (see above) or a generally low signal in the region, usually occurring on lowly expressed transcripts. As a rule of thumb, peaks below 20 crosslink events are close to background levels and not always found by PureCLIP.

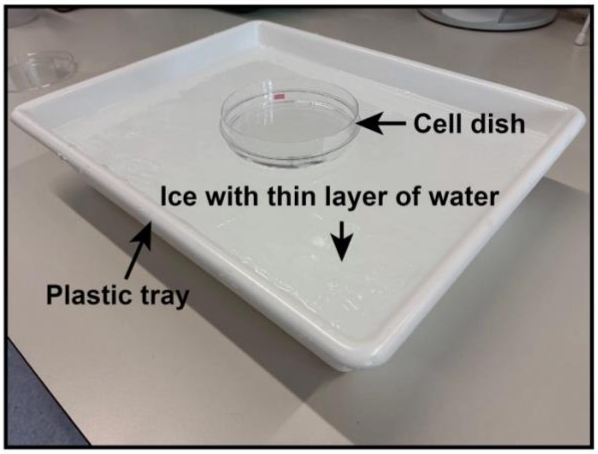

Figure S1 | Ice tray for UV crosslinking. A plastic tray that fits inside the UV crosslinker is filled with water and kept at -20ºC until the water is frozen. Once the ice is formed, the tray is taken out and a thin layer of water is added on top of it. The cell culture dishes without lid are arranged carefully on the ice tray, which is then placed inside the UV crosslinker.

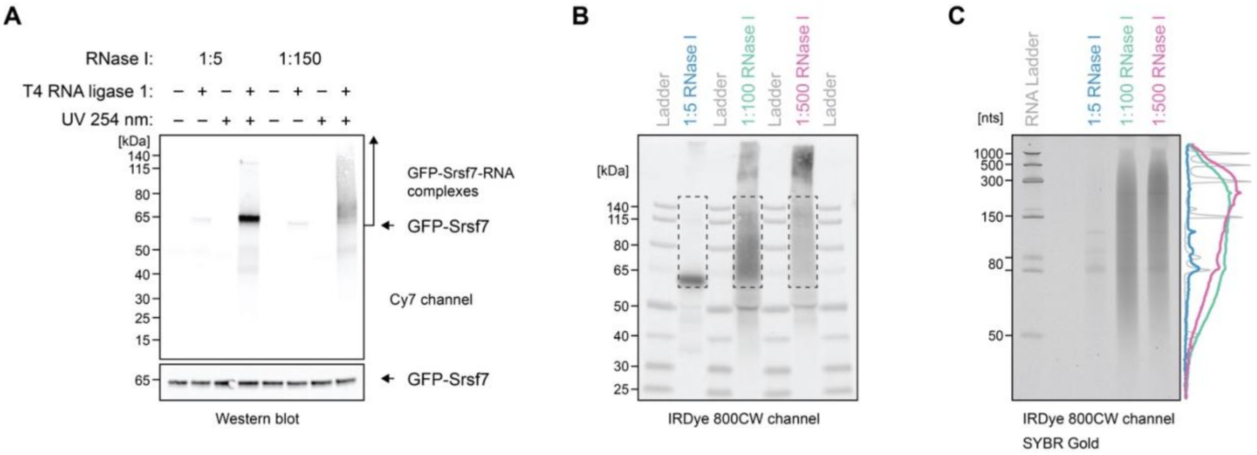

Figure S2 | iCLIP3-based visualization of immunopurified protein–RNA complexes. (A) A nitrocellulose membrane shows pCp-IR750-labeled GFP-Srsf7-RNA complexes immunopurified from mouse P19 cells under different RNase I digestion conditions (high, 20 U/µL, 1:5 dilution; medium, 0.67 U/µL, 1:150 dilution). Only 10% of the immunoprecipitation (IP) material was subjected to pCp-IR750 RNA labeling. Western blot below confirms equal GFP-Srsf7 immunoprecipitation across samples. **(B)** Nitrocellulose membrane showing pCp-IR750-labeled RNA on 10% of the immunopurified U2AF2–RNA complexes from UV-irradiated samples treated with three RNase I concentrations (high, 20 U/µL, 1:5 dilution; medium, 1 U/µL, 1:100 dilution; low, 0.2 U/µL, 1:500 dilution). The remaining 90% of the IP material was unlabeled but run on the same gel. Dashed boxes indicate the membrane regions from which RNA was isolated. **(C)** RNA isolated from the membrane regions shown in (D) was labeled with pCp-IR750 and resolved on a 10% TBE–urea gel alongside a low-range single-stranded RNA ladder. The gel was imaged in SYBR Gold and IRDye 800CW channels.

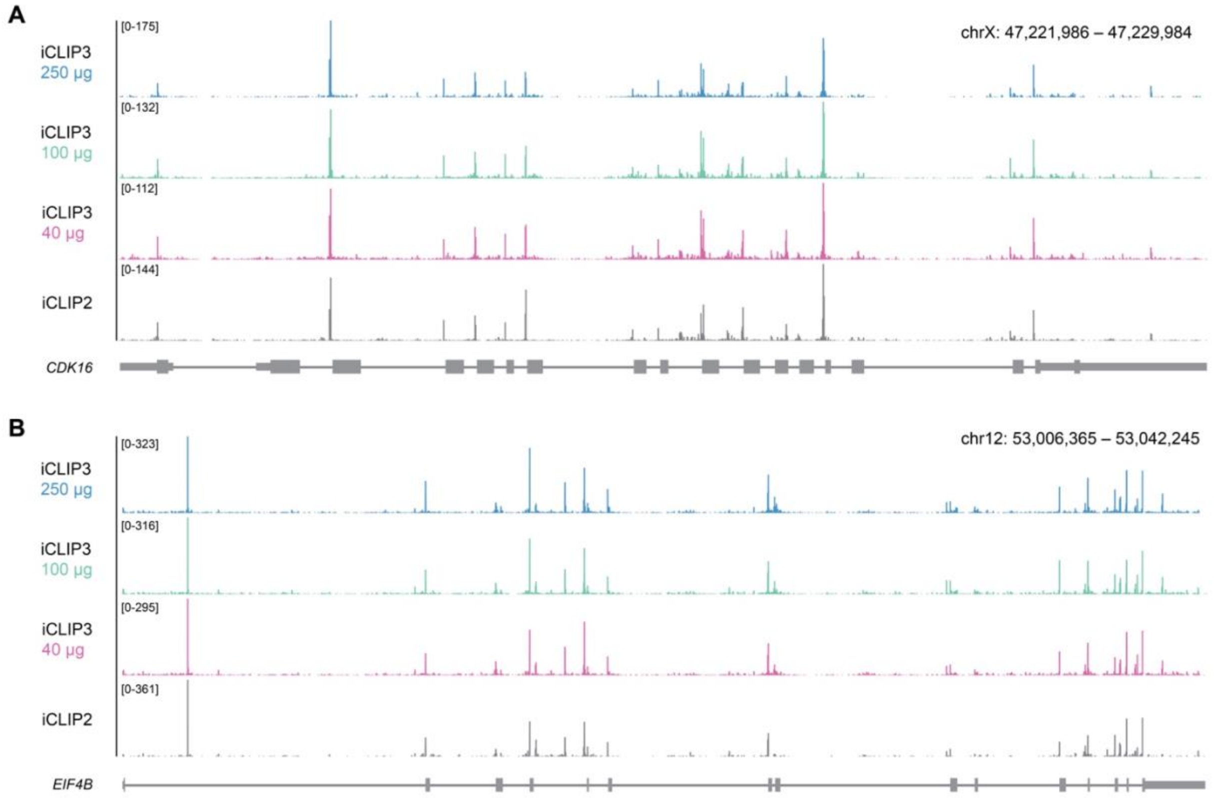

Figure S3 | Comparison between U2AF2 iCLIP3 and iCLIP2 crosslink events. Genome browser views of U2AF2 crosslink events (sum of replicates) across the genes **(A)** *CDK16* and **(B)** *EIF4B*.

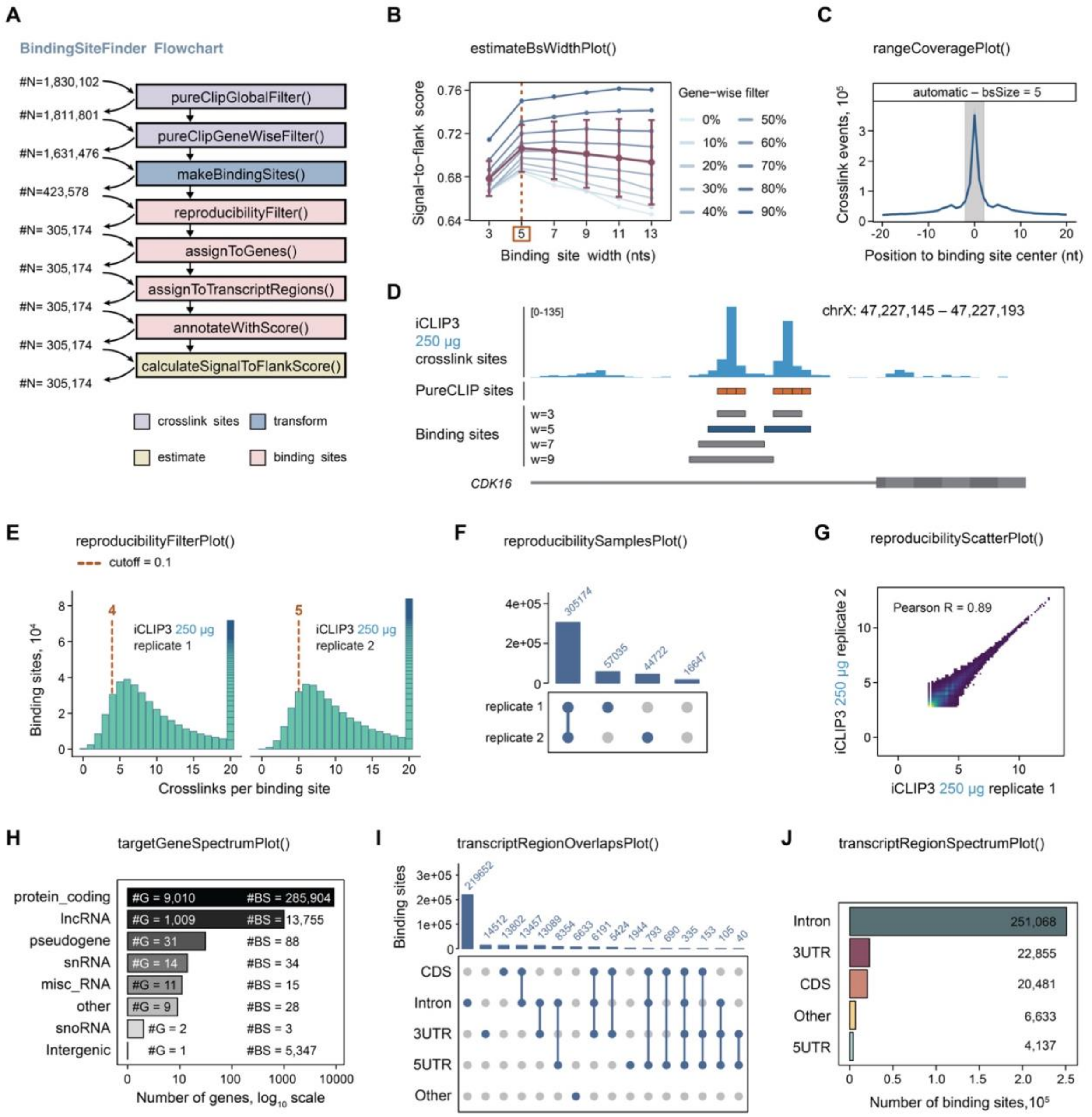

Figure S4 | Definition of binding sites with BindingSiteFinder. Exemplary visualizations for the 250 µg U2AF2 iCLIP3 sample. **(A)** BindingSiteFinder workflow summary. Numbers indicate peak or binding site counts at each step before or after binding site definition, respectively. **(B–D)** Estimation of optimal binding site width. **(B)** estimateBsWidthPlot displays signal-to-flank scores (y-axis) across candidate widths (x-axis), calculated for ten peak subsets generated by gene-wise filtering (0–90%; light to dark blue). Mean and standard deviation per width are marked in dark red, and suggested optimal width (here: 5 nt) in orange dashed line. **(C)** rangeCoveragePlot depicts metaprofile of crosslink events (y-axis) in 20-nt window around binding site center (x-axis); the grey box indicates given width.

(D) Genome browser view of the gene *CDK16* (chrX:47,227,145- 47,227,193) with U2AF2 iCLIP3 crosslink signal (blue), PureCLIP peaks (orange) and binding sites of 3, 5, 7 and 9 nt width (grey /dark blue). **(E–G)** Reproducibility filtering. **(E)** reproducibilityFilterPlot shows number of binding sites (y-axis) with given number of crosslinks (x-axis) for each replicate with selected cutoff for a binding site being supported (repro.cutoff = 0.1; corresponding 4 and 5 crosslink events for replicates 1 and 2, respectively). **(F)** reproducibilitySamplesPlot displays intersections of supported sites between replicates. **(G)** reproducibilityScatterPlot shows crosslink counts per binding site (log_2 scale) in replicate 1 (x-axis) versus replicate 2 (y-axis), colored by point density; Pearson correlation coefficient is indicated. **(H–J)** Assignment of binding sites to bound genes and transcript regions. **(H)** targetGeneSpectrumPlot summarizes bound genes by gene biotype, indicating gene biotype (left) and binding site counts (right). **(I)** transcriptRegionOverlapsPlot shows ambiguous assignments to multiple transcript regions. **(J)** Final distribution of binding sites across transcript regions after hierarchical assignment of ambiguous binding sites. An HTML report with the complete BindingSiteFinder analysis is provided in **Data S3**.

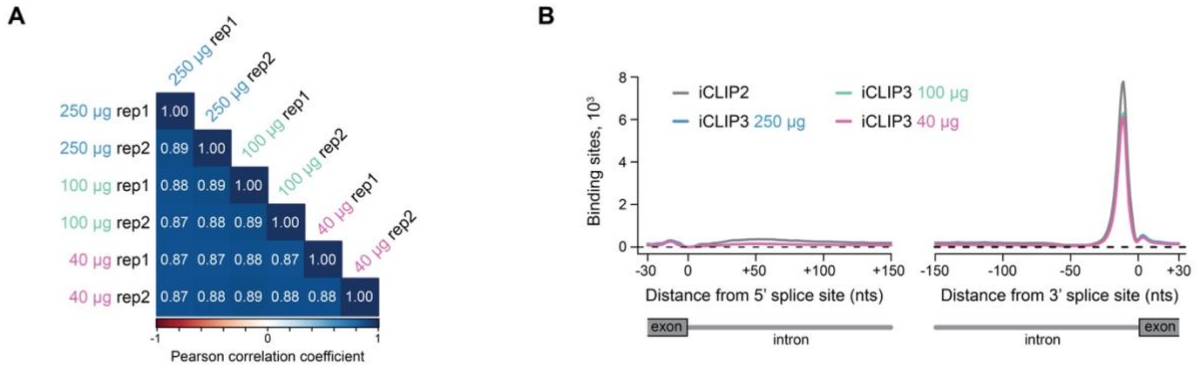

Figure S5 | Comparison between U2AF2 iCLIP3 and iCLIP2 binding sites. (A) Pairwise Pearson correlation coefficients for all U2AF2 iCLIP3 replicates based on the number of crosslink events in overlapping binding sites. **(B)** Metaprofile of binding sites around 5′ and 3′ splice sites (smoothed lines). blue – iCLIP3 250 µg, green - iCLIP3 100 µg, pink – iCLIP3 40 µg, grey – published U2AF2 iCLIP2 data (GSM6793346, GSM6793347)[6].

References:

1. Hentze, M.W., Sommerkamp, P., Ravi, V., and Gebauer, F. (2025). Rethinking RNA-binding proteins: Riboregulation challenges prevailing views. Cell *188*, 4811-4827. 10.1016/j.cell.2025.06.021.
2. Huppertz, I., Attig, J., D'Ambrogio, A., Easton, L.E., Sibley, C.R., Sugimoto, Y., Tajnik, M., König, J., and Ule, J. (2014). iCLIP: protein-RNA interactions at nucleotide resolution. Methods *65*, 274-287. 10.1016/j.ymeth.2013.10.011.
3. Brüggemann, M., Zarnack, K. (2021). BindingSiteFinder – Binding site definition based on iCLIP data. Bioconductor DOI: 10.18129/B9.bioc.BindingSiteFinder
4. Sugimoto, Y., König, J., Hussain, S., Zupan, B., Curk, T., Frye, M., and Ule, J. (2012). Analysis of CLIP and iCLIP methods for nucleotide-resolution studies of protein-RNA interactions. Genome Biol *13*, R67. 10.1186/gb-2012-13-8-r67.
5. Busch, A., Brüggemann, M., Ebersberger, S., and Zarnack, K. (2020). iCLIP data analysis: A complete pipeline from sequencing reads to RBP binding sites. Methods *178*, 49-62. 10.1016/j.ymeth.2019.11.008.
6. Ebersberger, S., Hipp, C., Mulorz, M.M., Buchbender, A., Hubrich, D., Kang, H.S., Martínez-Lumbreras, S., Kristofori, P., Sutandy, F.X.R., Llacsahuanga Allcca, L., et al. (2023). FUBP1 is a general splicing factor facilitating 3' splice site recognition and splicing of long introns. Mol Cell *83*, 2653-2672.e2615. 10.1016/j.molcel.2023.07.002.
7. Huber, W., Carey, V.J., Gentleman, R., Anders, S., Carlson, M., Carvalho, B.S., Bravo, H.C., Davis, S., Gatto, L., Girke, T., et al. (2015). Orchestrating high-throughput genomic analysis with Bioconductor. Nat Methods *12*, 115-121. 10.1038/nmeth.3252.
8. Klostermann, M., and Zarnack, K. (2024). racoon_clip-a complete pipeline for single-nucleotide analyses of iCLIP and eCLIP data. Bioinform Adv *4*, vbae084. 10.1093/bioadv/vbae084.
9. Krakau, S., Richard, H., and Marsico, A. (2017). PureCLIP: capturing target-specific protein-RNA interaction footprints from single-nucleotide CLIP-seq data. Genome Biol *18*, 240. 10.1186/s13059-017-1364-2.
10. Boyle, E.A., Her, H.L., Mueller, J.R., Naritomi, J.T., Nguyen, G.G., and Yeo, G.W. (2023). Skipper analysis of eCLIP datasets enables sensitive detection of constrained translation factor binding sites. Cell Genom *3*, 100317. 10.1016/j.xgen.2023.100317.
11. Van Nostrand, E.L., Freese, P., Pratt, G.A., Wang, X., Wei, X., Xiao, R., Blue, S.M., Chen, J.Y., Cody, N.A.L., Dominguez, D., et al. (2020). A large-scale binding and functional map of human RNA-binding proteins. Nature *583*, 711-719. 10.1038/s41586-020-2077-3.
