# Supplementary material for: Isotope-free mapping of protein-RNA interactions at single-nucleotide resolution by iCLIP3
Source: STAR Protoc. 2026 Jul 17;7(3):104704. doi: 10.1016/j.xpro.2026.104704 (PMC13400664; doi:10.1016/j.xpro.2026.104704)
Supplement: Data S1. Full processing report for U2AF2 iCLIP3 samples [file mmc2.zip › Data S1. Full processing report for U2AF2 iCLIP3 samples.html]

racoon\_clip processing report


# racoon\_clip processing report

#### Melina Klostermann

#### 25 February, 2026

# 1 Overview of steps performed

**racoon\_clip peaks** was used.

# 2 Configurations

Here is a summary of the settings you have chosen for this
analysis.

## 2.1 Input files

Input files and reference data used in the analysis

|  | Input |
| --- | --- |
| infiles | /data/42-julia-hpc-bio-zarnack/mek24iv/projects/04\_iCLIP3/raw\_files/U2AF2\_iCLIP3\_fastq/imb*ug*.fastq.gz |
| barcodes\_fasta |  |
| gtf | /data/42-julia-hpc-bio-zarnack/mek24iv/annotations/gencode.v49/gencode.v49.primary\_assembly.annotation.gtf |
| genome\_fasta | /data/42-julia-hpc-bio-zarnack/mek24iv/annotations/GRCh38.p14.genome.fa |
| fastqScreen\_config | /data/42-julia-hpc-bio-zarnack/mek24iv/projects/04\_iCLIP3/iCLIP2vs3\_cluster\_scripts/U2AF2/03\_racoon\_clip\_iCLIP2/fastqscreen.config |

## 2.2 Options

Analysis workflow options and settings

|  | Option |
| --- | --- |
| workflow\_type | peaks |
| demultiplex | FALSE |
| adapter\_trimming | TRUE |
| trim3 | TRUE |
| quality\_filter\_barcodes | FALSE |
| encode | FALSE |
| deduplicate | TRUE |
| fastqScreen | TRUE |

## 2.3 Barcode settings

Barcode and UMI configuration parameters

|  | Setting |
| --- | --- |
| barcodeLength | 0 |
| minBaseQuality | 10 |
| umi1\_len | 9 |
| umi2\_len | 0 |
| total\_barcode\_len | 9 |
| samples | imb\_koenig\_2025\_07\_01\_u2af65\_40ug\_rep1.R1 imb\_koenig\_2025\_07\_02\_u2af65\_40ug\_rep2.R1 imb\_koenig\_2025\_07\_03\_u2af65\_100ug\_rep1.R1 imb\_koenig\_2025\_07\_04\_u2af65\_100ug\_rep2.R1 imb\_koenig\_2025\_07\_05\_u2af65\_250ug\_rep1.R1 imb\_koenig\_2025\_07\_06\_u2af65\_250ug\_rep2.R1 |
| encode | FALSE |
| encode\_umi\_length | 10 |

## 2.4 Read filter

Read length filtering parameters

|  | Setting |
| --- | --- |
| min\_read\_length | 15 |

## 2.5 Peakcalling

Peak calling parameters for Pureclip

|  | Setting |
| --- | --- |
| morePureclipParameters |  |

## 2.6 Alignment setting (STAR)

STAR alignment parameters and settings

|  | Setting |
| --- | --- |
| read\_length | 150 |
| outFilterMismatchNoverReadLmax | 0.04 |
| outFilterMismatchNmax | 999 |
| outFilterMultimapNmax | 1 |
| outReadsUnmapped | Fastx |
| outSJfilterReads | Unique |
| moreSTARParameters |  |

## 2.7 Experimental groups

Experimental groups and sample assignments

| Group | Sample |
| --- | --- |
| u2af2\_40ug | imb\_koenig\_2025\_07\_01\_u2af65\_40ug\_rep1.R1 |
| u2af2\_40ug | imb\_koenig\_2025\_07\_02\_u2af65\_40ug\_rep2.R1 |
| u2af2\_100ug | imb\_koenig\_2025\_07\_03\_u2af65\_100ug\_rep1.R1 |
| u2af2\_100ug | imb\_koenig\_2025\_07\_04\_u2af65\_100ug\_rep2.R1 |
| u2af2\_250ug | imb\_koenig\_2025\_07\_05\_u2af65\_250ug\_rep1.R1 |
| u2af2\_250ug | imb\_koenig\_2025\_07\_06\_u2af65\_250ug\_rep2.R1 |

#

#

# 3 Input sequencing files

Input sequencing files summary

| Sample | Total.Sequences | Sequence.Length |
| --- | --- | --- |
| imb\_koenig\_2025\_07\_01\_u2af65\_40ug\_rep1.R1 | 61,993,752 | 122 |
| imb\_koenig\_2025\_07\_02\_u2af65\_40ug\_rep2.R1 | 70,180,871 | 122 |
| imb\_koenig\_2025\_07\_03\_u2af65\_100ug\_rep1.R1 | 74,690,516 | 122 |
| imb\_koenig\_2025\_07\_04\_u2af65\_100ug\_rep2.R1 | 55,601,104 | 122 |
| imb\_koenig\_2025\_07\_05\_u2af65\_250ug\_rep1.R1 | 59,046,851 | 122 |
| imb\_koenig\_2025\_07\_06\_u2af65\_250ug\_rep2.R1 | 67,003,798 | 122 |

# 4 Quality control of raw files

Initial quality control of the raw sequencing file is performed with
FastQC. Here you can see the sequencing quality and content per
base.

## 4.1 Quality scores across all bases

### imb\_koenig\_2025\_07\_01\_u2af65\_40ug\_rep1.R1

### imb\_koenig\_2025\_07\_02\_u2af65\_40ug\_rep2.R1

### imb\_koenig\_2025\_07\_03\_u2af65\_100ug\_rep1.R1

### imb\_koenig\_2025\_07\_04\_u2af65\_100ug\_rep2.R1

### imb\_koenig\_2025\_07\_05\_u2af65\_250ug\_rep1.R1

### imb\_koenig\_2025\_07\_06\_u2af65\_250ug\_rep2.R1

##

## 4.2 Sequence content over all bases

### imb\_koenig\_2025\_07\_01\_u2af65\_40ug\_rep1.R1

### imb\_koenig\_2025\_07\_02\_u2af65\_40ug\_rep2.R1

### imb\_koenig\_2025\_07\_03\_u2af65\_100ug\_rep1.R1

### imb\_koenig\_2025\_07\_04\_u2af65\_100ug\_rep2.R1

### imb\_koenig\_2025\_07\_05\_u2af65\_250ug\_rep1.R1

### imb\_koenig\_2025\_07\_06\_u2af65\_250ug\_rep2.R1

## 

**Note:** In CLIP-seq data, it is normal to observe an
enrichment of thymine (T) nucleotides at the crosslink position, which
occurs immediately following the barcode and UMI sequences. This
T-enrichment is a characteristic signature of UV-induced
crosslinking.

# 5 FastQ Screen contamination check

FastQ Screen was used to check for potential contamination from
different organisms. The plot shows the percentage of reads mapping to
each reference genome.

Contamination screening results showing percentage of reads mapping to
different reference genomes

| Sample | rRNA percentage | No hits percentage |
| --- | --- | --- |
| imb\_koenig\_2025\_07\_01\_u2af65\_40ug\_rep1.R1 | 3.61 | 96.39 |
| imb\_koenig\_2025\_07\_02\_u2af65\_40ug\_rep2.R1 | 3.62 | 96.38 |
| imb\_koenig\_2025\_07\_03\_u2af65\_100ug\_rep1.R1 | 3.82 | 96.18 |
| imb\_koenig\_2025\_07\_04\_u2af65\_100ug\_rep2.R1 | 3.64 | 96.36 |
| imb\_koenig\_2025\_07\_05\_u2af65\_250ug\_rep1.R1 | 3.91 | 96.09 |
| imb\_koenig\_2025\_07\_06\_u2af65\_250ug\_rep2.R1 | 3.98 | 96.02 |

# 6 Adapter trimming

**Adapter Content Detection and Trimming:**  
If FastQC reports “No adapter content found” in the raw files, this
indicates that adapter sequences were not detected by FastQC’s automated
detection. However, adapter trimming is still performed using FLEXBAR
with the adapter sequences specified in your configuration file. If no
file is provied, a default file is used, which contains standard
Illumina and eCLIP adapters.

The adapters are trimmed from the 3’ end of the read
(`--adapter-trim-end RIGHT`), with an allowed error of 0.1
(`--adapter-error-rate 0.1`). The adapter must overlap the
read by at least 1nt (`--adapter-min-overlap 1`). Adapter
trimming can be done in multiple cycles as specified by the user, here 1
(adapterCycles, see configurations). Reads shorter than 15 are discarded
(min\_read\_length, see configurations).

UMIs are written into the read header with (`--umi-tags`).
(If the data is from ENCODE, UMIs are already removed from the read and
written to the beginning of the header. As umi-tools dedup, which is
used here for deduplication, expects the UMI to be at the end of the
read, encode: True can be specified to move the UMI from the beginning
of the read header to the end (see configurations).)

Read counts through quality filtering and adapter trimming steps

| Sample | Raw reads | Trimmed reads |
| --- | --- | --- |
| imb\_koenig\_2025\_07\_01\_u2af65\_40ug\_rep1.R1 | 61,993,752 | 61,921,159 |
| imb\_koenig\_2025\_07\_02\_u2af65\_40ug\_rep2.R1 | 70,180,871 | 70,086,607 |
| imb\_koenig\_2025\_07\_03\_u2af65\_100ug\_rep1.R1 | 74,690,516 | 74,529,007 |
| imb\_koenig\_2025\_07\_04\_u2af65\_100ug\_rep2.R1 | 55,601,104 | 55,490,309 |
| imb\_koenig\_2025\_07\_05\_u2af65\_250ug\_rep1.R1 | 59,046,851 | 58,873,087 |
| imb\_koenig\_2025\_07\_06\_u2af65\_250ug\_rep2.R1 | 67,003,798 | 66,773,867 |

## 6.1 Adapter content before trimming

Position of adapters found in the reads. If adapters are found early
in the reads, this indicates over digestion of the samples.

**No adapter content was detected in the raw files.**

## 6.2 Adapter content after trimming

**Trimming successful, no adapter contents left.**

# 7 Alignment

The genome annotation is indexed using STAR
`--runMode genomeGenerate`. The merged file of chimeric reads
is then aligned to the genome annotation using STAR
`--runMode alignReads`. For STAR settings, see
Configurations. The resulting bam files are indexed using samtools
index.

STAR alignment statistics for all samples

|  | imb\_koenig\_2025\_07\_01\_u2af65\_40ug\_rep1.R1 | imb\_koenig\_2025\_07\_02\_u2af65\_40ug\_rep2.R1 | imb\_koenig\_2025\_07\_03\_u2af65\_100ug\_rep1.R1 | imb\_koenig\_2025\_07\_04\_u2af65\_100ug\_rep2.R1 | imb\_koenig\_2025\_07\_05\_u2af65\_250ug\_rep1.R1 | imb\_koenig\_2025\_07\_06\_u2af65\_250ug\_rep2.R1 |
| --- | --- | --- | --- | --- | --- | --- |
| Number of input reads | 61921159 | 70086607 | 74529007 | 55490309 | 58873087 | 66773867 |
| Uniquely mapped reads number | 50256899 | 56974722 | 60496417 | 45169554 | 47618925 | 53789877 |
| Uniquely mapped reads % | 81.16% | 81.29% | 81.17% | 81.40% | 80.88% | 80.56% |
| Number of reads mapped to multiple loci | 0 | 0 | 0 | 0 | 0 | 0 |
| % of reads mapped to multiple loci | 0.00% | 0.00% | 0.00% | 0.00% | 0.00% | 0.00% |
| Number of reads mapped to too many loci | 8229587 | 9323932 | 10030739 | 7326137 | 7990800 | 9129635 |
| % of reads mapped to too many loci | 13.29% | 13.30% | 13.46% | 13.20% | 13.57% | 13.67% |
| Number of reads unmapped: too many mismatches | 1774487 | 1864609 | 2008357 | 1508756 | 1625013 | 1931323 |
| % of reads unmapped: too many mismatches | 2.87% | 2.66% | 2.69% | 2.72% | 2.76% | 2.89% |
| Number of reads unmapped: too short | 1306307 | 1463893 | 1514064 | 1135026 | 1253501 | 1479675 |
| % of reads unmapped: too short | 2.11% | 2.09% | 2.03% | 2.05% | 2.13% | 2.22% |
| Number of reads unmapped: other | 353879 | 459451 | 479430 | 350836 | 384848 | 443357 |
| % of reads unmapped: other | 0.57% | 0.66% | 0.64% | 0.63% | 0.65% | 0.66% |

# 8 Deduplication

Reads are deduplicated with umi\_tools dedup
`--extract-umi-method read_id --method unique`.

Read counts from raw input through alignment and UMI-based deduplication

| Sample | Uniquely Mapped Reads | Deduplicated Reads |
| --- | --- | --- |
| imb\_koenig\_2025\_07\_01\_u2af65\_40ug\_rep1.R1 | 50,256,899 | 38,846,194 |
| imb\_koenig\_2025\_07\_02\_u2af65\_40ug\_rep2.R1 | 56,974,722 | 41,759,921 |
| imb\_koenig\_2025\_07\_03\_u2af65\_100ug\_rep1.R1 | 60,496,417 | 47,861,283 |
| imb\_koenig\_2025\_07\_04\_u2af65\_100ug\_rep2.R1 | 45,169,554 | 37,297,821 |
| imb\_koenig\_2025\_07\_05\_u2af65\_250ug\_rep1.R1 | 47,618,925 | 39,335,988 |
| imb\_koenig\_2025\_07\_06\_u2af65\_250ug\_rep2.R1 | 53,789,877 | 43,699,323 |

# 9 Obtaining crosslinks

The (deduplicated) bam files are then converted to bed files using
bedtools bamtobed. The reads are shifted 1nt upstream (5’ direction)
with bedtools shift -m 1 -p -1 because the UV crosslink should be
positioned 1nt earlier. To allow visualisation in a genome browser, the
1nt crosslink bed file is also converted to a .bigWig file using
bedGraphToBigWig. These bigWig files are then merged with bigWigMerge by
the experiment groups specified by the user.

Crosslink identification and nucleotide-level resolution statistics

|  | Uniquely mapped reads | Crosslink events (deduplicated reads) | Crosslinked nucleotides | Mean crosslink events per crosslinked nucleotide |
| --- | --- | --- | --- | --- |
| imb\_koenig\_2025\_07\_01\_u2af65\_40ug\_rep1.R1 | 50,256,899 | 38,846,194 | 27,931,051 | 1.39 |
| imb\_koenig\_2025\_07\_02\_u2af65\_40ug\_rep2.R1 | 56,974,722 | 41,759,921 | 29,491,846 | 1.42 |
| imb\_koenig\_2025\_07\_03\_u2af65\_100ug\_rep1.R1 | 60,496,417 | 47,861,283 | 32,840,148 | 1.46 |
| imb\_koenig\_2025\_07\_04\_u2af65\_100ug\_rep2.R1 | 45,169,554 | 37,297,821 | 26,580,153 | 1.40 |
| imb\_koenig\_2025\_07\_05\_u2af65\_250ug\_rep1.R1 | 47,618,925 | 39,335,988 | 27,521,061 | 1.43 |
| imb\_koenig\_2025\_07\_06\_u2af65\_250ug\_rep2.R1 | 53,789,877 | 43,699,323 | 30,017,408 | 1.46 |

# 10 Peak calling

Peaks are called with pureclip on the merged bam files of the sample
groups (or all samples if no groups are specified).

Number of peaks identified in each sample/group:

Number of peaks identified by Pureclip for each sample or group

| Group | Peaks |
| --- | --- |
| pureclip\_sites\_u2af2\_100ug | 1,911,344 |
| pureclip\_sites\_u2af2\_250ug | 1,833,522 |
| pureclip\_sites\_u2af2\_40ug | 1,757,429 |

# 11 Session info

```
## R version 4.5.2 (2025-10-31)
## Platform: x86_64-conda-linux-gnu
## Running under: Debian GNU/Linux 13 (trixie)
## 
## Matrix products: default
## BLAS/LAPACK: /data/42-julia-hpc-bio-zarnack/mek24iv/mamba_envs/f26c7ec36b6cb8a195817d034e46a6a9_/lib/libopenblasp-r0.3.30.so;  LAPACK version 3.12.0
## 
## locale:
##  [1] LC_CTYPE=en_US.UTF-8       LC_NUMERIC=C              
##  [3] LC_TIME=en_US.UTF-8        LC_COLLATE=en_US.UTF-8    
##  [5] LC_MONETARY=en_US.UTF-8    LC_MESSAGES=en_US.UTF-8   
##  [7] LC_PAPER=en_US.UTF-8       LC_NAME=C                 
##  [9] LC_ADDRESS=C               LC_TELEPHONE=C            
## [11] LC_MEASUREMENT=en_US.UTF-8 LC_IDENTIFICATION=C       
## 
## time zone: Europe/Berlin
## tzcode source: system (glibc)
## 
## attached base packages:
## [1] stats     graphics  grDevices utils     datasets  methods   base     
## 
## other attached packages:
##  [1] kableExtra_1.4.0 yaml_2.3.10      lubridate_1.9.4  forcats_1.0.1   
##  [5] stringr_1.6.0    dplyr_1.1.4      purrr_1.2.0      readr_2.1.6     
##  [9] tidyr_1.3.1      tibble_3.3.0     tidyverse_2.0.0  ggplot2_4.0.1   
## [13] knitr_1.50      
## 
## loaded via a namespace (and not attached):
##  [1] sass_0.4.10        generics_0.1.4     xml2_1.5.0         stringi_1.8.7     
##  [5] hms_1.1.4          digest_0.6.39      magrittr_2.0.4     evaluate_1.0.5    
##  [9] grid_4.5.2         timechange_0.3.0   RColorBrewer_1.1-3 fastmap_1.2.0     
## [13] plyr_1.8.9         jsonlite_2.0.0     viridisLite_0.4.2  scales_1.4.0      
## [17] textshaping_1.0.4  jquerylib_0.1.4    cli_3.6.5          rlang_1.1.6       
## [21] crayon_1.5.3       bit64_4.6.0-1      withr_3.0.2        cachem_1.1.0      
## [25] parallel_4.5.2     tools_4.5.2        reshape2_1.4.5     tzdb_0.5.0        
## [29] vctrs_0.6.5        R6_2.6.1           lifecycle_1.0.4    bit_4.6.0         
## [33] vroom_1.6.6        ragg_1.5.0         pkgconfig_2.0.3    pillar_1.11.1     
## [37] bslib_0.9.0        gtable_0.3.6       Rcpp_1.1.0         glue_1.8.0        
## [41] systemfonts_1.3.1  xfun_0.54          tidyselect_1.2.1   rstudioapi_0.17.1 
## [45] farver_2.1.2       htmltools_0.5.8.1  labeling_0.4.3     rmarkdown_2.30    
## [49] svglite_2.2.2      compiler_4.5.2     S7_0.2.1
```
