# Supplementary material for: Isotope-free mapping of protein-RNA interactions at single-nucleotide resolution by iCLIP3
Source: STAR Protoc. 2026 Jul 17;7(3):104704. doi: 10.1016/j.xpro.2026.104704 (PMC13400664; doi:10.1016/j.xpro.2026.104704)
Supplement: Data S2. BindingSiteFinder example codes [file mmc3.zip › Data S2. BindingSiteFinder example codes.html]

Define binding sites example code


## Table of contents

- 1 What is done here?
- 2 Input files
  - 2.1 Pureclip peaks
  - 2.2 Metadata for BindingSiteFinder
- 3 Make binding sites
  - 3.1 Use automatic binding site generation
  - 3.2 Check automatic binding sites (and decide what parameters should be changed for final binding site definition)
- 4 Control (and optionally change) the binding site width
  - 4.0.1 Optional: change to a new bs width
  - 4.0.2 Optional: change genewise filter
  - 4.1 Check reproducibility of binding sites and select cutoffs
    - 4.1.1 Optional: Change reproducibility support cutoff
  - 4.2 Export binding sites for IGV
- 5 Characterise binding sites
  - 5.1 Assign gene types
  - 5.2 Assignment of bound transcipt regions
    - 5.2.1 The problem: Multiple transcripts lead to ambiguty in binding site region
    - 5.2.2 Resolved transcript regions
- 6 Final binding sites
  - 6.1 Depict the processing steps for the final binding site definition
  - 6.2 Export binding sites
- 7 Session Info

# Define binding sites example code

 Code

- Show All Code
- Hide All Code
- ---
- View Source

Author

Melina Klostermann

Published

March 20, 2026

# 1 What is done here?

This is an exemplary code to define binding sites from racoon\_clip outputs. Here the replicates of the for the U2AF2 iCLIP3 dataset with 250ug RNA are used.

# 2 Input files

Show code

```
# ----------------------
# Get input from racoon_clip
# ----------------------
# pureclip files
pureclip_files <- "/Users/melinaklostermann/Documents/projects/iCLIP3/00_racoon_clip_processed_files/U2AF2_iCLIP3/pureclip_sites_u2af2_250ug.bed"


# bw files
bw_dir <- "/Users/melinaklostermann/Documents/projects/iCLIP3/00_racoon_clip_processed_files/U2AF2_iCLIP3"
bw.plus <- list.files(bw_dir, pattern = "u2af65_250ug.*\\plus.bw$", full.names = TRUE, recursive = TRUE)
bw.minus <- list.files(bw_dir, pattern = "u2af65_250ug.*\\minus.bw$", full.names = TRUE, recursive = TRUE)
```

Show code

```
# ----------------------
# Prepare annotation
# ----------------------
# Note: run only once to prepare annotation files for BSF and save them as rds files

# annotation file (download for example from GENCODE)
annoFile <- "/Users/melinaklostermann/Documents/projects/anno/GENCODEv49/gencode.v49.annotation.gtf.gz"
out_gns <- paste0(out, "gns.rds")
out_regions <- paste0(out, "regions.rds")

# prepare annotation for BSF
###########################
# Make annotation database from gff3 file
annoDb = txdbmaker::makeTxDbFromGFF(file = annoFile, format = "gtf")
annoInfo = rtracklayer::import(annoFile, format = "gtf")


# Get genes as GRanges
gns = genes(annoDb)
idx = match(gns$gene_id, annoInfo$gene_id)
meta = cbind(elementMetadata(gns),
             elementMetadata(annoInfo)[idx,])
meta = meta[,!duplicated(colnames(meta))]
elementMetadata(gns) = meta

saveRDS(gns, out_gns)


# Get regions as Granges
cdseq = cds(annoDb) 
intrns = unlist(intronsByTranscript(annoDb)) 
utrs3 = unlist(threeUTRsByTranscript(annoDb)) 
utrs5 = unlist(fiveUTRsByTranscript(annoDb)) 
regions = GRangesList(CDS = cdseq, INTRON = intrns, UTR3 = utrs3, UTR5 = utrs5)

saveRDS(regions, out_regions)
```

Show code

```
# read prepared annotation
gns <- readRDS(paste0(out, "gns.rds"))
regions <- readRDS(paste0(out, "regions.rds"))
```

## 2.1 Pureclip peaks

Show code

```
#-----------------
# Peaks from pureclip
#-----------------
pureclip_file <- "/Users/melinaklostermann/Documents/projects/iCLIP3/00_racoon_clip_processed_files/U2AF2_iCLIP3/peaks/pureclip_sites_u2af2_250ug.bed"
# read pureclip peaks
peaks  = rtracklayer::import(con = pureclip_file, 
                             format = "BED", 
                             extraCols=c("additionalScores" = "character"))

# clean pureclip peaks columns
peaks$additionalScores = NULL
peaks$name = NULL

# optionally keep only standard chromosomes and drop chrM
peaks = keepStandardChromosomes(peaks, pruning.mode = "coarse") %>% 
                 dropSeqlevels(., "chrM", pruning.mode = "coarse" )

# check number of peaks
NROW(peaks)
```

```
[1] 1830102
```

## 2.2 Metadata for BindingSiteFinder

Show code

```
# --------------------
# make metadata for BindingSiteFinder
# --------------------

meta = data.frame(
  id = c(1,2), # give each sample a unique id
  condition = c("250ug", "250ug"), # add the condition for each sample (used for differential analysis), but one BSF object per condition needs to be used
  clPlus = bw.plus, # crosslinks plus strand bigwig files
  clMinus = bw.minus) # crosslinks minus strand bigwig files


meta
```

```
  id condition
1  1     250ug
2  2     250ug
                                                                                                                                                                                clPlus
1 /Users/melinaklostermann/Documents/projects/iCLIP3/00_racoon_clip_processed_files/U2AF2_iCLIP3/bw/imb_koenig_2025_07_05_u2af65_250ug_rep1.R1.Aligned.sortedByCoord.out.duprm.plus.bw
2 /Users/melinaklostermann/Documents/projects/iCLIP3/00_racoon_clip_processed_files/U2AF2_iCLIP3/bw/imb_koenig_2025_07_06_u2af65_250ug_rep2.R1.Aligned.sortedByCoord.out.duprm.plus.bw
                                                                                                                                                                                clMinus
1 /Users/melinaklostermann/Documents/projects/iCLIP3/00_racoon_clip_processed_files/U2AF2_iCLIP3/bw/imb_koenig_2025_07_05_u2af65_250ug_rep1.R1.Aligned.sortedByCoord.out.duprm.minus.bw
2 /Users/melinaklostermann/Documents/projects/iCLIP3/00_racoon_clip_processed_files/U2AF2_iCLIP3/bw/imb_koenig_2025_07_06_u2af65_250ug_rep2.R1.Aligned.sortedByCoord.out.duprm.minus.bw
```

# 3 Make binding sites

## 3.1 Use automatic binding site generation

Show code

```
# --------------------
# run BindingSiteFinder in automatic mode
# --------------------

# make BSF objects
bds_object = BSFDataSetFromBigWig(ranges = peaks, 
                           meta = meta, 
                           silent =T)

# make inital BS allowing BSF to determine automatic parameters
bds_automatic = BSFind(bds_object, 
                 anno.genes = gns, 
                 anno.transcriptRegionList = regions, 
                 cutoff.geneWiseFilter = 0)


# save automatic binding sites
#saveRDS(bds_automatic, paste0(out, "bds_automatic.rds"))
#exportToBED(bds_automatic, con = "./binding_sites_automatic.bed")
```

## 3.2 Check automatic binding sites (and decide what parameters should be changed for final binding site definition)

Show code

```
# --------------------
# display paramteres choosen by automatic BSF
# --------------------

processingStepsFlowChart(bds_automatic)
```

Show code

```
ggsave(filename = paste0(out, "processing_steps_automatic_bs.pdf"))
```

# 4 Control (and optionally change) the binding site width

Show code

```
estimateBsWidthPlot(bds_automatic)
```

Show code

```
ggsave(filename = paste0(out, "estimate_binding_site_width.pdf"))
```

Show code

```
# --------------------
# Compare different binding site widths
# --------------------

# compute binding sites with different widths
bds1 <- makeBindingSites(object = bds_object, bsSize = 5)
bds2 <- makeBindingSites(object = bds_object, bsSize = 3)
bds3 <- makeBindingSites(object = bds_object, bsSize = 7)
bds4 <- makeBindingSites(object = bds_object, bsSize = 9)

# summarize in list
l = list(`automatic - bsSize = 5` = bds1, `close by - bsSize = 3` = bds2, 
         `close by - bsSize = 7` = bds3, `close by - bsSize = 9` = bds4)
# plot comparison
rangeCoveragePlot(l, width = 20, show.samples = F, subset.chromosome = "chr1")
```

Show code

```
ggsave(filename = paste0(out, "compare_binding_site_widths.pdf"))

# save bs for igv
exportToBED(bds2, con = paste0(out, "binding_sites_bs3.bed"))
exportToBED(bds3, con = paste0(out, "binding_sites_bs7.bed"))
exportToBED(bds4, con = paste0(out, "binding_sites_bs9.bed"))
```

### 4.0.1 Optional: change to a new bs width

Show code

```
bds_selected_width = BSFind(bds_object, 
                 anno.genes = gns, 
                 anno.transcriptRegionList = regions, 
                 bsSize = 7,
                 cutoff.geneWiseFilter = 0)


# Here we do not change to the bs width of 7 but continue using a width of 5.
bds_selected_width = BSFind(bds_object, 
                 anno.genes = gns, 
                 anno.transcriptRegionList = regions, 
                 bsSize = 7,
                 cutoff.geneWiseFilter = 0)
```

### 4.0.2 Optional: change genewise filter

Show code

```
bds_selected_width_genewise = BSFind(bds_object,
                     anno.genes = gns, 
                     anno.transcriptRegionList = regions, 
                     bsSize = 5,
                     cutoff.geneWiseFilter = 0.1)
```

## 4.1 Check reproducibility of binding sites and select cutoffs

Show code

```
reproducibilityFilterPlot(bds_selected_width_genewise)
```

Show code

```
ggsave(filename = paste0(out, "reproducibility_filter_plot.pdf"), width = 10, height = 5)

reproducibilitySamplesPlot(bds_selected_width_genewise)
```

Show code

```
pdf(paste0(out, "reproducibility_samples_plot.pdf"), width = 4, height = 3)
reproducibilitySamplesPlot(bds_selected_width_genewise)
dev.off()
```

```
svg 
  2
```

### 4.1.1 Optional: Change reproducibility support cutoff

Show code

```
# Make binding sites using new repro cutoff
bds_repro_stringent = BSFind(bds_object, 
                 anno.genes = gns, 
                 anno.transcriptRegionList = regions, 
                 bsSize = 5, # add the width you selected before
                 cutoff.geneWiseFilter = 0.1, # change genewise filter here 
                 repro.nReps = 2,
                 repro.cutoff = 0.1) # change cutoff here
# get numbers
bds_repro_stringent
```

```
Object of class BSFDataSet 
#N Ranges:  305,174 
Width ranges:  5 
#N Samples:  2 
#N Conditions:  1
```

Show code

```
# plot reproducibility support with changes settings
reproducibilityFilterPlot(bds_repro_stringent)
```

Show code

```
ggsave(filename = paste0(out, "reproducibility_filter_plot_repro_cut0.1.pdf"), width = 10, height = 5)

reproducibilitySamplesPlot(bds_repro_stringent)
```

Show code

```
pdf(paste0(out, "reproducibility_samples_plot_0.1.pdf"), width = 4, height = 3)
reproducibilitySamplesPlot(bds_repro_stringent)
dev.off()
```

```
svg 
  2
```

Show code

```
reproducibilityScatterPlot(bds_selected_width)
```

Show code

```
ggsave(filename = paste0(out, "reproducibility_scatter_plot_repro.pdf"), width = 5, height = 5)
reproducibilityScatterPlot(bds_repro_stringent)
```

Show code

```
ggsave(filename = paste0(out, "reproducibility_scatter_plot_after_repro_cut0.1.pdf"), width = 5, height = 5)
```

## 4.2 Export binding sites for IGV

Show code

```
bds_final <- bds_repro_stringent

# If you don't want to change the reproducibility cutoff, you can also directly export the binding sites with the automatic settings:
#bds_final <- bds_selected_width


exportToBED(bds_final, con = paste0(out, "binding_sites.bed"))
```

# 5 Characterise binding sites

Show code

```
# --------------------
# Bound genes
# --------------------
# the problem of overlapping genes
geneOverlapsPlot(bds_final)
```

Show code

```
# flag overlaps
# bds_alt_gene_asignment <- assignToGenes(bds_final, 
#                                         overlaps="keep",
#                                         anno.genes = gns,
#                                         )
# r <- getRanges(bds_alt_gene_asignment)
# r$dup <- duplicated(r$bsID) | duplicated(r$bsID, fromLast = TRUE)
# 
# r <- r[r$geneType == "protein_coding"]
# r <- r[order(r$score, decreasing = TRUE)]
# unique(r[r$dup == T ]$geneName)
```

## 5.1 Assign gene types

Show code

```
# resolving by hieracry
# ---------------------

# check gene types
unique(gns$gene_type)
```

```
 [1] "protein_coding"                     "transcribed_unprocessed_pseudogene"
 [3] "processed_pseudogene"               "lncRNA"                            
 [5] "transcribed_unitary_pseudogene"     "transcribed_processed_pseudogene"  
 [7] "unprocessed_pseudogene"             "IG_V_pseudogene"                   
 [9] "unitary_pseudogene"                 "TR_V_pseudogene"                   
[11] "IG_V_gene"                          "snRNA"                             
[13] "miRNA"                              "misc_RNA"                          
[15] "snoRNA"                             "rRNA_pseudogene"                   
[17] "rRNA"                               "vault_RNA"                         
[19] "TR_V_gene"                          "Mt_tRNA"                           
[21] "Mt_rRNA"                            "IG_C_gene"                         
[23] "IG_J_gene"                          "TR_J_gene"                         
[25] "TR_C_gene"                          "TR_J_pseudogene"                   
[27] "IG_D_gene"                          "ribozyme"                          
[29] "IG_C_pseudogene"                    "TR_D_gene"                         
[31] "TEC"                                "IG_J_pseudogene"                   
[33] "scaRNA"                             "translated_processed_pseudogene"   
[35] "artifact"                           "sRNA"                              
[37] "IG_pseudogene"
```

Show code

```
# decide which gene types are interesting
my_gene_types <- c("protein_coding", "lncRNA", "snRNA", "snoRNA", "miRNA", "rRNA", "misc_RNA", "tRNA")

# make a new column where all no interesting gene_types are summarized into "other" and pseudogenes are summarized into "pseudogenes"
gns <- as.data.frame(gns) %>% 
  dplyr::mutate(gene_type_plot = case_when(
    gene_type %in% my_gene_types ~ gene_type,
    grepl(gene_type, pattern = "pseudogene") ~ "pseudogene",
    TRUE ~ "other"
  )) %>%
  makeGRangesFromDataFrame(., keep.extra.columns = TRUE)

# set up your hierarcy from the most important type to the left to the least important type one the right
hierarchy <- c("protein_coding", "lncRNA", "snRNA", "snoRNA", "miRNA", "rRNA", "misc_RNA", "tRNA", "pseudogene", "other")
bds_alt_gene_asignment <- assignToGenes(bds_final, 
                                        overlaps = "hierarchy", 
                                        overlaps.rule = hierarchy, 
                                        anno.genes = gns,
                                        match.geneType = "gene_type_plot"
                                        )

targetGeneSpectrumPlot(bds_alt_gene_asignment, showNGroups = 20)
```

Show code

```
ggsave(filename = paste0(out, "target_gene_spectrum_plot.pdf"), width = 7, height = 5)
```

## 5.2 Assignment of bound transcipt regions

### 5.2.1 The problem: Multiple transcripts lead to ambiguty in binding site region

Show code

```
transcriptRegionOverlapsPlot(bds_final)
```

Show code

```
pdf(paste0(out, "transcript_region_overlaps_plot.pdf"), width = 7, height = 5)
transcriptRegionOverlapsPlot(bds_final)
dev.off()
```

```
svg 
  2
```

### 5.2.2 Resolved transcript regions

Show code

```
bds_final <- assignToTranscriptRegions(bds_final,
                                     overlaps = "hierarchy",
                                     overlaps.rule = c("INTRON", "UTR5", "UTR3", "CDS"),
                                     anno.transcriptRegionList = regions,
                                     )

transcriptRegionSpectrumPlot(bds_final, show.others = TRUE)
```

Show code

```
ggsave(paste0(out, "transcript_region_spectrum_plot.pdf"), width = 7, height = 5)
transcriptRegionSpectrumPlot(bds_final, normalize = TRUE)
```

Show code

```
ggsave(paste0(out, "transcript_region_spectrum_plot_normalised.pdf"), width = 7, height = 5)
#transcriptRegionSpectrumPlot(bds_final, normalize = TRUE, normalize.factor = "median")
```

# 6 Final binding sites

## 6.1 Depict the processing steps for the final binding site definition

Show code

```
# --------------------
# display paramteres choosen by automatic BSF
# --------------------
bds_final = BSFind(bds_object, 
                 anno.genes = gns, 
                 anno.transcriptRegionList = regions, 
                 bsSize = 5, # add the width you selected before
                 cutoff.geneWiseFilter = 0.1, # change genewise filter here 
                 repro.nReps = 2,
                 repro.cutoff = 0.1,
                 overlaps.geneAssignment = "hierarchy",
                 overlaps.rule.geneAssignment = hierarchy,
                 overlaps.TranscriptRegions = "hierarchy",
                 overlaps.rule.TranscriptRegions = c("INTRON", "UTR5", "UTR3", "CDS")
                 ) # change cutoff here


processingStepsFlowChart(bds_final)
```

Show code

```
ggsave(filename = paste0(out, "processing_steps_final_bs.pdf"))
```

## 6.2 Export binding sites

Show code

```
# Get list of all binding sites on target genes
exportTargetGenes(bds_final, paste0(out, "binding_sites_final_target_genes"))

# save binding sites 
exportToBED(bds_final, con = paste0(out, "binding_sites_final.bed"))
saveRDS(bds_final, paste0(out, "bds_final.rds"))
```

# 7 Session Info

Show code

```
sessionInfo()
```

```
R version 4.5.1 (2025-06-13)
Platform: aarch64-apple-darwin20
Running under: macOS Sequoia 15.6

Matrix products: default
BLAS:   /Library/Frameworks/R.framework/Versions/4.5-arm64/Resources/lib/libRblas.0.dylib 
LAPACK: /Library/Frameworks/R.framework/Versions/4.5-arm64/Resources/lib/libRlapack.dylib;  LAPACK version 3.12.1

locale:
[1] en_US.UTF-8/en_US.UTF-8/en_US.UTF-8/C/en_US.UTF-8/en_US.UTF-8

time zone: Europe/Berlin
tzcode source: internal

attached base packages:
[1] stats4    stats     graphics  grDevices utils     datasets  methods  
[8] base     

other attached packages:
 [1] BSgenome.Hsapiens.UCSC.hg38_1.4.5 BSgenome_1.78.0                  
 [3] BiocIO_1.20.0                     Biostrings_2.78.0                
 [5] XVector_0.50.0                    txdbmaker_1.6.0                  
 [7] GenomicFeatures_1.62.0            AnnotationDbi_1.72.0             
 [9] Biobase_2.70.0                    GenomeInfoDb_1.46.0              
[11] BindingSiteFinder_2.8.0           rtracklayer_1.70.0               
[13] GenomicRanges_1.62.0              Seqinfo_1.0.0                    
[15] IRanges_2.44.0                    S4Vectors_0.48.0                 
[17] BiocGenerics_0.56.0               generics_0.1.4                   
[19] lubridate_1.9.4                   forcats_1.0.1                    
[21] stringr_1.6.0                     dplyr_1.1.4                      
[23] purrr_1.2.0                       readr_2.1.5                      
[25] tidyr_1.3.1                       tibble_3.3.0                     
[27] ggplot2_4.0.0                     tidyverse_2.0.0                  
[29] knitr_1.50                       

loaded via a namespace (and not attached):
  [1] RColorBrewer_1.1-3          rstudioapi_0.17.1          
  [3] jsonlite_2.0.0              shape_1.4.6.1              
  [5] magrittr_2.0.4              magick_2.9.0               
  [7] farver_2.1.2                rmarkdown_2.30             
  [9] GlobalOptions_0.1.2         ragg_1.5.0                 
 [11] vctrs_0.6.5                 memoise_2.0.1              
 [13] Cairo_1.7-0                 Rsamtools_2.26.0           
 [15] RCurl_1.98-1.17             htmltools_0.5.8.1          
 [17] S4Arrays_1.10.0             progress_1.2.3             
 [19] distributional_0.5.0        curl_7.0.0                 
 [21] SparseArray_1.10.1          htmlwidgets_1.6.4          
 [23] plyr_1.8.9                  httr2_1.2.1                
 [25] cachem_1.1.0                GenomicAlignments_1.46.0   
 [27] lifecycle_1.0.4             iterators_1.0.14           
 [29] pkgconfig_2.0.3             Matrix_1.7-4               
 [31] R6_2.6.1                    fastmap_1.2.0              
 [33] MatrixGenerics_1.22.0       clue_0.3-66                
 [35] digest_0.6.37               GGally_2.4.0               
 [37] colorspace_2.1-2            textshaping_1.0.4          
 [39] RSQLite_2.4.3               filelock_1.0.3             
 [41] labeling_0.4.3              timechange_0.3.0           
 [43] httr_1.4.7                  polyclip_1.10-7            
 [45] abind_1.4-8                 compiler_4.5.1             
 [47] bit64_4.6.0-1               withr_3.0.2                
 [49] doParallel_1.0.17           S7_0.2.0                   
 [51] BiocParallel_1.44.0         viridis_0.6.5              
 [53] DBI_1.2.3                   ggstats_0.11.0             
 [55] ggforce_0.5.0               biomaRt_2.66.0             
 [57] MASS_7.3-65                 rappdirs_0.3.3             
 [59] DelayedArray_0.36.0         rjson_0.2.23               
 [61] tools_4.5.1                 glue_1.8.0                 
 [63] restfulr_0.0.16             grid_4.5.1                 
 [65] cluster_2.1.8.1             gtable_0.3.6               
 [67] tzdb_0.5.0                  hms_1.1.4                  
 [69] xml2_1.4.1                  foreach_1.5.2              
 [71] pillar_1.11.1               ggdist_3.3.3               
 [73] circlize_0.4.16             tweenr_2.0.3               
 [75] BiocFileCache_3.0.0         lattice_0.22-7             
 [77] bit_4.6.0                   tidyselect_1.2.1           
 [79] ComplexHeatmap_2.26.0       gridExtra_2.3              
 [81] SummarizedExperiment_1.40.0 svglite_2.2.2              
 [83] xfun_0.54                   matrixStats_1.5.0          
 [85] stringi_1.8.7               UCSC.utils_1.6.0           
 [87] yaml_2.3.10                 kableExtra_1.4.0           
 [89] evaluate_1.0.5              codetools_0.2-20           
 [91] cigarillo_1.0.0             cli_3.6.5                  
 [93] systemfonts_1.3.1           dichromat_2.0-0.1          
 [95] Rcpp_1.1.0                  dbplyr_2.5.1               
 [97] png_0.1-8                   XML_3.99-0.19              
 [99] parallel_4.5.1              blob_1.2.4                 
[101] prettyunits_1.2.0           bitops_1.0-9               
[103] viridisLite_0.4.2           scales_1.4.0               
[105] crayon_1.5.3                GetoptLong_1.0.5           
[107] rlang_1.1.6                 KEGGREST_1.50.0
```


##### Source Code

```
---
title: "Define binding sites example code"
author: "Melina Klostermann"
date: "`r format(Sys.time(), '%d %B, %Y')`"
format: 
  html:
    code-fold: true
    code-overflow: scroll
    code-summary: "Show code"
    code-tools: true
    code-line-numbers: true
    
    toc: true
    toc-depth: 3
    toc-location: left
    toc-expand: false
    number-sections: true
    
    theme: sandstone
    fontsize: 11pt
    linestretch: 1.5
    fig-format: svg
        
    cap-location: margin
    crossref:
      fig-title: Fig
    
    embed-resources: true
    link-external-newwindow: true
    smooth-scroll: true
    
    execute:
      echo: true
      warning: false
      cache: false

---
  
```{r setup, include=FALSE}
require("knitr")
knitr::opts_chunk$set(warning=FALSE, message=FALSE, cache=FALSE, cache.lazy = FALSE) #, fig.pos = "!H", out.extra = ""
```

```{r libraries, include=FALSE}
# ----------------------
# Load libraries
# ----------------------
library(knitr)
library(tidyverse)
library(purrr)

library(rtracklayer)
library(BindingSiteFinder)
library(GenomicRanges)
library(GenomeInfoDb)
library(txdbmaker)
library(Biostrings)
library(BSgenome.Hsapiens.UCSC.hg38)

# set an output folder
out <- "/Users/melinaklostermann/Documents/projects/iCLIP3/03_R_analyses/iCLIP2vs3_all_code/U2AF2/04_BS_def_example_code_250ug/out/"

set.seed(5)

```

# What is done here?

This is an exemplary code to define binding sites from racoon_clip outputs. Here the replicates of the for the U2AF2 iCLIP3 dataset with 250ug RNA are used. 

# Input files

```{r}
# ----------------------
# Get input from racoon_clip
# ----------------------
# pureclip files
pureclip_files <- "/Users/melinaklostermann/Documents/projects/iCLIP3/00_racoon_clip_processed_files/U2AF2_iCLIP3/pureclip_sites_u2af2_250ug.bed"


# bw files
bw_dir <- "/Users/melinaklostermann/Documents/projects/iCLIP3/00_racoon_clip_processed_files/U2AF2_iCLIP3"
bw.plus <- list.files(bw_dir, pattern = "u2af65_250ug.*\\plus.bw$", full.names = TRUE, recursive = TRUE)
bw.minus <- list.files(bw_dir, pattern = "u2af65_250ug.*\\minus.bw$", full.names = TRUE, recursive = TRUE)

```

```{r eval = FALSE}
# ----------------------
# Prepare annotation
# ----------------------
# Note: run only once to prepare annotation files for BSF and save them as rds files

# annotation file (download for example from GENCODE)
annoFile <- "/Users/melinaklostermann/Documents/projects/anno/GENCODEv49/gencode.v49.annotation.gtf.gz"
out_gns <- paste0(out, "gns.rds")
out_regions <- paste0(out, "regions.rds")

# prepare annotation for BSF
###########################
# Make annotation database from gff3 file
annoDb = txdbmaker::makeTxDbFromGFF(file = annoFile, format = "gtf")
annoInfo = rtracklayer::import(annoFile, format = "gtf")


# Get genes as GRanges
gns = genes(annoDb)
idx = match(gns$gene_id, annoInfo$gene_id)
meta = cbind(elementMetadata(gns),
             elementMetadata(annoInfo)[idx,])
meta = meta[,!duplicated(colnames(meta))]
elementMetadata(gns) = meta

saveRDS(gns, out_gns)


# Get regions as Granges
cdseq = cds(annoDb) 
intrns = unlist(intronsByTranscript(annoDb)) 
utrs3 = unlist(threeUTRsByTranscript(annoDb)) 
utrs5 = unlist(fiveUTRsByTranscript(annoDb)) 
regions = GRangesList(CDS = cdseq, INTRON = intrns, UTR3 = utrs3, UTR5 = utrs5)

saveRDS(regions, out_regions)

```


```{r}
# read prepared annotation
gns <- readRDS(paste0(out, "gns.rds"))
regions <- readRDS(paste0(out, "regions.rds"))
```


## Pureclip peaks

```{r}
#-----------------
# Peaks from pureclip
#-----------------
pureclip_file <- "/Users/melinaklostermann/Documents/projects/iCLIP3/00_racoon_clip_processed_files/U2AF2_iCLIP3/peaks/pureclip_sites_u2af2_250ug.bed"
# read pureclip peaks
peaks  = rtracklayer::import(con = pureclip_file, 
                             format = "BED", 
                             extraCols=c("additionalScores" = "character"))

# clean pureclip peaks columns
peaks$additionalScores = NULL
peaks$name = NULL

# optionally keep only standard chromosomes and drop chrM
peaks = keepStandardChromosomes(peaks, pruning.mode = "coarse") %>% 
                 dropSeqlevels(., "chrM", pruning.mode = "coarse" )

# check number of peaks
NROW(peaks)
```


## Metadata for BindingSiteFinder

```{r}
# --------------------
# make metadata for BindingSiteFinder
# --------------------

meta = data.frame(
  id = c(1,2), # give each sample a unique id
  condition = c("250ug", "250ug"), # add the condition for each sample (used for differential analysis), but one BSF object per condition needs to be used
  clPlus = bw.plus, # crosslinks plus strand bigwig files
  clMinus = bw.minus) # crosslinks minus strand bigwig files


meta
```

# Make binding sites

## Use automatic binding site generation

```{r}
# --------------------
# run BindingSiteFinder in automatic mode
# --------------------

# make BSF objects
bds_object = BSFDataSetFromBigWig(ranges = peaks, 
                           meta = meta, 
                           silent =T)

# make inital BS allowing BSF to determine automatic parameters
bds_automatic = BSFind(bds_object, 
                 anno.genes = gns, 
                 anno.transcriptRegionList = regions, 
                 cutoff.geneWiseFilter = 0)


# save automatic binding sites
#saveRDS(bds_automatic, paste0(out, "bds_automatic.rds"))
#exportToBED(bds_automatic, con = "./binding_sites_automatic.bed")
```

## Check automatic binding sites (and decide what parameters should be changed for final binding site definition)


```{r fig.width=5, fig.height=5}
# --------------------
# display paramteres choosen by automatic BSF
# --------------------

processingStepsFlowChart(bds_automatic)
ggsave(filename = paste0(out, "processing_steps_automatic_bs.pdf"))
```


# Control (and optionally change) the binding site width


```{r fig.width=5, fig.height=5}

estimateBsWidthPlot(bds_automatic)
ggsave(filename = paste0(out, "estimate_binding_site_width.pdf"))
```

```{r}
# --------------------
# Compare different binding site widths
# --------------------

# compute binding sites with different widths
bds1 <- makeBindingSites(object = bds_object, bsSize = 5)
bds2 <- makeBindingSites(object = bds_object, bsSize = 3)
bds3 <- makeBindingSites(object = bds_object, bsSize = 7)
bds4 <- makeBindingSites(object = bds_object, bsSize = 9)

# summarize in list
l = list(`automatic - bsSize = 5` = bds1, `close by - bsSize = 3` = bds2, 
         `close by - bsSize = 7` = bds3, `close by - bsSize = 9` = bds4)
# plot comparison
rangeCoveragePlot(l, width = 20, show.samples = F, subset.chromosome = "chr1")
ggsave(filename = paste0(out, "compare_binding_site_widths.pdf"))

# save bs for igv
exportToBED(bds2, con = paste0(out, "binding_sites_bs3.bed"))
exportToBED(bds3, con = paste0(out, "binding_sites_bs7.bed"))
exportToBED(bds4, con = paste0(out, "binding_sites_bs9.bed"))

```

### Optional: change to a new bs width

```{r}
bds_selected_width = BSFind(bds_object, 
                 anno.genes = gns, 
                 anno.transcriptRegionList = regions, 
                 bsSize = 7,
                 cutoff.geneWiseFilter = 0)


# Here we do not change to the bs width of 7 but continue using a width of 5.
bds_selected_width = BSFind(bds_object, 
                 anno.genes = gns, 
                 anno.transcriptRegionList = regions, 
                 bsSize = 7,
                 cutoff.geneWiseFilter = 0)

```


### Optional: change genewise filter

```{r}
bds_selected_width_genewise = BSFind(bds_object,
                     anno.genes = gns, 
                     anno.transcriptRegionList = regions, 
                     bsSize = 5,
                     cutoff.geneWiseFilter = 0.1)


```


## Check reproducibility of binding sites and select cutoffs

```{r fig.width=5, fig.height=5}

reproducibilityFilterPlot(bds_selected_width_genewise)
ggsave(filename = paste0(out, "reproducibility_filter_plot.pdf"), width = 10, height = 5)

reproducibilitySamplesPlot(bds_selected_width_genewise)

pdf(paste0(out, "reproducibility_samples_plot.pdf"), width = 4, height = 3)
reproducibilitySamplesPlot(bds_selected_width_genewise)
dev.off()

```

### Optional: Change reproducibility support cutoff

```{r}
# Make binding sites using new repro cutoff
bds_repro_stringent = BSFind(bds_object, 
                 anno.genes = gns, 
                 anno.transcriptRegionList = regions, 
                 bsSize = 5, # add the width you selected before
                 cutoff.geneWiseFilter = 0.1, # change genewise filter here 
                 repro.nReps = 2,
                 repro.cutoff = 0.1) # change cutoff here
# get numbers
bds_repro_stringent

# plot reproducibility support with changes settings
reproducibilityFilterPlot(bds_repro_stringent)
ggsave(filename = paste0(out, "reproducibility_filter_plot_repro_cut0.1.pdf"), width = 10, height = 5)

reproducibilitySamplesPlot(bds_repro_stringent)

pdf(paste0(out, "reproducibility_samples_plot_0.1.pdf"), width = 4, height = 3)
reproducibilitySamplesPlot(bds_repro_stringent)
dev.off()
```


```{r}
reproducibilityScatterPlot(bds_selected_width)
ggsave(filename = paste0(out, "reproducibility_scatter_plot_repro.pdf"), width = 5, height = 5)
reproducibilityScatterPlot(bds_repro_stringent)
ggsave(filename = paste0(out, "reproducibility_scatter_plot_after_repro_cut0.1.pdf"), width = 5, height = 5)

```

## Export binding sites for IGV
```{r}
bds_final <- bds_repro_stringent

# If you don't want to change the reproducibility cutoff, you can also directly export the binding sites with the automatic settings:
#bds_final <- bds_selected_width


exportToBED(bds_final, con = paste0(out, "binding_sites.bed"))
```


# Characterise binding sites

```{r}
# --------------------
# Bound genes
# --------------------
# the problem of overlapping genes
geneOverlapsPlot(bds_final)

# flag overlaps
# bds_alt_gene_asignment <- assignToGenes(bds_final, 
#                                         overlaps="keep",
#                                         anno.genes = gns,
#                                         )
# r <- getRanges(bds_alt_gene_asignment)
# r$dup <- duplicated(r$bsID) | duplicated(r$bsID, fromLast = TRUE)
# 
# r <- r[r$geneType == "protein_coding"]
# r <- r[order(r$score, decreasing = TRUE)]
# unique(r[r$dup == T ]$geneName)
```

## Assign gene types

```{r}
# resolving by hieracry
# ---------------------

# check gene types
unique(gns$gene_type)
# decide which gene types are interesting
my_gene_types <- c("protein_coding", "lncRNA", "snRNA", "snoRNA", "miRNA", "rRNA", "misc_RNA", "tRNA")

# make a new column where all no interesting gene_types are summarized into "other" and pseudogenes are summarized into "pseudogenes"
gns <- as.data.frame(gns) %>% 
  dplyr::mutate(gene_type_plot = case_when(
    gene_type %in% my_gene_types ~ gene_type,
    grepl(gene_type, pattern = "pseudogene") ~ "pseudogene",
    TRUE ~ "other"
  )) %>%
  makeGRangesFromDataFrame(., keep.extra.columns = TRUE)

# set up your hierarcy from the most important type to the left to the least important type one the right
hierarchy <- c("protein_coding", "lncRNA", "snRNA", "snoRNA", "miRNA", "rRNA", "misc_RNA", "tRNA", "pseudogene", "other")
bds_alt_gene_asignment <- assignToGenes(bds_final, 
                                        overlaps = "hierarchy", 
                                        overlaps.rule = hierarchy, 
                                        anno.genes = gns,
                                        match.geneType = "gene_type_plot"
                                        )

targetGeneSpectrumPlot(bds_alt_gene_asignment, showNGroups = 20)
ggsave(filename = paste0(out, "target_gene_spectrum_plot.pdf"), width = 7, height = 5)

```

## Assignment of bound transcipt regions

### The problem: Multiple transcripts lead to ambiguty in binding site region

```{r}
transcriptRegionOverlapsPlot(bds_final)

pdf(paste0(out, "transcript_region_overlaps_plot.pdf"), width = 7, height = 5)
transcriptRegionOverlapsPlot(bds_final)
dev.off()

```

### Resolved transcript regions

```{r}
bds_final <- assignToTranscriptRegions(bds_final,
                                     overlaps = "hierarchy",
                                     overlaps.rule = c("INTRON", "UTR5", "UTR3", "CDS"),
                                     anno.transcriptRegionList = regions,
                                     )

transcriptRegionSpectrumPlot(bds_final, show.others = TRUE)
ggsave(paste0(out, "transcript_region_spectrum_plot.pdf"), width = 7, height = 5)
transcriptRegionSpectrumPlot(bds_final, normalize = TRUE)
ggsave(paste0(out, "transcript_region_spectrum_plot_normalised.pdf"), width = 7, height = 5)
#transcriptRegionSpectrumPlot(bds_final, normalize = TRUE, normalize.factor = "median")

```

# Final binding sites

## Depict the processing steps for the final binding site definition


```{r fig.width=5, fig.height=5}
# --------------------
# display paramteres choosen by automatic BSF
# --------------------
bds_final = BSFind(bds_object, 
                 anno.genes = gns, 
                 anno.transcriptRegionList = regions, 
                 bsSize = 5, # add the width you selected before
                 cutoff.geneWiseFilter = 0.1, # change genewise filter here 
                 repro.nReps = 2,
                 repro.cutoff = 0.1,
                 overlaps.geneAssignment = "hierarchy",
                 overlaps.rule.geneAssignment = hierarchy,
                 overlaps.TranscriptRegions = "hierarchy",
                 overlaps.rule.TranscriptRegions = c("INTRON", "UTR5", "UTR3", "CDS")
                 ) # change cutoff here


processingStepsFlowChart(bds_final)
ggsave(filename = paste0(out, "processing_steps_final_bs.pdf"))
```


## Export binding sites

```{r}
# Get list of all binding sites on target genes
exportTargetGenes(bds_final, paste0(out, "binding_sites_final_target_genes"))

# save binding sites 
exportToBED(bds_final, con = paste0(out, "binding_sites_final.bed"))
saveRDS(bds_final, paste0(out, "bds_final.rds"))
```


# Session Info

```{r}
sessionInfo()
```
```
