# Supplementary material for: Isotope-free mapping of protein-RNA interactions at single-nucleotide resolution by iCLIP3
Source: STAR Protoc. 2026 Jul 17;7(3):104704. doi: 10.1016/j.xpro.2026.104704 (PMC13400664; doi:10.1016/j.xpro.2026.104704)
Supplement: Data S3. BindingSiteFinder analysis plots [file mmc4.zip › Data S3. BindingSiteFinder analysis plots.html]

Define binding sites for all iCLIP3 U2AF2 data sets


## Table of contents

- 1 What is done here?
- 2 Input files
  - 2.1 Preparing the annotation files for BindingSiteFinder
  - 2.2 Pureclip peaks
    - 2.2.1 Number of pureclip peaks
  - 2.3 Crosslinks
- 3 Make binding sites
- 4 Check quality binding sites
  - 4.1 100ug
  - 4.2 250ug
  - 4.3 40ug
- 5 Binding site characteristics
  - 5.1 100ug
  - 5.2 250ug
  - 5.3 40ug
- 6 Session info

# Define binding sites for all iCLIP3 U2AF2 data sets

 Code

- Show All Code
- Hide All Code
- ---
- View Source

Author

Melina Klostermann

Published

February 27, 2026

# 1 What is done here?

This code performs the same bindingSiteAnalysis that is shown exemplarily for the U2AF2 250µg sample in Protocol 4 - Defining binding sites with BindingSiteFinder for all 3 iCLIP3 samples (250µg, 100µg, 40µg).

- The resulting numbers of binding sites are used in Figure 8A.
- The resulting binding sites are used for the corrleation matrix in Figure 8B and the metaprofile in Figure 8C.

# 2 Input files

The inputs are:

- Pureclip peaks for each iCLIP3 sample (250µg, 100µg, 40µg)
- Bigwig files for each iCLIP3 replicate of each sample (250µg, 100µg, 40µg) and each strand (plus, minus)
- Annotation files for the binding site definition.

Show code

```
# pureclip files
pureclip_files <- list.files("/Users/melinaklostermann/Documents/projects/iCLIP3/00_racoon_clip_processed_files/U2AF2_iCLIP3/peaks", pattern = "ug.bed", full.names = TRUE)

names(pureclip_files) <- list.files("/Users/melinaklostermann/Documents/projects/iCLIP3/00_racoon_clip_processed_files/U2AF2_iCLIP3/peaks", pattern = "ug.bed", full.names = FALSE) %>% 
  str_remove_all("pureclip_sites_u2af2_") %>%
  str_remove_all(".bed") 

exp_names <- names(pureclip_files)


# bw files
bw_dir <- "/Users/melinaklostermann/Documents/projects/iCLIP3/00_racoon_clip_processed_files/U2AF2_iCLIP3/bw"
bw_files_all <- list.files(bw_dir, pattern = "imb.*\\ug.*\\.bw$", full.names = TRUE, recursive = TRUE)

# Extract the category name directly using vectorized matching
get_category <- function(x) {
  cat_match <- sapply(exp_names, function(cat) grepl(cat, x, ignore.case = TRUE))
  exp_names[max.col(cat_match, ties.method = "first")] %>%
    replace(is.infinite(.), NA)  # handle no-match cases
}

# Build dataframe
bw_df <- data.frame(
  file = bw_files_all,
  category = get_category(bw_files_all),
  strand = ifelse(grepl("plus\\.bw$", bw_files_all), "p",
                  ifelse(grepl("minus\\.bw$", bw_files_all), "m", NA)),
  stringsAsFactors = FALSE
)

# Drop unmatched entries
bw_df <- subset(bw_df, !is.na(category) & !is.na(strand))

# Use split() to create the nested list
bw_list_split <- split(bw_df, bw_df$category)

bw_files <- lapply(bw_list_split, function(df) {
  split(df$file, df$strand)[c("p", "m")]  # ensures order p, m
})
```

## 2.1 Preparing the annotation files for BindingSiteFinder

Show code

```
anno_path <- "/Users/melinaklostermann/Documents/projects/anno/GENCODEv49/gencode.v49.annotation.gtf.gz"
prepare_anno_for_bsf(anno_path, paste0(out, "gns.rds"), paste0(out, "regions.rds"))
```

Show code

```
gns <- readRDS(paste0(out, "gns.rds"))
regions <- readRDS(paste0(out, "regions.rds"))
```

## 2.2 Pureclip peaks

Show code

```
# Peaks from pureclip
peaks  = sapply(pureclip_files, function(x) prepare_pureclip_for_BSF(x))
peaks = sapply(peaks, function(x) GenomeInfoDb::keepStandardChromosomes(x, pruning.mode = "coarse") %>% 
                 GenomeInfoDb::dropSeqlevels(., "chrM", pruning.mode = "coarse" ))
```

### 2.2.1 Number of pureclip peaks

Show code

```
n_peaks <- sapply(names(peaks), function(x){
  cat(x)
  NROW(peaks[[x]])}) %>% as.data.frame()
```

```
100ug250ug40ug
```

Show code

```
colnames(n_peaks) <- c("n_peaks")

kable(n_peaks)
```

|  | n\_peaks |
| --- | --- |
| 100ug | 1907770 |
| 250ug | 1830102 |
| 40ug | 1754053 |

## 2.3 Crosslinks

Show code

```
# --------------------
# make meta
# --------------------

meta = list()

for(i in 1:length(exp_names)){
  bw = bw_files[[i]]
  n = length(bw$p)
  
 meta[[i]] = data.frame(
  id = c(1:n),
  condition = factor(rep(exp_names[i], n)), # add option for multiple groups from sample file
  clPlus = bw$p, 
  clMinus = bw$m)
}
names(meta) = exp_names


lapply(meta, function(x) kable(x))
```

```
$`100ug`


| id|condition |clPlus                                                                                                                                                                               |clMinus                                                                                                                                                                               |
|--:|:---------|:------------------------------------------------------------------------------------------------------------------------------------------------------------------------------------|:-------------------------------------------------------------------------------------------------------------------------------------------------------------------------------------|
|  1|100ug     |/Users/melinaklostermann/Documents/projects/iCLIP3/00_racoon_clip_processed_files/U2AF2_iCLIP3/bw/imb_koenig_2025_07_03_u2af65_100ug_rep1.R1.Aligned.sortedByCoord.out.duprm.plus.bw |/Users/melinaklostermann/Documents/projects/iCLIP3/00_racoon_clip_processed_files/U2AF2_iCLIP3/bw/imb_koenig_2025_07_03_u2af65_100ug_rep1.R1.Aligned.sortedByCoord.out.duprm.minus.bw |
|  2|100ug     |/Users/melinaklostermann/Documents/projects/iCLIP3/00_racoon_clip_processed_files/U2AF2_iCLIP3/bw/imb_koenig_2025_07_04_u2af65_100ug_rep2.R1.Aligned.sortedByCoord.out.duprm.plus.bw |/Users/melinaklostermann/Documents/projects/iCLIP3/00_racoon_clip_processed_files/U2AF2_iCLIP3/bw/imb_koenig_2025_07_04_u2af65_100ug_rep2.R1.Aligned.sortedByCoord.out.duprm.minus.bw |

$`250ug`


| id|condition |clPlus                                                                                                                                                                               |clMinus                                                                                                                                                                               |
|--:|:---------|:------------------------------------------------------------------------------------------------------------------------------------------------------------------------------------|:-------------------------------------------------------------------------------------------------------------------------------------------------------------------------------------|
|  1|250ug     |/Users/melinaklostermann/Documents/projects/iCLIP3/00_racoon_clip_processed_files/U2AF2_iCLIP3/bw/imb_koenig_2025_07_05_u2af65_250ug_rep1.R1.Aligned.sortedByCoord.out.duprm.plus.bw |/Users/melinaklostermann/Documents/projects/iCLIP3/00_racoon_clip_processed_files/U2AF2_iCLIP3/bw/imb_koenig_2025_07_05_u2af65_250ug_rep1.R1.Aligned.sortedByCoord.out.duprm.minus.bw |
|  2|250ug     |/Users/melinaklostermann/Documents/projects/iCLIP3/00_racoon_clip_processed_files/U2AF2_iCLIP3/bw/imb_koenig_2025_07_06_u2af65_250ug_rep2.R1.Aligned.sortedByCoord.out.duprm.plus.bw |/Users/melinaklostermann/Documents/projects/iCLIP3/00_racoon_clip_processed_files/U2AF2_iCLIP3/bw/imb_koenig_2025_07_06_u2af65_250ug_rep2.R1.Aligned.sortedByCoord.out.duprm.minus.bw |

$`40ug`


| id|condition |clPlus                                                                                                                                                                              |clMinus                                                                                                                                                                              |
|--:|:---------|:-----------------------------------------------------------------------------------------------------------------------------------------------------------------------------------|:------------------------------------------------------------------------------------------------------------------------------------------------------------------------------------|
|  1|40ug      |/Users/melinaklostermann/Documents/projects/iCLIP3/00_racoon_clip_processed_files/U2AF2_iCLIP3/bw/imb_koenig_2025_07_01_u2af65_40ug_rep1.R1.Aligned.sortedByCoord.out.duprm.plus.bw |/Users/melinaklostermann/Documents/projects/iCLIP3/00_racoon_clip_processed_files/U2AF2_iCLIP3/bw/imb_koenig_2025_07_01_u2af65_40ug_rep1.R1.Aligned.sortedByCoord.out.duprm.minus.bw |
|  2|40ug      |/Users/melinaklostermann/Documents/projects/iCLIP3/00_racoon_clip_processed_files/U2AF2_iCLIP3/bw/imb_koenig_2025_07_02_u2af65_40ug_rep2.R1.Aligned.sortedByCoord.out.duprm.plus.bw |/Users/melinaklostermann/Documents/projects/iCLIP3/00_racoon_clip_processed_files/U2AF2_iCLIP3/bw/imb_koenig_2025_07_02_u2af65_40ug_rep2.R1.Aligned.sortedByCoord.out.duprm.minus.bw |
```

# 3 Make binding sites

Show code

```
# --------------------
# run BindingSiteFinder 
# --------------------

# make BSF objects
bds = mapply(x = peaks, y = meta, function(x,y) BSFDataSetFromBigWig(ranges = x, meta = y, silent =T))

# set hieracries
gene_hierarcy <- c("protein_coding", "lncRNA", "snRNA", "snoRNA", "miRNA", "rRNA", "misc_RNA", "tRNA", "pseudogene")
transcript_region_hierarcy <- c("INTRON", "UTR5", "UTR3", "CDS")

#--------------------------------
# make inital BS repro 0.05, geneWiseFilter = 0
#--------------------------------
bds[1:3] = lapply(bds[1:3], function(x) BSFind(x, 
                                     anno.genes = gns, 
                                     anno.transcriptRegionList = regions, 
                                     bsSize = 5,
                                     cutoff.geneWiseFilter = 0.1,
                                     repro.nReps = 2,
                                     repro.cutoff = 0.1,
                                     overlaps.geneAssignment = "hierarchy",
                                     overlaps.rule.geneAssignment = gene_hierarcy,
                                     overlaps.TranscriptRegions = "hierarchy",
                                     overlaps.rule.TranscriptRegions = transcript_region_hierarcy
                                     ))


saveRDS(bds, paste0(out, "bds.rds"))

lapply(names(bds), function(x) {
  exportToBED(bds[[x]], paste0(out, x, "_binding_sites.bed"))
  #saveRDS(getRanges(bds[[x]]), paste0(out, x, "_binding_sites.rds"))
  })
```

Loops over the 3 samples and makes binding sites with the same parameters as for the 250µg sample in Protocol 4 - Defining binding sites with BindingSiteFinder. The paramerters are:

- bsSize = 5
- cutoff.geneWiseFilter = 0.1
- repro.nReps = 2
- repro.cutoff = 0.1
- overlaps.geneAssignment = “hierarchy”
- overlaps.rule.geneAssignment = gene\_hierarcy
- overlaps.TranscriptRegions = “hierarchy”
- overlaps.rule.TranscriptRegions = transcript\_region\_hierarcy

The gene hierarchy is set to: “protein\_coding” > “lncRNA” > “snRNA” > “snoRNA” > “miRNA” > “rRNA” > “misc\_RNA” > “tRNA” > “pseudogene”.

The transcript region hierarchy is set to: “INTRON” > “UTR5” > “UTR3” > “CDS”.

# 4 Check quality binding sites

The following shows the quality control plots for the binding sites for all 3 samples.

Show code

```
bds <- readRDS(paste0(out, "bds.rds"))

for(i in 1:length(bds[1:3])){
  cat("##", names(bds)[i], "\n")
  print(quick_BSF_QC(bds[[i]], estimate.bs.width = F))
  cat("\n\n")
}
```

## 4.1 100ug

[1] “” [1] “”

## 4.2 250ug

[1] “” [1] “”

## 4.3 40ug

[1] “” [1] “”

# 5 Binding site characteristics

The following shows the binding site characteristics for all 3 samples.

Show code

```
for(i in 1:length(bds[1:3])){
  cat("##", names(bds)[i], "\n")
   print(quick_BSF_characterise(bds[[i]]))
   cat("\n\n")
}
```

## 5.1 100ug

[1] “” [1] “”

## 5.2 250ug

[1] “” [1] “”

## 5.3 40ug

[1] “” [1] “”

# 6 Session info

Here you can see the exact versions of the packages used for the analysis.

Show code

```
sessionInfo()
```

```
R version 4.5.1 (2025-06-13)
Platform: aarch64-apple-darwin20
Running under: macOS Sequoia 15.6

Matrix products: default
BLAS:   /Library/Frameworks/R.framework/Versions/4.5-arm64/Resources/lib/libRblas.0.dylib 
LAPACK: /Library/Frameworks/R.framework/Versions/4.5-arm64/Resources/lib/libRlapack.dylib;  LAPACK version 3.12.1

locale:
[1] en_US.UTF-8/en_US.UTF-8/en_US.UTF-8/C/en_US.UTF-8/en_US.UTF-8

time zone: Europe/Berlin
tzcode source: internal

attached base packages:
[1] stats4    stats     graphics  grDevices utils     datasets  methods  
[8] base     

other attached packages:
 [1] GenomeInfoDb_1.46.0     BindingSiteFinder_2.8.0 lubridate_1.9.4        
 [4] forcats_1.0.1           stringr_1.6.0           readr_2.1.5            
 [7] tidyr_1.3.1             tibble_3.3.0            tidyverse_2.0.0        
[10] ggplot2_4.0.0           rtracklayer_1.70.0      GenomicRanges_1.62.0   
[13] Seqinfo_1.0.0           IRanges_2.44.0          S4Vectors_0.48.0       
[16] BiocGenerics_0.56.0     generics_0.1.4          purrr_1.2.0            
[19] dplyr_1.1.4             knitr_1.50             

loaded via a namespace (and not attached):
  [1] DBI_1.2.3                   bitops_1.0-9               
  [3] gridExtra_2.3               rlang_1.1.6                
  [5] magrittr_2.0.4              clue_0.3-66                
  [7] GetoptLong_1.0.5            matrixStats_1.5.0          
  [9] compiler_4.5.1              RSQLite_2.4.3              
 [11] GenomicFeatures_1.62.0      systemfonts_1.3.1          
 [13] png_0.1-8                   vctrs_0.6.5                
 [15] pkgconfig_2.0.3             shape_1.4.6.1              
 [17] crayon_1.5.3                fastmap_1.2.0              
 [19] magick_2.9.0                XVector_0.50.0             
 [21] labeling_0.4.3              Rsamtools_2.26.0           
 [23] rmarkdown_2.30              tzdb_0.5.0                 
 [25] UCSC.utils_1.6.0            bit_4.6.0                  
 [27] xfun_0.54                   cachem_1.1.0               
 [29] cigarillo_1.0.0             jsonlite_2.0.0             
 [31] blob_1.2.4                  DelayedArray_0.36.0        
 [33] tweenr_2.0.3                BiocParallel_1.44.0        
 [35] parallel_4.5.1              cluster_2.1.8.1            
 [37] R6_2.6.1                    stringi_1.8.7              
 [39] RColorBrewer_1.1-3          GGally_2.4.0               
 [41] Rcpp_1.1.0                  SummarizedExperiment_1.40.0
 [43] iterators_1.0.14            Matrix_1.7-4               
 [45] timechange_0.3.0            tidyselect_1.2.1           
 [47] viridis_0.6.5               rstudioapi_0.17.1          
 [49] dichromat_2.0-0.1           abind_1.4-8                
 [51] yaml_2.3.10                 doParallel_1.0.17          
 [53] codetools_0.2-20            curl_7.0.0                 
 [55] plyr_1.8.9                  lattice_0.22-7             
 [57] Biobase_2.70.0              withr_3.0.2                
 [59] KEGGREST_1.50.0             S7_0.2.0                   
 [61] evaluate_1.0.5              ggstats_0.11.0             
 [63] polyclip_1.10-7             xml2_1.4.1                 
 [65] ggdist_3.3.3                circlize_0.4.16            
 [67] Biostrings_2.78.0           pillar_1.11.1              
 [69] MatrixGenerics_1.22.0       foreach_1.5.2              
 [71] distributional_0.5.0        RCurl_1.98-1.17            
 [73] hms_1.1.4                   scales_1.4.0               
 [75] glue_1.8.0                  tools_4.5.1                
 [77] BiocIO_1.20.0               GenomicAlignments_1.46.0   
 [79] XML_3.99-0.19               Cairo_1.7-0                
 [81] grid_4.5.1                  AnnotationDbi_1.72.0       
 [83] colorspace_2.1-2            ggforce_0.5.0              
 [85] restfulr_0.0.16             cli_3.6.5                  
 [87] textshaping_1.0.4           kableExtra_1.4.0           
 [89] viridisLite_0.4.2           S4Arrays_1.10.0            
 [91] svglite_2.2.2               ComplexHeatmap_2.26.0      
 [93] gtable_0.3.6                digest_0.6.37              
 [95] SparseArray_1.10.1          rjson_0.2.23               
 [97] htmlwidgets_1.6.4           farver_2.1.2               
 [99] memoise_2.0.1               htmltools_0.5.8.1          
[101] lifecycle_1.0.4             httr_1.4.7                 
[103] GlobalOptions_0.1.2         bit64_4.6.0-1              
[105] MASS_7.3-65
```


##### Source Code

```
---
title: "Define binding sites for all iCLIP3 U2AF2 data sets"
author: "Melina Klostermann"
date: "`r format(Sys.time(), '%d %B, %Y')`"
format: 
  html:
    code-fold: true
    code-overflow: scroll
    code-summary: "Show code"
    code-tools: true
    code-line-numbers: true
    
    toc: true
    toc-depth: 3
    toc-location: left
    toc-expand: false
    number-sections: true
    
    theme: sandstone
    fontsize: 11pt
    linestretch: 1.5
    fig-format: svg
        
    cap-location: margin
    crossref:
      fig-title: Fig
    
    embed-resources: true
    link-external-newwindow: true
    smooth-scroll: true
    
    execute:
      echo: true
      warning: false
      cache: false

---
  
```{r setup, include=FALSE}
require("knitr")
knitr::opts_chunk$set(warning=FALSE, message=FALSE, cache=FALSE, cache.lazy = FALSE) #, fig.pos = "!H", out.extra = ""
```

```{r libraries, include=FALSE}
library(knitr)
library(dplyr)
library(purrr)
library(rtracklayer)
library(ggplot2)
library(tidyverse)
library(BindingSiteFinder)
library(GenomicRanges)
library(GenomeInfoDb)
source("/Users/melinaklostermann/Documents/projects/R_general_functions/BFS_helper_functions.R")


out <- "/Users/melinaklostermann/Documents/projects/iCLIP3/03_R_analyses/iCLIP2vs3_all_code/U2AF2/03_1_iCLIP3_full_binding_sites_repro01_gwf01_PRI/out/"

set.seed(5)

```

# What is done here?

This code performs the same bindingSiteAnalysis that is shown exemplarily for the U2AF2 250µg sample in Protocol 4 - Defining binding sites with BindingSiteFinder for all 3 iCLIP3 samples (250µg, 100µg, 40µg). 

- The resulting numbers of binding sites are used in Figure 8A. 
- The resulting binding sites are used for the corrleation matrix in Figure 8B and the metaprofile in Figure 8C. 

# Input files

The inputs are:

- Pureclip peaks for each iCLIP3 sample (250µg, 100µg, 40µg)
- Bigwig files for each iCLIP3 replicate of each sample (250µg, 100µg, 40µg) and each strand (plus, minus)
- Annotation files for the binding site definition.

```{r}
# pureclip files
pureclip_files <- list.files("/Users/melinaklostermann/Documents/projects/iCLIP3/00_racoon_clip_processed_files/U2AF2_iCLIP3/peaks", pattern = "ug.bed", full.names = TRUE)

names(pureclip_files) <- list.files("/Users/melinaklostermann/Documents/projects/iCLIP3/00_racoon_clip_processed_files/U2AF2_iCLIP3/peaks", pattern = "ug.bed", full.names = FALSE) %>% 
  str_remove_all("pureclip_sites_u2af2_") %>%
  str_remove_all(".bed") 

exp_names <- names(pureclip_files)


# bw files
bw_dir <- "/Users/melinaklostermann/Documents/projects/iCLIP3/00_racoon_clip_processed_files/U2AF2_iCLIP3/bw"
bw_files_all <- list.files(bw_dir, pattern = "imb.*\\ug.*\\.bw$", full.names = TRUE, recursive = TRUE)

# Extract the category name directly using vectorized matching
get_category <- function(x) {
  cat_match <- sapply(exp_names, function(cat) grepl(cat, x, ignore.case = TRUE))
  exp_names[max.col(cat_match, ties.method = "first")] %>%
    replace(is.infinite(.), NA)  # handle no-match cases
}

# Build dataframe
bw_df <- data.frame(
  file = bw_files_all,
  category = get_category(bw_files_all),
  strand = ifelse(grepl("plus\\.bw$", bw_files_all), "p",
                  ifelse(grepl("minus\\.bw$", bw_files_all), "m", NA)),
  stringsAsFactors = FALSE
)

# Drop unmatched entries
bw_df <- subset(bw_df, !is.na(category) & !is.na(strand))

# Use split() to create the nested list
bw_list_split <- split(bw_df, bw_df$category)

bw_files <- lapply(bw_list_split, function(df) {
  split(df$file, df$strand)[c("p", "m")]  # ensures order p, m
})


```

## Preparing the annotation files for BindingSiteFinder
```{r eval= F}
anno_path <- "/Users/melinaklostermann/Documents/projects/anno/GENCODEv49/gencode.v49.annotation.gtf.gz"
prepare_anno_for_bsf(anno_path, paste0(out, "gns.rds"), paste0(out, "regions.rds"))

```

```{r}
gns <- readRDS(paste0(out, "gns.rds"))
regions <- readRDS(paste0(out, "regions.rds"))

```


## Pureclip peaks

```{r}
# Peaks from pureclip
peaks  = sapply(pureclip_files, function(x) prepare_pureclip_for_BSF(x))
peaks = sapply(peaks, function(x) GenomeInfoDb::keepStandardChromosomes(x, pruning.mode = "coarse") %>% 
                 GenomeInfoDb::dropSeqlevels(., "chrM", pruning.mode = "coarse" ))

```

### Number of pureclip peaks

```{r}
n_peaks <- sapply(names(peaks), function(x){
  cat(x)
  NROW(peaks[[x]])}) %>% as.data.frame()

colnames(n_peaks) <- c("n_peaks")

kable(n_peaks)

```

## Crosslinks

```{r}
# --------------------
# make meta
# --------------------

meta = list()

for(i in 1:length(exp_names)){
  bw = bw_files[[i]]
  n = length(bw$p)
  
 meta[[i]] = data.frame(
  id = c(1:n),
  condition = factor(rep(exp_names[i], n)), # add option for multiple groups from sample file
  clPlus = bw$p, 
  clMinus = bw$m)
}
names(meta) = exp_names


lapply(meta, function(x) kable(x))
```

# Make binding sites 

```{r eval=F}
# --------------------
# run BindingSiteFinder 
# --------------------

# make BSF objects
bds = mapply(x = peaks, y = meta, function(x,y) BSFDataSetFromBigWig(ranges = x, meta = y, silent =T))

# set hieracries
gene_hierarcy <- c("protein_coding", "lncRNA", "snRNA", "snoRNA", "miRNA", "rRNA", "misc_RNA", "tRNA", "pseudogene")
transcript_region_hierarcy <- c("INTRON", "UTR5", "UTR3", "CDS")

#--------------------------------
# make inital BS repro 0.05, geneWiseFilter = 0
#--------------------------------
bds[1:3] = lapply(bds[1:3], function(x) BSFind(x, 
                                     anno.genes = gns, 
                                     anno.transcriptRegionList = regions, 
                                     bsSize = 5,
                                     cutoff.geneWiseFilter = 0.1,
                                     repro.nReps = 2,
                                     repro.cutoff = 0.1,
                                     overlaps.geneAssignment = "hierarchy",
                                     overlaps.rule.geneAssignment = gene_hierarcy,
                                     overlaps.TranscriptRegions = "hierarchy",
                                     overlaps.rule.TranscriptRegions = transcript_region_hierarcy
                                     ))


saveRDS(bds, paste0(out, "bds.rds"))

lapply(names(bds), function(x) {
  exportToBED(bds[[x]], paste0(out, x, "_binding_sites.bed"))
  #saveRDS(getRanges(bds[[x]]), paste0(out, x, "_binding_sites.rds"))
  })

```

Loops over the 3 samples and makes binding sites with the same parameters as for the 250µg sample in Protocol 4 - Defining binding sites with BindingSiteFinder. The paramerters are:

- bsSize = 5
- cutoff.geneWiseFilter = 0.1
- repro.nReps = 2
- repro.cutoff = 0.1
- overlaps.geneAssignment = "hierarchy"
- overlaps.rule.geneAssignment = gene_hierarcy
- overlaps.TranscriptRegions = "hierarchy"
- overlaps.rule.TranscriptRegions = transcript_region_hierarcy

The gene hierarchy is set to: "protein_coding" > "lncRNA" > "snRNA" > "snoRNA" > "miRNA" > "rRNA" > "misc_RNA" > "tRNA" > "pseudogene".

The transcript region hierarchy is set to: "INTRON" > "UTR5" > "UTR3" > "CDS".

# Check quality binding sites

The following shows the quality control plots for the binding sites for all 3 samples. 

```{r results="asis"}
bds <- readRDS(paste0(out, "bds.rds"))

for(i in 1:length(bds[1:3])){
  cat("##", names(bds)[i], "\n")
  print(quick_BSF_QC(bds[[i]], estimate.bs.width = F))
  cat("\n\n")
}


```


# Binding site characteristics

The following shows the binding site characteristics for all 3 samples.

```{r results="asis"}
for(i in 1:length(bds[1:3])){
  cat("##", names(bds)[i], "\n")
   print(quick_BSF_characterise(bds[[i]]))
   cat("\n\n")
}

```


# Session info

Here you can see the exact versions of the packages used for the analysis.

```{r}
sessionInfo()

```
```
